# *Funiliomycetaceae* fam. nov. (*Amphisphaeriales*, *Ascomycota*) accommodating *Funiliomyces*, including *F. jiangxiensis* sp. nov. from *Tetradium ruticarpum* and ten new combinations

**DOI:** 10.3897/imafungus.17.179140

**Published:** 2026-02-06

**Authors:** Li-Xue Mi, Dian-Ming Hu, Kevin D. Hyde, Prapassorn D. Eungwanichayapant, Ausana Mapook, Danushka S. Tennakoon, Jing-Yi Zhang, Hai-Yan Song

**Affiliations:** 1 School of Agricultural Sciences, Jiangxi Agricultural University, Nanchang 330045, China Bioengineering and Technological Research Centre for Edible and Medicinal Fungi, Jiangxi Agricultural University Nanchang China; 2 Bioengineering and Technological Research Centre for Edible and Medicinal Fungi, Jiangxi Agricultural University, Nanchang 330045, China Nanchang Key Laboratory of Edible and Medicinal Fungi, Jiangxi Agricultural University Nanchang China; 3 Nanchang Key Laboratory of Edible and Medicinal Fungi, Jiangxi Agricultural University, Nanchang 330045, China School of Agricultural Sciences, Jiangxi Agricultural University Nanchang China; 4 Center of Excellence in Fungal Research, Mae Fah Luang University, Chiang Rai 57100, Thailand Center of Excellence in Fungal Research, Mae Fah Luang University Chiang Rai Thailand; 5 School of Science, Mae Fah Luang University, Chiang Rai 57100, Thailand School of Science, Mae Fah Luang University Chiang Rai Thailand; 6 Shenzhen Key Laboratory of Microbial Genetic Engineering, College of Life Science and Oceanography, Shenzhen University, Shenzhen 518060, China College of Life Science and Oceanography, Shenzhen University Shenzhen China; 7 School of Food and Pharmaceutical Engineering, Guizhou Institute of Technology, Guiyang 550025, China School of Food and Pharmaceutical Engineering, Guizhou Institute of Technology Guiyang China

**Keywords:** *
Ascomycota
*, medicinal plant, multi-gene phylogeny, new family, *
Rutaceae
*, taxonomy

## Abstract

The genus *Dactylaria* has long been recognized as polyphyletic. In this study, phylogenetic analyses based on LSU sequences indicate that *Dactylaria* is widely distributed across six different classes within *Pezizomycotina*. Further analyses using combined LSU, ITS, and RPB2 sequence data revealed that *Funiliomyces biseptatus*, ten previously described “*Dactylaria*” species, and our newly isolated strains form a distinct lineage (SH-aLRT/UFB/BPP = 99.9/94/0.99) within *Amphisphaeriales*, sister to *Nothodactylariaceae*. Interfamily genetic differences provide additional evidence for recognizing this lineage as an independent family-level clade. Accordingly, a new family, *Funiliomycetaceae***fam. nov**., is proposed to accommodate this lineage. The majority of species in this family are saprobes, endophytes, or epiphytes, occurring on a wide range of plant hosts across tropical to temperate regions. The sexual morph of *Funiliomycetaceae* is characterized by black, subglobose ascomata; cylindrical asci with an IKI-negative apical apparatus; and pale brown, torpedo-shaped, 2-septate ascospores bearing two hyaline mucilaginous appendages. The asexual morph is characterized by macronematous conidiophores and integrated, sympodial conidiogenous cells that exhibit remarkable diversity in denticle morphology, ranging from large cylindrical or geniculate forms to entirely absent, producing hyaline to pale smoky, septate conidia of variable dimensions. The diversity of *Funiliomycetaceae* is expanded here by the addition of one new species, *Funiliomyces jiangxiensis***sp. nov**., and the transfer of ten previously described species to *Funiliomyces* as new combinations (*F. acaciae***comb. nov**., *F. bisepatus***comb. nov**., *F. calliandrae***comb. nov**., *F. fragilis***comb. nov**., *F. hwasunensis***comb. nov**., *F. mavisleverae***comb. nov**., *F. monticola***comb. nov**., *F. retrophylli***comb. nov**., *F. sparsus***comb. nov**., and *F. zapatensis***comb. nov**.). Information on asexual morphology, lifestyle, host associations, and distribution of *Funiliomyces* species is provided to facilitate species identification.

## Introduction

*Dactylaria* was established by Saccardo (1880) with *D. purpurella* as the type species, originally identified from dead leaves of *Castanopsis cuspidata* var. *sieboldii* in Japan. De Hoog (1985) expanded the circumscription of *Dactylaria*, recognizing 41 species classified into four sections: *Dactylaria*, *Mirandina* (G. Arnaud ex Matsush.) de Hoog, *Diplorhinotrichum* (Höhn.) de Hoog, and *Pleurophragmium* (Constantin) de Hoog. Later, Goh and Hyde (1997) expanded this framework by providing a taxonomic key for 37 additional species associated with plant material, increasing the number of recognized species to 82. Subsequent to Goh and Hyde’s (1997) review, a further 14 species have been described in *Dactylaria* (Paulus et al. 2003). The genus has undergone considerable taxonomic changes in recent years. Several new species have been described (Cooper 2005; Barbosa et al. 2013; Mel’nik et al. 2013; Magalhães et al. 2014; Crous et al. 2016; Crous et al. 2022; Tan and Shivas 2024; Crous et al. 2025; de Souza et al. 2025; Liu et al. 2025), while the application of molecular phylogenetic data has resulted in the transfer of some species to other genera, such as *Dactylaria hyalotunicata*, which is now recognized as *Pseudodactylaria hyalotunicata* (Crous et al. 2017). Conversely, species from other genera have been reassigned to *Dactylaria* based solely on traditional morphology, including *Subulispora argentina* and *S. malaysiana* (de Souza et al. 2025), although molecular evidence supporting these transfers is still lacking.

Currently, 134 epithets are listed under *Dactylaria* in Index Fungorum (2025) with 117 possible species listed in Species Fungorum (2025). Traditionally, members of *Dactylaria* are characterized by hyaline, cylindrical, rachis-bearing conidiogenous cells with apical, scattered, sympodial, tubular, or cylindrical denticles, and their conidia are hyaline, cylindrical, fusiform, or navicular, and can be unicellular or septate, with liberation occurring through schizolytic secession (de Hoog 1985; Goh and Hyde 1997; Paulus et al. 2003). *Dactylaria* species exhibit diverse ecological roles, with most species functioning as saprobes or parasites on a wide range of substrates including soil (Bhatt and Kendrick 2011) and plant materials (Paulus et al. 2003; Pinnoi et al. 2003; Senwanna et al. 2021; Wijesinghe et al. 2022). Additionally, some species act as endophytes that colonize living plant tissues (Wu et al. 1996), while others serve as nematode-trapping fungi (Krizková et al. 1976; Kumar and Singh 2011), plant pathogens, or opportunistic agents of animal diseases (Kralovic and Rhodes 1995; Goh and Hyde 1997).

To date, only 31 species have available sequence data in GenBank, with these data primarily corresponding to the LSU (large subunit ribosomal RNA gene) and SSU (small subunit ribosomal RNA gene) regions. No comprehensive, genus-wide phylogenetic study including the type species has been conducted and the genus’ systematic placement remains poorly resolved. Most critically, the phylogenetic position of its type species, *D. purpurella*, has yet to be clarified (Bussaban et al. 2005; Réblová 2009; Lin et al. 2021). LSU-based analyses conducted by Réblová (2009), which primarily addressed the sexual morph of *Rhodoveronaea* and the reevaluation of *Pleurophragmium*, demonstrated that *D. purpurella*, *D. monticola*, and *D. parvispora* occupy distinct lineages. The results excluded *Dactylaria* from synonymy with *Pleurophragmium* and further highlighted the polyphyletic nature of the genus. Despite its long taxonomic history, *Dactylaria* is taxonomically heterogeneous, leading to persistent controversy regarding its definition and classification (Goh and Hyde 1997; Paulus et al. 2003). Most species were historically established solely on morphological characteristics, particularly before the application of molecular tools (Goh and Hyde 1997; Cooper 2005; Flavia et al. 2013; Mel’nik et al. 2013; Magalhães et al. 2014). In recent years, with the advent of molecular techniques, newly described *Dactylaria* species have incorporated both morphological and molecular data, though typically limited to single-gene analyses. For example, *D. acaciae* (Crous et al. 2016), *D. retrophylli* (Crous et al. 2022), *D. calliandrae* (Crous et al. 2025), and *D. hwasunensis* (Liu et al. 2025) were identified using LSU sequences, whereas *D. mavisleverae* (Tan and Shivas 2024) was confirmed based on ITS sequences.

Interestingly, recent phylogenetic analyses of dactylaria-like taxa have revealed that *Funiliomyces biseptatus* consistently clusters with several “*Dactylaria*” species, forming a well-supported clade with uncertain taxonomic position within the order *Amphisphaeriales* (Crous et al. 2016, 2022, 2025). *Funiliomyces biseptatus*, the type and currently the only described species of the genus *Funiliomyces*, was established by Aptroot (2004) from a dead leaf of a *Bromeliaceae* plant. The genus is characterized by its fusiform ascospores with two submedian septa and furnished with both apical and median appendages. Notably, *F. biseptatus* represents a sexual morph, whereas the associated “*Dactylaria*” species are asexual forms. The close phylogenetic affinity between the sexual morph of *Funiliomyces* and asexual morphs of dactylaria-like taxa forms a robust, statistically well-supported clade that is phylogenetically distinct from all currently described families within *Amphisphaeriales*.

*Amphisphaeriales* was introduced by Eriksson and Hawksworth (1986). Subsequent multigene phylogenetic studies have consistently demonstrated that *Amphisphaeriales* and *Xylariales* represent distinct orders within the subclass *Xylariomycetidae* (Senanayake et al. 2015; Samarakoon et al. 2016; Hongsanan et al. 2017; Crous et al. 2018b). According to molecular dating analyses, the crown age of *Amphisphaeriales* has been estimated at around 133 MYA (Hyde et al. 2020). Members of *Amphisphaeriales* are characterized by gelatin-covered, interascal, tapering or reticulate paraphyses, host-immersed perithecia with or without a clypeus, hymenial asci with amyloid apices and apical rings, variable, hyaline or pigmented ascospores with variable septa, and holoblastic conidiogenesis with denticulate conidia and solitary or united conidiophores (McLaughlin and Spatafora 2014). The circumscription of *Amphisphaeriales* by Hyde et al. (2020) included 17 families: *Amphisphaeriaceae*, *Appendicosporaceae*, *Apiosporaceae*, *Beltraniaceae*, *Castanediellaceae*, *Clypeophysalosporaceae*, *Cylindriaceae*, *Hyponectriaceae*, *Iodosphaeriaceae*, *Melogrammataceae*, *Oxydothidaceae*, *Phlogicylindriaceae*, *Pseudomassariaceae*, *Pseudosporidesmiaceae*, *Pseudotruncatellaceae*, *Sporocadaceae*, and *Xyladictyochaetaceae*, while *Vialaeaceae* was excluded. Hyde et al. (2024) later incorporated *Vialaeaceae* into the order. In addition, two families originally placed in *Xylariales*, *Nothodactylariaceae*, and *Anungitiomycetaceae* (Crous et al. 2019b), have been transferred to *Amphisphaeriales* based on phylogenetic evidence (Réblová et al. 2021; Zhang et al. 2025).

Endophytic fungi associated with medicinal plants are well recognized as a rich reservoir of structurally diverse bioactive metabolites, and their interactions with host plants often contribute to the biosynthesis of pharmacologically important compounds, making them a focal point for natural product and fungal taxonomy research (Tiwari and Bae 2022; Wen et al. 2022; Gupta et al. 2023; Chandra et al. 2024). *Tetradium ruticarpum* (A.Juss.) Hartley, commonly known as *Evodia rutaecarpa* or Wu Zhu Yu in Chinese, is a medicinal plant of high value in traditional Chinese medicine (Chinese Pharmacopoeia Commission 2010). Its fruits are widely used for their pharmacological activities, including antitumor, cardiovascular protective, anti-inflammatory, antimicrobial, antioxidant, and anti-obesity effects (Li and Wang 2020; Xiao et al. 2023). Research on endophytic fungi associated with *T. ruticarpum* remains limited. Previously reported endophytes include *Sclerotium* Tode ex Fr., which produces multiple bioactive compounds (Zhu 2007), as well as other taxa identified only to the genus level, such as *Cyanodermella*, *Guignardia*, *Hypoxylon*, and *Nigrospora* (Ho et al. 2012). Two novel species, *Cyphellophora guangxiensis* (Mi et al. 2025b) and *Pseudokeissleriella tetradii* (Mi et al. 2025a), were recently reported from *T. ruticarpum*.

In this study, we elucidate the long-recognized polyphyly of *Dactylariasensu lato*. Phylogenetic analyses based on the LSU gene show that species historically assigned to this genus, for which LSU sequence data are available, are distributed across at least six distinct classes within *Pezizomycotina*, further supporting its non-monophyletic status. Phylogenetic inference using a concatenated LSU-ITS-RPB2 dataset and interfamily genetic differences recover a monophyletic lineage within *Amphisphaeriales*, comprising *Funiliomyces biseptatus*, ten species previously classified under *Dactylaria*, and our newly isolated strains. We thus formally propose the new family *Funiliomycetaceae* fam. nov. to accommodate this well-delimited clade. Additionally, we describe one new species *Funiliomyces jiangxiensis* from *Tetradium ruticarpum*, formally transfer the ten aforementioned *Dactylaria* species to *Funiliomyces* as new combinations, and present a table summarizing the asexual morphological features, lifestyle, host associations, and distribution of all known *Funiliomyces* species.

## Materials and methods

### Collection, isolation, and morphological studies

The healthy roots of *Tetradium ruticarpum* were collected from Jiangxi Province, China. The samples were placed into sterile self-sealing plastic bags, transported to the lab and stored at 4 °C until processing. Tissue isolation and culture method were carried out by following the method of Deng et al. (2014). The specific disinfection time was adjusted according to *T. ruticarpum*. The roots were repeatedly washed by using running tap water and air dried. The surface of each sample was disinfected using 75% ethanol for 1 min, and 3.3% sodium hypochlorite solution for 1 min, followed by three rinses in sterile distilled water before finally being dried on sterile filter papers. After disinfection, the roots were cut into small pieces (ca. 5 mm^2^) by a sterile scalpel and placed in Potato Dextrose Agar plates (PDA), incubated at 25 °C in darkness. When the fungi develop from the tissue segment, a few hyphal fragments were picked up and transferred to new PDA plates to obtain pure cultures.

Micromorphological characteristics from pure cultures were observed and captured using a Nikon ECLIPSE Ni-U compound microscope (Nikon Corporation, Japan), equipped with a Nikon DS-Fi3 camera. All measurements were calculated using PhotoRuler v. 1.1 software (The Genus Inocybe, Hyogo, Japan). For conidiophores and conidia, morphological data are presented as range followed by mean ± standard deviation (SD). The figures were processed using Adobe Photoshop CS6 Extended v.10.0 software (Adobe Systems, USA). The fungal cultures were deposited in the Jiangxi Agricultural University Culture Collection (JAUCC), Nanchang, China, and the China Center for Type Culture Collection (CCTCC), respectively. The dry cultures were deposited in the Herbarium of Fungi, Jiangxi Agricultural University (HFJAU), Nanchang, China. The newly introduced fungal species was submitted to the Index Fungorum (2025) and Faces of Fungi database (Jayasiri et al. 2015).

### DNA extraction, PCR amplification and sequencing

Fresh mycelium was extracted using the CTAB method (Doyle and Doyle 1987). According to the locus-specific databases of the *Dactylaria* species (Crous et al. 2016; Crous et al. 2022), three loci (LSU, ITS, and RPB2) were chosen for polymerase chain reaction (PCR) using the primer pairs LR0R/LR5 (Vilgalys and Hester 1990), ITS1/ITS4 (White et al. 1990), and fRPB2-5F/fRPB2-7CR (Liu et al. 1999), respectively. The final volume of the PCR reaction mixture was 25 μL, contained 12.5 μL 2×Taq PCR MasterMix (Qingke, Changsha, China), 1 μL each forward and reverse primer (0.2 μM), 1 μL template DNA (circa 50–100 ng), and 9.5 μL ddH_2_O. For ITS and LSU genes, the PCR parameters were initial denaturation 94 °C for 5 min; followed by 35 cycles consisting of denaturation at 94 °C for 30 seconds, annealing at 55 °C for 50 seconds and extension at 72 °C for 60 seconds; and final extension at 72 °C for 10 min. For RPB2 gene, the PCR parameters were initial denaturation 94 °C for 5 min; followed by 40 cycles consisting of denaturation at 94 °C for 60 seconds, annealing at 58 °C for 60 seconds and extension at 72 °C for 60 seconds; and a final extension at 72 °C for 10 min. The PCR products were purified and sequenced by QingKe Biotechnology Co. (Changsha, China). All sequences were assembled using BioEdit v.7.2.6 (Hall 1999). The new sequences were deposited in GenBank (https://www.ncbi.nlm.nih.gov/). The sequence alignments and resulting phylogenetic trees have been deposited in TreeBASE (www.treebase.org).

### Reference datasets

We combined the new ribosomal and protein coding sequences with data from the National Center for Biotechnology Information (NCBI). Accession numbers for all sequences used for the molecular analyses are provided in Tables 1, 2.

**Table 1.** Taxa, isolate information, and GenBank accession numbers for sequences used in the phylogenetic analyses of *Pezizomycotina*.

Taxon	Strain	GenBank accession numbers	References
		LSU	
*Acarosporina microspora*	CBS 338.39	AY584643	Lutzoni et al. (2004)
*Amphisphaeria umbrina*	HKUCC 994	AF452029	Jeewon et al. (2003)
*Anungitiomyces stellenboschiensis*	CPC 34726^T^	NG_067874	Crous et al. (2019a)
*Appendicospora hongkongensis*	HKAS 107015	MW240581	Samarakoon et al. (2022)
*Arthonia dispersa*	UPSC 2583	AY571381	Lumbsch et al. (2005)
*Arthrobotrys macroides*	CBS 120.54	MH868799	Vu et al. (2019)
*Arthrobotrys mangrovispora*	MGDW17	EU573355	Swe et al. (2008)
*Ascosphaera apis*	CBS 402.96	FJ358275	Gueidan et al. (2008)
*Aspergillus fumigatus*	INFU/Jc/KF/6	FM179606	Kusari et al. (2009)
*Barrmaelia serenoae*	CPC:37572^T^	MT223876	Crous et al. (2020)
*Caliciopsis pinea*	CBS 139.64	DQ678097	Schoch et al. (2006b)
*Camarops ustulinoides*	AFTOL-ID 72	DQ470941	Spatafora et al. (2006)
*Candelaria concolor*	AFTOL-ID 1706	DQ986791	Miadlikowska et al. (2006)
*Candelariella reflexa*	AFTOL-ID 1271	DQ912331	Miadlikowska et al. (2006)
*Candelariella terrigena*	AFTOL-ID 227	DQ986745	Miadlikowska et al. (2006)
*Candida albicans*	ATCC 18804^T^	NG_054826	Suh et al. (2013)
*Capnodium coffeae*	CBS 147.52	DQ247800	Schoch et al. (2006a)
*Chaenotheca furfuracea*	Wedin 6366 (UPS)^T^	JX000087	Prieto et al. (2013)
*Cirrosporium novae-zelandiae*	CBS 125236	HQ878612	Réblová et al. (2012)
*Cladonia caroliniana*	AFTOL-ID 3	AY584640	Lutzoni et al. (2004)
*Cladosporium herbarum*	CBS 121621	MH874676	Vu et al. (2019)
*Coccomyces strobi*	AFTOL-ID 1250	DQ470975	Spatafora et al. (2006)
*Collemopsidium angermannicum*	s1473	KU556871	Pérez-Ortega et al. (2016)
*Collemopsidium pelvetiae*	RO25	KU556868	Pérez-Ortega et al. (2016)
*Conlarium baiseense*	TD17	MK164655	Xie et al. (2019)
*Cordyceps militaris*	CBS 110.70	MH878247	Vu et al. (2019)
*Corynelia africana*	PREM 57242^T^	NG_058910	Wood et al. (2016)
*Dactylaria acicularis*	CBS 511.72	MH872253	Vu et al. (2019)
*Dactylaria aquatica*	P003	EU107312	Bhilabutra et al. (2007, submitted directly)
*Dactylaria belliana*	P020	EU107295	Bhilabutra et al. (2007, submitted directly)
*Dactylaria dimorpha*	P067	EU107305	Bhilabutra et al. (2007, submitted directly)
*Dactylaria dimorphospora*	CBS 256.70	MH871358	Vu et al. (2019)
*Dactylaria flammulicornuta*	P005	EU107313	Bhilabutra et al. (2007, submitted directly)
*Dactylaria humicola*	P006	EU107304	Bhilabutra et al. (2007, submitted directly)
*Dactylaria hyalotunicata*	P010	EU107298	Bhilabutra et al. (2007, submitted directly)
*Dactylaria longispora*	P001	EU107299	Bhilabutra et al. (2007, submitted directly)
*Dactylaria lunata*	CBS 689.93	MH874102	Vu et al. (2019)
*Dactylaria parvispora*	P024	EU107296	Bhilabutra et al. (2007, submitted directly)
*Dactylaria purpurella*	P048	EU107301	Bhilabutra et al. (2007, submitted directly)
*Dactylaria sahelensis*	CBS 247.93	MH874055	Vu et al. (2019)
*Dactylaria simonensis*	CBS 367.90	MH873904	Vu et al. (2019)
*Dactylaria sporexamorpha*	CBS 690.93	MH874103	Vu et al. (2019)
*Dactylaria uliginicola*	P007	EU107311	Bhilabutra et al. (2007, submitted directly)
*Dactylellina appendiculata*	CBS 206.64^T^	NG_059071	Li et al. (2006)
*Dactylellina leptospora*	CBS 560.92	AY261162	Crous et al. (2022)
*Dactylospora haliotrepha*	AFTOL-ID 758	FJ176855	Schoch et al. (2009a)
*Dactylospora mangrovei*	AFTOL-ID 2108	FJ176890	Schoch et al. (2009a)
*Delphinella strobiligena*	AFTOL-ID 1257	DQ470977	Spatafora et al. (2006)
*Dendrographa minor*	R. Ornduff 10070	AF279382	Bhattacharya et al. (2000)
*Diatrype disciformis*	AFTOL-ID 927	DQ470964	Spatafora et al. (2006)
*Dothiora cannabinae*	CBS 737.71	DQ470984	Spatafora et al. (2006)
*Elaphomyces granulatus*	AFTOL-ID 436^T^	KT232217	Voglmayr et al. (2019)
*Eremascus albus*	CBS 975.69	MH871279	Voglmayr et al. (2019)
*Exophiala dermatitidis*	AFTOL-ID 668^T^	DQ823100	Voglmayr et al. (2019)
*Funiliomyces acaciae*	CPC 29771^T^	KY173493	Crous et al. (2016)
*Funiliomyces biseptatus*	P062	EU107288	Bhilabutra et al. (2007, submitted directly)
*Funiliomyces calliandrae*	CPC 48004^T^	PV664963	Crous et al. (2025)
*Funiliomyces fragilis*	P057	EU107290	Bhilabutra et al. (2007, submitted directly)
*Funiliomyces hwasunensis*	CMML 20-35	PQ741487	Liu et al. (2025)
*Funiliomyces jiangxiensis*	CCTCC AF2023034^T^	OQ869216	This study
*Funiliomyces mavisleverae*	BRIP 76362a^T^	PQ431199	Tan and Shivas (2024)
*Funiliomyces monticola*	P060	EU107289	Bhilabutra et al. (2007, submitted directly)
*Funiliomyces retrophylli*	CBS:148271	ON811548	Crous et al. (2022)
*Funiliomyces sparsus*	P055	EU107291	Bhilabutra et al. (2007, submitted directly)
*Funiliomyces zapatensis*	P056	EU107287	Bhilabutra et al. (2007, submitted directly)
*Fusarium graminearum*	CBS 132906	MH877513	Vu et al. (2019)
*Gelasinospora tetrasperma*	CBS 178.33	DQ470980	Spatafora et al. (2006)
*Geoglossum nigritum*	AFTOL-ID 56^T^	AY544650	Schoch et al. (2009b)
*Halosphaeria appendiculata*	CBS 197.60	MH869504	Vu et al. (2019)
*Herpomyces chaetophilus*	D. Haelew. 1097b	MG438352	Haelewaters et al. (2019b)
*Hypocrea lutea*	ATCC 208838	AF543791	Currie et al. (2003)
*Hyponectria buxi*	UME 31430	AY083834	Aptroot et al. (2004)
*Laboulbenia bruchii*	D. Haelew 1346b	MN394843	Haelewaters et al. (2019a)
*Lecanora contractula*	AFTOL-ID 877	DQ986746	Miadlikowska et al. (2006)
*Lecanora paramerae*	Lumbsch s.n. (F)	EF105422	Crespo et al. (2007)
*Lempholemma polyanthes*	Zoladeski and Lutzoni 11294-L1(2/2) (CANL)	AF356691	Lutzoni et al. (2001)
*Lepraria lobificans*	AFTOL-ID 325	DQ986768	Miadlikowska et al. (2006)
*Lichina confinis*	I1024	MT877184	Garrido-Benavent (2020, submitted directly)
*Lichina pygmaea*	I1028	MT877182	Garrido-Benavent (2020, submitted directly)
* Marcelleina persoonii *	AFTOL-ID 164	DQ470943	Spatafora et al. (2006)
*Microascus longirostris*	CBS 196.61	NG_058479	Daros-Pawlyta and Pawlyta (2023)
*Mycocalicium subtile*	Wedin 6889 (UPS)^T^	AY853379	Wedin et al. (2005)
*Mycosphaerella etlingerae*	CBS 129062	NG_069080	Crous et al. (2011b)
*Myrmecridium schulzeri*	CBS 188.96^T^	EU041829	Arzanlo et al. (2007)
*Nectria cinnabarina*	CBS_114055	KU382228	Zare and Gams (2016)
*Neomyrmecridium asiaticum*	CBS:145080	NG_066291	Crous et al. (2018a)
*Nothodactylaria nephrolepidis*	CPC 37028^T^	NG_068668	Crous et al. (2019b)
*Orbilia albidorosea*	CBS 140818^T^	NG_073646	Vu et al. (2019)
*Orbilia vinosa*	AFTOL-ID 905^T^	DQ470952	Spatafora et al. (2006)
*Peltula umbilicata*	AFTOL-ID 891	DQ832334	James et al. (2006)
*Petriella setifera*	AFTOL-ID 956	DQ470969	Spatafora et al. (2006)
*Peziza varia*	JC19031901	MT247057	Van Vooren (2020)
*Peziza vesiculosa*	AFTOL-ID 507	DQ470948	Spatafora et al. (2006)
*Plicaria leiocarpa*	CBS 144.92	DQ842029	Spatafora et al. (2006)
*Pseudodactylaria xanthorrhoeae*	CBS:143414	NG_058521	Crous et al. (2017)
*Pseudotruncatella arezzoensis*	MFLUCC 14-0988^T^	NG_070426	Perera et al. (2018)
*Pyrenula reebiae*	DUKE:0047599^T^	NG_068722	Reeb et al. (2004)
*Ramichloridium anceps*	AFTOL-ID 659^T^	DQ823102	James et al. (2006)
*Roccella fuciformis*	AFTOL-ID 126^T^	AY584654	Lutzoni et al. (2004)
*Roccellographa cretacea*	AFTOL-ID 93	DQ883696	Spatafora et al. (2006)
*Sabahriopsis eucalypti*	CPC:24957	NG_058167	Crous et al. (2015b)
*Saccharomyces cerevisiae*	NRRL Y-12632^T^	NG_042623	Kurtzman et al. (2013)
*Sarea difformis*	JR6451^T^	MN938401	Beimforde et al. (2020)
*Sarea resinae*	JR6450	MN938402	Beimforde et al. (2020)
*Schismatomma decolorans*	Ertz 5003 (BR)	AY548815	Lutzoni et al. (2004)
*Sclerophora farinacea*	Wedin 6414 (UPS)	JX000095	Prieto et al. (2013)
*Sordaria fimicola*	SMH4106	AY780079	Miller and Huhndorf (2005)
*Spathularia velutipes*	AFTOL-ID 1291	FJ997861	Voglmayr et al. (2019)
* Sphinctrina turbinata *	AFTOL-ID 1721	EF413632	Geiser et al. (2006)
*Teloschistes exilis*	AFTOL-ID 87	AY584647	Lutzoni et al. (2004)
*Toxicocladosporium chlamydosporum*	CBS 124157^T^	NG_069916	Vu et al. (2019)
*Trapelia placodioides*	KS163	KU844623	Schneider et al. (2016)
*Trichocoma paradoxa*	CBS 788.83	FJ358290	Gueidan et al. (2008)
*Trichoglossum hirsutum*	AFTOL-ID 64	AY544653	Voglmayr et al. (2019)
*Trinosporium guianense*	CBS 132537^T^	NG_042682	Crous et al. (2012)
*Tryblidiopsis pinastri*	AFTOL-ID 1319	DQ470983	Spatafora et al. (2006)
*Umbilicaria arctica*	AFTOL-ID 1266	DQ986772	Miadlikowska et al. (2006)
*Umbilicaria hyperborea*	OSC:McCune 35477	MH290350	McCune (2018)
*Volutella ciliata*	CBS 127312	MH875955	Vu et al. (2019)
*Xylaria acuta*	AFTOL-ID 63	AY544676	Hongsanan and Hyde (2017)
*Xylaria hypoxylon*	AFTOL-ID 51	AY544648	Voglmayr et al. (2019)
*Xylobotryum andinum*	XA1	MH468791	Voglmayr et al. (2019)
*Xylobotryum portentosum*	XP	MH468792	Voglmayr et al. (2019)
*Xylona heveae*	CBS 132557^T^	NG_066160	Gazis et al. (2012)

Notes: New sequences determined for this study and taxonomic novelties are given in light orange. New combinations are given in blue. Superscript T denotes ex-type strains.

**Table 2.** Taxa, isolate information, and GenBank accession numbers for sequences used in the phylogenetic analyses of *Amphisphaeriales*.

Taxon	Strain	GenBank accession numbers			References
		LSU	ITS	RPB2	
*Achaetomium macrosporum*	CBS 532.94	KX976699	KX976574	KX976797	Wang et al. (2016)
*Amphisphaeria fuckelii*	CBS:140409^T^	KT949902	KT949902	MH554918	Jaklitsch et al. (2016)
*Amphisphaeria thailandica*	MFLU 18-0794^T^	MH971235	MH971225	MK033640	Samarakoon et al. (2019)
*Anungitiomyces stellenboschiensis*	CPC:34726^T^	MK876415	MK876376	NA	Crous et al. (2019b)
*Appendicospora hongkongensis*	HKAS 107015	MW240581	MW240651	MW658638	Samarakoon et al. (2022)
*Arthrinium hysterinum*	ICMP 6889	MK014841	MK014874	DQ368649	Pintos et al. (2019)
*Arthrinium pseudoparenchymaticum*	SICAUCC 18-0008	MK346321	MK346319	MK359207	Yang et al. (2019)
*Beltrania pseudorhombica*	CBS:138003^T^	KJ869215	MH554124	MH555032	Crous et al. (2014a)
*Beltraniopsis neolitseae*	CBS:137974^T^	KJ869183	KJ869126	NA	Crous et al. (2014a)
*Brachiampulla verticillata*	ICMP 15993	MW144403	MW144419	NA	Réblová et al. (2021)
*Castanediella acaciae*	CBS 139896^T^	MH878661	KR476728	NA	Vu et al. (2019)
*Castanediella cagnizarii*	MUCL 41095	KC775707	KC775732	NA	Becerra-Hernández et al. (2016)
*Castanediella ramosa*	MUCL 39857	KC775711	KC775736	NA	Becerra-Hernández et al. (2016)
*Chaetomium elatum*	CBS 374.66	MH870466	KC109758	KF001820	Vu et al. (2019)
*Clypeophysalospora latitans*	CBS 141463^T^	KX820261	KX820250	NA	Giraldo et al. (2017)
*Cylindrium elongatum*	CBS 115974	KM231733	KM231853	KM232429	Lombard et al. (2015)
*Cylindrium grande*	CBS:145578	MK876426	MK876385	MK876482	Crous et al. (2019a)
*Funiliomyces acaciae*	CPC 29771	KY173493	KY173400	NA	Crous et al. (2016)
*Funiliomyces biseptatus*	P062	EU107288	NA	NA	Bhilabutra et al. (2007, submitted directly)
*Funiliomyces biseptatus*	CBS 100373^T^	NG_067443	NR_159862	NA	Aptroot (2004)
*Funiliomyces calliandrae*	CPC 48004	PV664963	PV664937	PV664022	Crous et al. (2025)
*Funiliomyces fragilis*	P057	EU107290	NA	NA	Bhilabutra et al. (2007, submitted directly)
*Funiliomyces hwasunensis*	CMML 20-35	PQ741487	PQ741486	NA	Liu et al. (2025)
*Funiliomyces hwasunensis*	CMML 20-88	PQ741488	NA	NA	Liu et al. (2025)
*Funiliomyces jiangxiensis*	CCTCC AF2023034^T^	OQ869216	OQ869213	OR046688	This study
*Funiliomyces jiangxiensis*	CCTCC AF2023033	OQ869214	OQ869215	OR046687	This study
*Funiliomyces mavisleverae*	BRIP 76362a	PQ431199	PQ431206	NA	Tan and Shivas (2024)
*Funiliomyces monticola*	P060	EU107289	NA	NA	Bhilabutra et al. (2007, submitted directly)
*Funiliomyces retrophylli*	CBS:148271	ON811548	ON811489	NA	Crous et al. (2022)
*Funiliomyces sparsus*	P055	EU107291	NA	NA	Bhilabutra et al. (2007, submitted directly)
*Funiliomyces zapatensis*	P056	EU107287	NA	NA	Bhilabutra et al. (2007, submitted directly)
*Hyponectria buxi*	UME 31430	AY083834	NA	NA	Smith et al. (2002, submitted directly)
*Iodosphaeria honghensis*	MFLU 19-0719^T^	MK722172	MK737501	MK791287	Marasinghe et al. (2019)
*Iodosphaeria tongrenensis*	MFLU:15-0393	KR095283	KR095282	NA	Li et al. (2015)
*Leiosphaerella praeclara*	CBS:125586	JF440976	JF440976	NA	Jaklitsch and Voglmayr (2012)
*Melogramma campylosporum*	MFLU 17-0348	MW240575	MW240645	MW658632	Samarakoon et al. (2022)
*Melogramma campylosporum*	MFLU 18-0778	MW240576	MW240646	MW658633	Samarakoon et al. (2022)
*Neoamphisphaeria hyalinospora*	MFLU 19-2131^T^	MW240579	MW240649	MW658636	Samarakoon et al. (2022)
*Neoamphisphaeria shangrilaensis*	HKAS 130274	PP584800	PP584703	NA	Dissanayake et al. (2024)
*Neophysalospora eucalypti*	CBS:138864^T^	KP004490	KP004462	NA	Crous et al. (2014b)
*Nothodactylaria comitabilis*	CPC 45173	PQ498974	PQ498925	NA	Crous et al. (2024)
*Nothodactylaria fusiformis*	KUNCC:23-13961^T^	PQ671162	PQ671242	PQ662509	Zhang et al. (2025)
*Nothodactylaria guizhouensis*	KUNCC:23-14080^T^	PQ671163	PQ671243	PQ662510	Zhang et al. (2025)
*Nothodactylaria nephrolepidis*	CPC:37028	MN567639	MN562132	MN556809	Crous et al. (2019b)
*Nothodactylaria nephrolepidis*	CBS:146078^T^	NG_068668	NR_166331	NA	Crous et al. (2019b)
*Nothodactylaria polyblastis*	KUNCC:23-13922^T^	PQ671164	PQ671244	PQ662511	Zhang et al. (2025)
*Nothodactylaria woodwardiae*	KUNCC:23-13927^T^	PQ671165	PQ671245	PQ662512	Zhang et al. (2025)
*Nothodactylaria woodwardiae*	KUNCC:23-13886	PQ671166	PQ671246	PQ662513	Zhang et al. (2025)
*Nothodactylaria woodwardiae*	KUNCC:23-14045	PQ671167	PQ671247	PQ662514	Zhang et al. (2025)
*Nothodactylaria woodwardiae*	KUNCC:23-13954	PQ671168	PQ671248	NA	Zhang et al. (2025)
*Nothodactylaria woodwardiae*	KUNCC23-14006	PQ671169	PQ671249	NA	Zhang et al. (2025)
*Oxydothis metroxyli*	MFLUCC15-0283	KY206764	KY206775	NA	Konta et al. (2016)
*Oxydothis metroxylonicola*	MFLUCC 15-0281^T^	KY206763	KY206774	KY206781	Konta et al. (2016)
*Oxydothis palmicola*	MFLUCC 15-0806^T^	KY206765	KY206776	KY206782	Konta et al. (2016)
*Phlogicylindrium eucalypti*	CBS 120080^T^	DQ923534	DQ923534	MH554893	Summerell et al. (2006)
*Phlogicylindrium uniforme*	CBS 131312^T^	JQ044445	JQ044426	NA	Crous et al. (2011a)
*Polyscytalum eucalyptorum*	CPC 17207^T^	KJ869176	KJ869118	NA	Crous et al. (2014a)
*Pseudapiospora corni*	WU:36852^T^	KT949907	KT949907	NA	Jaklitsch et al. (2016)
*Pseudomassaria chondrospora*	CBS:125600	JF440981	JF440981	NA	Jaklitsch and Voglmayr (2012)
*Pseudosporidesmium knawiae*	CBS:123529^T^	MH874823	MH863299	NA	Vu et al. (2019)
*Pseudosporidesmium lambertiae*	CBS:143169^T^	MG386087	MG386034	NA	Crous et al. (2017)
*Pseudotruncatella arezzoensis*	MFLUCC:14-0988^T^	MG192317	MG192320	NA	Perera et al. (2018)
*Pseudotruncatella bolusanthi*	CBS:145532^T^	MK876448	MK876407	NA	Crous et al. (2019a)
*Robillarda sessilis*	CBS 114312^T^	KR873284	KR873256	NA	Crous et al. (2015a)
*Seiridium marginatum*	CBS:140403^T^	KT949914	KT949914	MK523301	Jaklitsch et al. (2016)
*Sordaria fimicola*	CBS 723.96	NA	MH862606	NA	Vu et al. (2019)
*Strelitziomyces knysnanus*	CPC:37067^T^	MN567642	MN562135	MN556810	Crous et al. (2019b)
*Subsessila turbinata*	MFLUCC:15-0831^T^	KX762289	KX762288	NA	Lin et al. (2017)
*Vialaea insculpta*	DAOM:240257	JX139726	JX139726	NA	Hambleton et al. (2010, submitted directly)
*Vialaea minutella*	BRIP:56959	KC181924	KC181926	NA	McTaggart et al. (2013)
*Xyladictyochaeta lusitanica*	CBS:142290^T^	KY853543	KY853479	NA	Hernández-Restrepo et al. (2017)

Notes: New sequences determined for this study and taxonomic novelties are given in light orange. New combinations are given in blue. Superscript T denotes ex-type strains. NA indicates the unavailability of data.

1. *Pezizomycotina*: To determine the phylogenetic placement of *Dactylaria* within *Pezizomycotina*, we compiled a dataset representing all major ascomycete classes and conducted LSU single-gene analyses (Beimforde et al. 2020). Of the 31 *Dactylaria* species with molecular data in GenBank, five (*D. acerosa*, *D. appendiculata*, *D. candidula*, *D. junci*, and *D. lanosa*) lack LSU sequences and were excluded. The final dataset included 124 species, comprising 26 *Dactyla­ria* species. Accession numbers for all sequences are provided in Table 1.

2. *Amphisphaeriales*: To assess the placement of our two new strains, 71 taxa were selected based on the *Pezizomycotina* phylogeny of *Dactylaria* and recent publications (Crous et al. 2016, 2022, 2025; Tan and Shivas 2024; Liu et al. 2025). Phylogenetic analyses of *Dactylaria* species affiliated with *Amphisphaeriales* were performed using LSU, ITS, and RPB2 sequences. Accession numbers for all sequences are listed in Table 2.

### Phylogenetic analyses

Phylogenetic analyses were conducted using Maximum Likelihood (ML) and Bayesian Inference (BI). Sequences for both datasets were obtained from GenBank (http://www.ncbi.nlm.nih.gov/). All sequences were aligned with the online version of MAFFT v. 7 (https://mafft.cbrc.jp/alignment/server/) (Katoh and Standley 2013) and manually refined in BioEdit v. 7.2.6 (Hall 1999), with gaps treated as missing data. Dataset 1 contained only LSU sequences, whereas for Dataset 2, LSU, ITS, and RPB2 sequences were concatenated into a supermatrix using PhyloSuite v. 1.2.2 (Zhang et al. 2020). Alignment file formats were subsequently converted with ALTER (Alignment Transformation Environment; http://www.sing-group.org/ALTER/).

ML analyses were carried out on the dataset using IQ-TREE v.1.6 (http://iqtree.cibiv.univie.ac.at/) with partitioning, and the optimal substitution model for each partition was determined by IQ-TREE. (Trifinopoulos et al. 2016). Clade support for the ML analyses was assessed using the Shimodaira-Hasegawa-like approximate likelihood ratio test (SH-aLRT) with 1,000 replicates (Guindon et al. 2010) and 1,000 replicates of the ultrafast bootstrap (UFB) (Hoang et al. 2017). Nodes with support values of both SH-aLRT ≥ 80 and UFB ≥ 95 were considered supported, nodes with one of SH-aLRT ≥ 80 or UFB ≥ 95 were weakly supported, and nodes with both SH-aLRT < 80 and UFB < 95 were unsupported, and other parameters were used for the default settings (Liu et al. 2022).

Bayesian Inference (BI) analyses were performed on the CIPRES Science Gateway (http://www.phylo.org/) (Miller et al. 2010) using MrBayes 3.2.7 (Ronquist et al. 2012). For both datasets, each gene partition was analyzed under the GTR+I+G model, which was selected as the best-fit model using MrModeltest 2.3 in PAUP* v4.0b (Swofford 2002; Nylander 2004). Two independent Markov Chain Monte Carlo (MCMC) runs were conducted for 10,000,000 generations, sampling every 1,000 generations, with the first 25% of samples discarded as burn-in. Convergence was assessed by ensuring the average standard deviation of split frequencies (ASDSF) fell below 0.01. Bayesian posterior probabilities (BPP) were used to assess node support (Rannala and Yang 1996). Resulting trees were visualized in FigTree v. 1.4.4 (Rambaut 2018) and edited using the online service tvBOT (https://www.chiplot.online/tvbot.html) (Xie et al. 2023). Genetic distances were estimated using the Kimura 2-parameter (K2P) model in MEGA-X (Kumar et al. 2018) based on ITS, LSU, and RPB2 sequences of species representing different family within *Amphisphaeriales*.

## Results

### Molecular analysis

Based on a nucleotide BLAST search in GenBank database using the LSU sequence, the closest matches are *Dactylaria* species, including *Funiliomyces hwasunensis* (≡ *D. calliandrae*) CMML 20-35 [GenBank PQ741487; identities = 820/824 (99%), gaps = 0/824 (0%)], *F. calliandrae* (≡*D. calliandrae*) CPC 48004 [GenBank PV664963; identities = 805/811 (99%), gaps = 0/811 (0%)], *F. fragilis* (≡ *D. fragilis*) P057 [GenBank EU107290; identities = 798/807 (99%), gaps = 0/811 (0%)]. The closest match for the ITS sequence is *Fusidium griseum* Trtsf08 [GenBank GU479905; identities = 468/495 (95%), gaps = 7/495 (1%)], *D. acerosa* ICMP 13178 [GenBank OR543730; identities = 465/494 (94%), gaps = 4/494 (4%)], and *F. calliandrae* (≡ *D. calliandrae*) CPC 48004 [GenBank PV664937; identities = 454/479 (95%), gaps = 6/479 (4%)]. For the RPB2 sequence, the closest matches included *Dicyma funiculosa* CBS 323.86 [GenBank KU684306; identities = 579/735 (79%), gaps = 6/735 (0%)], and *Xylaria liquidambaris* FCATAS879 [GenBank MZ707110; identities = 553/700 (79%), gaps = 8/700 (1%)]. This situation may be due to the limited availability of RPB2 gene sequences for this genus.

### Phylogenetic analysis

**Figure 1.**
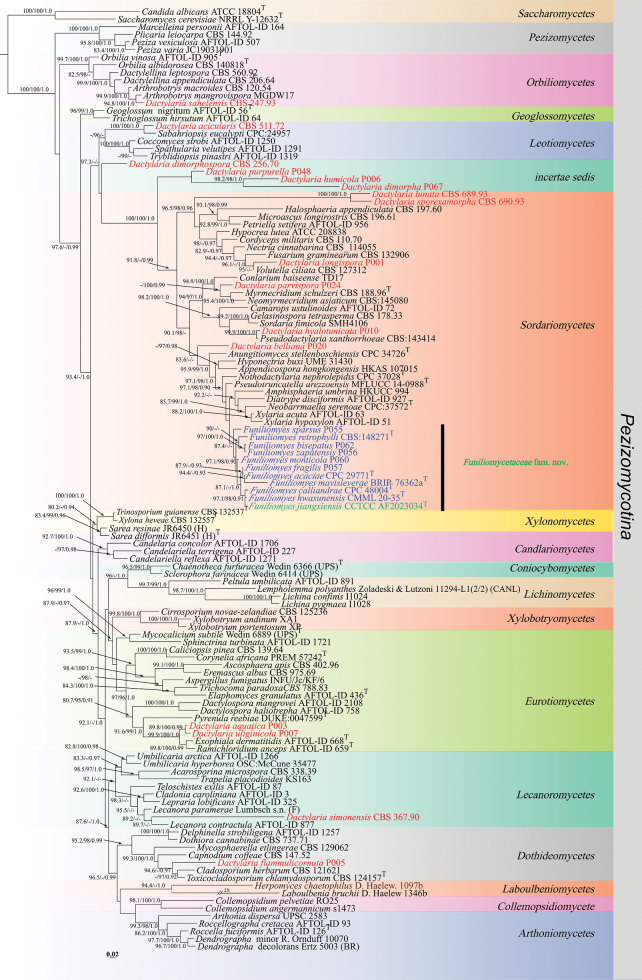
Maximum likelihood phylogenetic tree of *Pezizomycotina* based on LSU sequences, with Shimodaira-Hasegawa-like approximate likelihood ratio test (SH-aLRT) (left), ultrafast bootstrap (UFB) (middle), and Bayesian posterior probabilities (BPP) values (right) near the corresponding node. Only one of SH-aLRT > 80 or UFB > 95 for ML and BYPP > 0.90 for BI is indicated along the branches (SH-aLRT/UFB/BPP). The “*Dactylaria*” species are indicated in red, the newly generated sequences are indicated in green, and species for reclassification from *Dactylaria to Funiliomyces* are in blue. Ex-type strains are marked with T after the strain number. The ML phylogram is available in TreeBASE (study accession S32460; http://purl.org/phylo/treebase/phylows/study/TB2:S32460).

The phylogenetic hypothesis inferred from the LSU gene dataset for *Pezizomycotina* is presented in Fig. 1, with *Candida albicans* (ATCC 18804) and *Saccharomyces cerevisiae* (NRRL Y-12632) selected as outgroup taxa to root the tree, following previous large-scale phylogenetic studies of *Pezizomycotina* (Beimforde et al. 2020). The LSU alignment comprised 898 bp (including gaps), with 445 parsimony-informative sites, 94 singletons, and 359 constant sites. Maximum likelihood (ML) analysis recovered 629 distinct alignment patterns, with 7.33% undetermined characters or gaps. The best-scoring ML tree (–lnL = 22860.942) is shown in Fig. 1. Overall, the topologies obtained from ML and Bayesian inference (BI) analyses were highly congruent, differing only in the placement of a few taxa and in several unresolved clades. In this analysis, twenty-two *Dactylaria* species are distributed across six different classes within *Pezizomycotina*, namely *Orbiliomycetes* (one species), *Leotiomycetes* (one species), *Eurotiomycetes* (two species), *Sordariomycetes* (16 species), *Dothideomycetes* (one species), and *Lecanoromycetes* (one species). The remaining four species could not be confidently assigned to any currently recognized class of *Pezizomycotina*. The phylogenetic tree reveals that *Dactylaria* is polyphyletic, with most species clustering in *Sordariomycetes*. The exact taxonomic position of the type species, *D. purpurella*, remains uncertain. It forms a distinct lineage with *D. humicola* and *D. dimorpha*, representing an *incertae sedis* lineage in *Pezizomycotina*. In addition, our newly isolated strain clustered in the *Sordariomycetes* clade with ten species of *Funiliomyces* (formerly treated as *Dactylaria*) in *Funiliomycetaceae* fam. nov. with branch support values of SH-aLRT = 87.4%, UFB = 94%, and BPP = 0.88 (UFB value < 95% and BPP < 0.90 are not shown on the tree) (Fig. 1).

**Figure 2.**
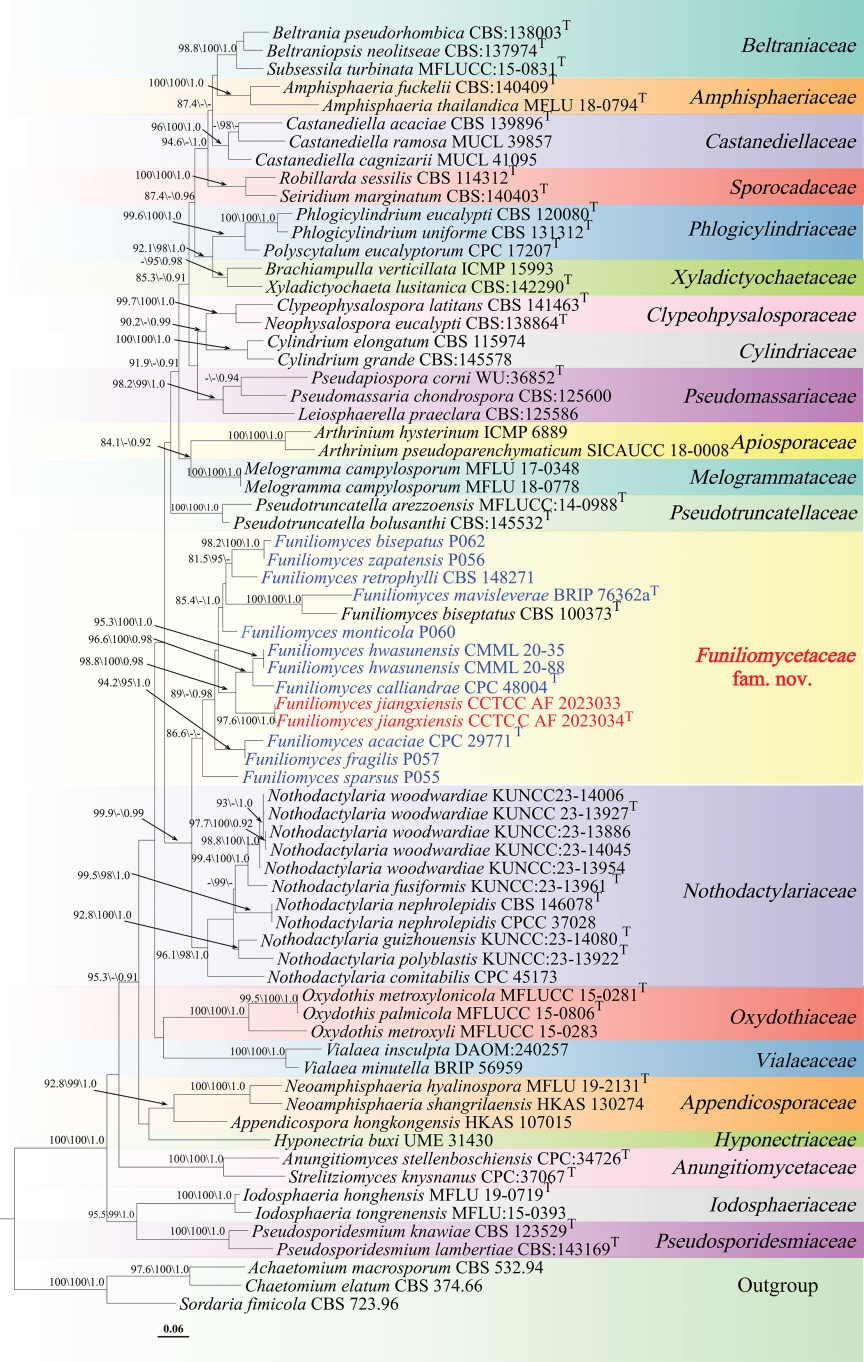
Maximum likelihood phylogenetic tree of *Amphisphaeriales* based on combined LSU, ITS, and RPB2 sequences, with Shimodaira-Hasegawa-like approximate likelihood ratio test (SH-aLRT) (left), ultrafast bootstrap (UFB) (middle), and Bayesian posterior probabilities (BPP) values (right) near the corresponding node. Only one of SH-aLRT > 80 or UFB > 95 for ML and BYPP > 0.90 for BI is indicated along the branches (SH-aLRT/UFB/BPP). Ex-type strains are marked with T after the strain number. The newly generated sequences are indicated in red and species for reclassification are in blue. The ML phylogram is available in TreeBASE (study accession S32462; http://purl.org/phylo/treebase/phylows/study/TB2:S32462).

The phylogenetic tree of *Amphisphaeriales* based on the three-gene dataset, rooted with *Achaetomium macrosporum* (CBS 532.94), *Chaetomium elatum* (CBS 374.66), and *Sordaria fimicola* (CBS 723.96), is shown in Fig. 2. The combined dataset of LSU, ITS, and RPB2 consisted of 2642 bp including gaps (LSU 857 bp, ITS 728 bp, and RPB2 1057 bp), which contained 1501 parsimony-informative characters, 247 singleton sites, and 1339 constant characters. The maximum likelihood matrix had 1804 distinct alignment patterns with 37.19% undetermined characters or gaps. The best maximum likelihood tree, with a final likelihood value of -29373.458, is shown in Fig. 2. The ML tree and BI analysis tree were similar, except for some interchanged positions and some unseparated clade. In this study, phylogenetic analyses revealed that our two new strains, together with ten previously described “*Dactylaria*” species and one *Funiliomyces* species, formed a distinct yet unplaced lineage within *Amphisphaeriales* (Fig. 2). The overall tree topology (Fig. 2) recovered in this study is largely congruent with those reported by Crous et al. (2022, 2025). Our phylogenetic analyses further confirmed that *Dactylaria* sensu lato is polyphyletic. *Dactylaria purpurella*, the type species of the genus, forms an independent lineage within *Pezizomycotina* and is distantly related to the *Amphisphaeriales* clade comprising ten “*Dactylaria*” taxa. (Fig. 1). Accordingly, these species in *Amphisphaeriales* are not congeneric with the type of *Dactylaria*. Based on phylogenetic evidence and following the principles of monophyly and the “One Fungus, One Name” concept, these ten species are here transferred to the genus *Funiliomyces*. Although *Funiliomyces biseptatus* is only known from its sexual morph, whereas the transferred species are asexual, phylogenetic placement indicates that they represent different morphs of the same genus. In addition, our analyses also showed that the two newly generated strains, CCTCC AF 2023033 and CCTCC AF 2023034, form a distinct branch and cluster as sister lineages with *Funiliomyces hwasunensis* (≡ *Dactylaria hwasunensis*) and *F. calliandrae* (≡ *D. calliandrae*), supported by SH-aLRT = 98.8%, UFB = 100%, and Bayesian posterior probability = 0.98 (Fig. 2), indicating that they represent a phylogenetically novel species in *Funiliomyces*.

Furthermore, the phylogenetic tree inferred from the combined three-locus dataset resolved twenty main clades within *Amphisphaeriales* (Fig. 2), corresponding to the families *Beltraniaceae* (98.8\100\1.0), *Amphisphaeriaceae* (100\100\1.0), *Castanediellaceae* (96\100\1.0), *Sporocadaceae* (100\100\1.0), *Phlogicylindriaceae* (99.6\100\1.0), *Xyladictyochaetaceae* (-\95\0.98), *Clypeophysalosporaceae* (99.7\100\1.0), *Cylindriaceae* (100\100\1.0), *Pseudomassariaceae* (98.2\99\1.0), *Apiosporaceae* (100\100\1.0), *Melogrammataceae* (100\100\1.0), *Pseudotruncatellaceae* (100\100\1.0), a distinct clade formed by *Funiliomyces* (86.6\-\-), *Nothodactylariaceae* (96.1\98\1.0), *Oxydothidaceae* (100\100\1.0), *Appendicosporaceae* (92.8\99\1.0), *Hyponectriaceae* (-/-/-), *Anungitiomycetaceae* (100\100\1.0), *Vialaeaceae* (100\100\1.0), *Pseudosporidesmiaceae* (100\100\1.0), and *Iodosphaeriaceae* (100\100\1.0). Both Bayesian inference and maximum likelihood analyses supported the monophyly of this *Funiliomyces* clade and its sister relationship to *Nothodactylariaceae* (SH-aLRT = 99.9%, UFB = 94%, BPP = 0.99; UFB < 95% is not shown on the tree). Based on these phylogenetic results, a new family, *Funiliomycetaceae* fam. nov., is proposed to accommodate the *Funiliomyces* lineage.

### Genetic distance analysis among families within *Amphisphaeriales*

To further clarify the phylogenetic position of the “*Dactylaria*” lineage within *Amphisphaeriales*, we conducted genetic divergence analyses based on ITS, LSU, and RPB2 sequences.

Within *Amphisphaeriales*, the ITS genetic distances among 19 families range from 0.110 (between *Sporocadaceae* and *Beltraniaceae*) to 0.369 (between *Pseudosporidesmiaceae* and *Iodosphaeriaceae*), indicating substantial inter-family variation in this locus (Table 3). When focusing on *Funiliomycetaceae*, its ITS genetic distances to the other 19 families range from 0.156 to 0.306, with the smallest distance to *Nothodactylariaceae* (0.156) and the largest to *Iodosphaeriaceae* (0.306). Notably, the minimum divergence of *Funiliomycetaceae* from other families (0.156) is still greater than the smallest inter-family distance within the order (0.110), suggesting that *Funiliomycetaceae* is not closely allied with any established family.

**Table 3.** Estimates of evolutionary divergence in ITS rDNA, LSU rDNA, and RPB2 sequences.

ITS rDNA																				
	* Beltraniaceae *	* Castanediellaceae *	* Clypeophysalosporaceae *	* Amphisphaeriaceae *	* Sporocadaceae *	* Pseudomassariaceae *	* Phlogicylindriaceae *	* Xyladictyochaetaceae *	* Cylindriaceae *	* Vialaeaceae *	* Nothodactylariaceae *	* Funiliomycetaceae *	* Pseudotruncatellaceae *	* Melogrammataceae *	* Appendicosporaceae *	* Apiosporaceae *	* Oxydothidaceae *	* Anungitiomycetaceae *	* Pseudosporidesmiaceae *	
* Castanediellaceae *	0.110																			
* Clypeophysalosporaceae *	0.181	0.168																		
* Amphisphaeriaceae *	0.145	0.157	0.217																	
* Sporocadaceae *	0.125	0.119	0.144	0.167																
* Pseudomassariaceae *	0.187	0.185	0.166	0.209	0.157															
* Phlogicylindriaceae *	0.150	0.145	0.185	0.178	0.145	0.186														
* Xyladictyochaetaceae *	0.137	0.122	0.189	0.162	0.128	0.173	0.136													
* Cylindriaceae *	0.140	0.132	0.130	0.150	0.152	0.150	0.136	0.121												
* Vialaeaceae *	0.240	0.239	0.228	0.255	0.225	0.213	0.238	0.259	0.242											
* Nothodactylariaceae *	0.250	0.233	0.237	0.270	0.223	0.183	0.223	0.244	0.195	0.238										
* Funiliomycetaceae *	0.230	0.229	0.230	0.264	0.217	0.182	0.223	0.229	0.209	0.231	0.156									
* Pseudotruncatellaceae *	0.178	0.154	0.147	0.179	0.130	0.158	0.181	0.159	0.167	0.212	0.206	0.219								
* Melogrammataceae *	0.227	0.214	0.209	0.255	0.159	0.179	0.204	0.202	0.180	0.240	0.213	0.221	0.145							
* Appendicosporaceae *	0.286	0.290	0.308	0.339	0.270	0.219	0.292	0.279	0.229	0.258	0.262	0.255	0.223	0.241						
* Apiosporaceae *	0.235	0.232	0.278	0.306	0.256	0.198	0.269	0.233	0.196	0.264	0.273	0.272	0.204	0.207	0.253					
* Oxydothidaceae *	0.288	0.272	0.298	0.324	0.233	0.253	0.281	0.284	0.214	0.262	0.297	0.299	0.221	0.275	0.281	0.282				
* Anungitiomycetaceae *	0.310	0.282	0.293	0.339	0.276	0.233	0.293	0.298	0.245	0.282	0.286	0.280	0.218	0.225	0.300	0.302	0.297			
* Pseudosporidesmiaceae *	0.316	0.314	0.308	0.335	0.302	0.265	0.292	0.321	0.280	0.263	0.257	0.258	0.298	0.293	0.297	0.313	0.318	0.269		
* Iodosphaeriaceae *	0.356	0.329	0.363	0.369	0.348	0.275	0.333	0.318	0.298	0.270	0.321	0.306	0.252	0.271	0.341	0.330	0.320	0.316	0.258	
LSU rDNA																				
	* Beltraniaceae *	* Amphisphaeriaceae *	* Castanediellaceae *	* Phlogicylindriaceae *	* Xyladictyochaetaceae *	* Funiliomycetaceae *	* Cylindriaceae *	* Nothodactylariaceae *	* Pseudotruncatellaceae *	* Oxydothidaceae *	* Anungitiomycetaceae *	* Hyponectriaceae *	* Sporocadaceae *	* Clypeophysalosporaceae *	* Melogrammataceae *	* Apiosporaceae *	* Pseudomassariaceae *	* Appendicosporaceae *	* Pseudosporidesmiaceae *	* Iodosphaeriaceae *
* Amphisphaeriaceae *	0.047																			
* Castanediellaceae *	0.041	0.058																		
* Phlogicylindriaceae *	0.055	0.056	0.050																	
* Xyladictyochaetaceae *	0.049	0.055	0.040	0.032																
* Funiliomycetaceae *	0.059	0.061	0.044	0.050	0.042															
* Cylindriaceae *	0.057	0.053	0.048	0.051	0.044	0.053														
* Nothodactylariaceae *	0.064	0.067	0.055	0.052	0.040	0.040	0.047													
* Pseudotruncatellaceae *	0.060	0.062	0.051	0.053	0.039	0.036	0.053	0.038												
* Oxydothidaceae *	0.056	0.051	0.051	0.051	0.044	0.038	0.053	0.047	0.037											
* Anungitiomycetaceae *	0.072	0.069	0.070	0.076	0.064	0.057	0.059	0.065	0.053	0.052										
* Hyponectriaceae *	0.077	0.080	0.076	0.076	0.065	0.058	0.059	0.054	0.045	0.058	0.059									
* Sporocadaceae *	0.061	0.062	0.062	0.053	0.058	0.066	0.058	0.056	0.070	0.060	0.080	0.083								
* Clypeophysalosporaceae *	0.062	0.054	0.063	0.070	0.063	0.069	0.050	0.062	0.064	0.058	0.072	0.071	0.065							
* Melogrammataceae *	0.060	0.056	0.059	0.047	0.037	0.044	0.046	0.044	0.045	0.036	0.054	0.058	0.055	0.053						
* Apiosporaceae *	0.065	0.064	0.063	0.062	0.062	0.056	0.056	0.057	0.052	0.049	0.060	0.069	0.070	0.065	0.047					
* Pseudomassariaceae *	0.067	0.068	0.064	0.071	0.063	0.065	0.063	0.065	0.065	0.062	0.080	0.076	0.073	0.068	0.065	0.073				
* Appendicosporaceae *	0.076	0.077	0.083	0.083	0.078	0.065	0.070	0.069	0.063	0.057	0.056	0.057	0.081	0.077	0.060	0.066	0.082			
* Pseudosporidesmiaceae *	0.088	0.093	0.098	0.097	0.086	0.078	0.092	0.086	0.077	0.066	0.066	0.080	0.100	0.092	0.073	0.079	0.102	0.053		
* Iodosphaeriaceae *	0.087	0.091	0.098	0.102	0.096	0.084	0.104	0.100	0.087	0.073	0.074	0.089	0.110	0.098	0.084	0.089	0.106	0.067	0.051	
* Vialaeaceae *	0.076	0.075	0.066	0.073	0.067	0.057	0.083	0.072	0.061	0.058	0.074	0.077	0.084	0.086	0.062	0.070	0.085	0.069	0.081	0.089
RPB2 rDNA																				
	* Beltraniaceae *	* Melogrammataceae *	* Cylindriaceae *	* Sporocadaceae *	* Nothodactylariaceae *	* Funiliomycetaceae *	* Amphisphaeriaceae *	* Phlogicylindriaceae *	* Apiosporaceae *	* Iodosphaeriaceae *	* Appendicosporaceae *	* Oxydothidaceae *								
* Melogrammataceae *	0.208																			
* Cylindriaceae *	0.251	0.215																		
* Sporocadaceae *	0.234	0.241	0.286																	
* Nothodactylariaceae *	0.273	0.256	0.293	0.285																
* Funiliomycetaceae *	0.252	0.259	0.270	0.309	0.262															
* Amphisphaeriaceae *	0.259	0.247	0.285	0.256	0.303	0.304														
* Phlogicylindriaceae *	0.262	0.229	0.271	0.296	0.296	0.309	0.313													
* Apiosporaceae *	0.280	0.286	0.280	0.299	0.298	0.298	0.296	0.301												
* Iodosphaeriaceae *	0.238	0.262	0.319	0.308	0.307	0.311	0.311	0.293	0.340											
* Appendicosporaceae *	0.276	0.282	0.344	0.312	0.315	0.316	0.349	0.323	0.353	0.285										
* Oxydothidaceae *	0.326	0.305	0.315	0.315	0.338	0.324	0.367	0.298	0.342	0.349	0.375									
* Anungitiomycetaceae *	0.376	0.392	0.420	0.418	0.392	0.426	0.433	0.399	0.440	0.366	0.397	0.446								

Notes: The number of base substitutions per site between family is shown.

For the LSU dataset, the genetic distances among the 20 families range from 0.032 between *Xyladictyochaetaceae* and *Phlogicylindriaceae* to 0.110 between *Sporocadaceae* and *Iodosphaeriaceae*. The LSU distances between *Funiliomycetaceae* and the other families vary from 0.036 with *Pseudotruncatellaceae* to 0.084 with *Iodosphaeriaceae*. Again, the smallest LSU distance between *Funiliomycetaceae* and *Pseudotruncatellaceae* (0.036) remains higher than the overall minimum value (0.032) observed within the order, supporting its independent molecular identity.

For the RPB2 dataset, only 13 families were included because not all families within *Amphisphaeriales* have publicly available RPB2 sequences. Nevertheless, the inter-family distances reveal clear divergence of *Funiliomycetaceae*. Overall, the inter-family distances range from 0.208 between *Melogrammataceae* and *Beltraniaceae* to 0.446 between *Anungitiomycetaceae* and *Oxydothidaceae*. The RPB2 distances between *Funiliomycetaceae* and the other families range from 0.252 with *Beltraniaceae* to 0.426 with *Anungitiomycetaceae*. The minimum distance between *Funiliomycetaceae* and *Beltraniaceae* (0.252) is notably higher than the smallest inter-family distance in the dataset (0.208), indicating consistent divergence.

Collectively, the analyses based on ITS, LSU, and RPB2 sequences demonstrate that *Funiliomycetaceae* is genetically distinct from all other families within *Amphisphaeriales*. Across three loci, its inter-family distances are consistently greater than the smallest distances observed among other established families, providing robust molecular evidence that *Funiliomycetaceae* represents an independent family within the order.

### Taxonomy

#### Funiliomycetaceae

Taxon classification

Animalia

AmphisphaerialesFuniliomycetaceae

L.X. Mi, K.D. Hyde, H.Y. Song & D.M. Hu fam. nov.

1F5906FE-B7AA-56E9-BDE3-BAAB5CA80D61

Index Fungorum: IF904529

Facesoffungi Number: FoF18896

##### Etymology.

Name refers to the type genus *Funiliomyces*.

##### Type genus.

*Funiliomyces* Aptroot, Studies in Mycology 50(2): 309 (2004).

##### Description.

Saprobic, endophytic, or epiphytic on diverse plant hosts in tropical to temperate regions. Sexual morph: ***Ascomata*** black, subglobose, immersed to erumpent. ***Wall*** composed of irregular layers of regularly melanized, flattened cells, with no color change in KOH. ***Paraphyses*** absent. ***Asci*** cylindrical, 8-spored, with a thickened apex bearing a central refractive, IKI-negative apical apparatus, and enclosed by parenchymatous tissue. ***Ascospores*** pale brown, torpedo-shaped, 2-septate, upper cell pointed, lower cell rounded, hyaline granules or oil droplets, bearing two hyaline mucilaginous appendages. Asexual morph: ***Mycelium*** consisting of branched, septate, smooth, hyaline to pale brown hyphae, *s*ometimes forming hyphal ropes. ***Conidiophores*** macronematous, mononematous, solitary or in small groups, erect, straight to flexuous, subcylindrical to cylindrical (apex sometimes inflated), simple or occasionally branched, hyaline to brown, septate, sometimes reduced to conidiogenous cells. ***Conidiogenous cells*** integrated, terminal or lateral, sympodial, mono- to polyblastic, hyaline to brown, cylindrical to clavate, with flat-tipped or denticulate apices; denticles (when present) large, cylindrical to geniculate, cylindrical, truncate, or pimple-like, or lacking entirely. ***Conidia*** solitary, hyaline to pale smoky, smooth, septate, narrowly fusiform to cylindrical, with obtuse, subobtuse, or tapering apices and truncate or rounded bases; dimensions variable among species.

##### Notes.

A new family, *Funiliomycetaceae*, is proposed to accommodate a distinct, strongly supported clade comprising the genus *Funiliomyces* and several related lineages historically identified as “*Dactylaria*”. *Funiliomycetaceae* shares general sexual characters with *Amphisphaeriales*, including immersed to semi-immersed ascomata and unitunicate asci with an apical ring (Aptroot 2004). However, the family is readily distinguished from all currently accepted families in the order by its unique ascospore morphology: the ascospores are fusiform, consistently biseptate, and bear both apical and median appendages, a combination of characters not reported in other families of *Amphisphaeriales* (Aptroot 2004). The asexual morph of *Funiliomycetaceae* is hyphomycetous, characterized by conidiogenous cells that are sympodial, terminal or lateral, integrated, and denticulate (when present, denticles are large and cylindrical to geniculate), producing solitary, hyaline to pale smoky, smooth, septate, narrowly fusiform to cylindrical conidia with obtuse to tapering apices and truncate or rounded bases. *Funiliomycetaceae* shares similar sexual features with *Nothodactylariaceae (Nothodactylaria)*, such as *Nothodactylaria nephrolepidis* (Cours et al. 2019b). However, *Funiliomycetaceae* is phylogenetically distinct from the families in *Amphisphaeriales*, as supported by multi-locus phylogenetic analyses (Fig. 2) and inter-familial genetic distance comparisons (Table 3).

#### Funiliomyces

Taxon classification

Animalia

AmphisphaerialesFuniliomycetaceae

Aptroot, Stud. Mycol. 50 (2): 309 (2004)

8987BC25-26FA-557A-A755-8B01ACE7817F

Index Fungorum: IF500077

Facesoffungi Number: FoF18897

##### Type species.

*Funiliomyces biseptatus* Aptroot, Stud. Mycol. 50 (2): 309 (2004). Index Fungorum: IF500164.

##### Holotype.

CBS H-10505.

##### Type information.

Brazil, Minas Gerais, Catas Altas, Serro do Caraça, Parque Natural do Caraça, near Funil, 1 km NW of monastery Santuário do Caraça, 20°06'S, 43°29'W, on dead leaf of *Bromeliaceae* in rock field, 18 Sept. 1997, A. Aptroot, holotype herb. CBS H-10505, isotypes herb. SP, living culture ex-type CBS 100373, also dried culture CBS H-10506.

##### Description.

See Aptroot (2004) on page 309.

##### Emended diagnosis.

Saprobic, endophytic, or epiphytic on diverse plant hosts in tropical to temperate regions. Sexual morph: ***Ascomata*** black, subglobose, immersed to erumpent. ***Wall*** composed of irregular layers of regularly melanized, flattened cells, with no color change in KOH. ***Paraphyses*** absent. ***Asci*** cylindrical, 8-spored, with a thickened apex bearing a central refractive, IKI-negative apical apparatus, and enclosed by parenchymatous tissue. ***Ascospores*** pale brown, torpedo-shaped, 2-septate, upper cell pointed, lower cell rounded, hyaline granules or oil droplets, bearing two hyaline mucilaginous appendages. Asexual morph: ***Mycelium*** consisting of branched, septate, smooth, hyaline to pale brown hyphae, *s*ometimes forming hyphal ropes. ***Conidiophores*** macronematous, mononematous, solitary or in small groups, erect, straight to flexuous, subcylindrical to cylindrical (apex sometimes inflated), simple or occasionally branched, hyaline to brown, septate, sometimes reduced to conidiogenous cells. ***Conidiogenous cells*** integrated, terminal or lateral, sympodial, mono- to polyblastic, hyaline to brown, cylindrical to clavate, with flat-tipped or denticulate apices; denticles (when present) large, cylindrical to geniculate, cylindrical, truncate, or pimple-like, or lacking entirely. ***Conidia*** solitary, hyaline to pale smoky, smooth, septate, narrowly fusiform to cylindrical, with obtuse, subobtuse, or tapering apices and truncate or rounded bases, dimensions variable among species.

##### Notes.

*Funiliomyces* was first described by Aptroot (2004) as a monospecific genus, containing only its type species *F. biseptatus*s. With respect to ten species previously assigned to *Dactylaria*, our phylogenetic evidence shows they are phylogenetically distant from the type species of the genus (*Dactylaria purpurella*) (Fig. 1). However, these ten species cluster with *Funiliomyces biseptatus*, forming a distinct, well-supported lineage (Fig. 2). Therefore, in accordance with the “One Fungus, One Name” principle, we transfer these ten species to *Funiliomyces* and introduce the new species *F. jiangxiensis* within this genus. This emendation expands the genus to encompass both sexual and asexual morphs. The type species, *F. biseptatus*, represents the sexual morph, characterized by torpedo-shaped ascospores with two nearly central septa and appendages. In contrast, the eleven other species represent the asexual morphs, producing hyaline, septate conidiophores with sympodial, denticulate conidiogenous cells and solitary, hyaline, clavate or fusoid-ellipsoid conidia.

#### Funiliomyces jiangxiensis

Taxon classification

Animalia

AmphisphaerialesFuniliomycetaceae

L.X. Mi, K.D. Hyde, H.Y. Song & D.M. Hu sp. nov

C4E7F4E0-6D94-56B2-B84C-E02A364C46B2

Index Fungorum: IF904530

Facesoffungi Number: FoF18898

Fig. 3

##### Etymology.

The name refers to the place where the fungal was collected.

**Figure 3.**
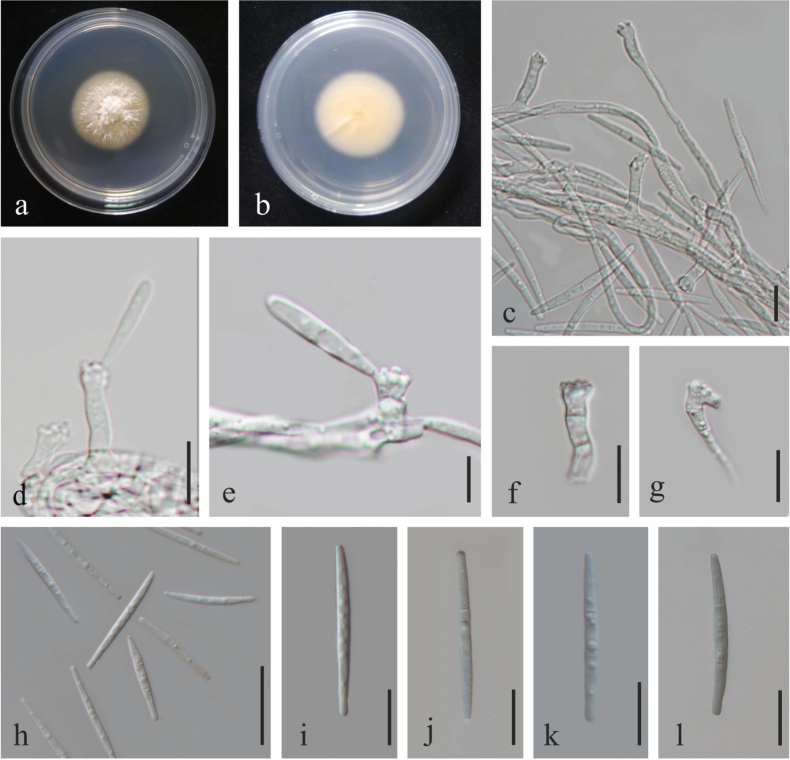
*Funiliomyces jiangxiensis* sp. nov. (HFJAU10125, holotype) **a, b** Colonies on the front and back of PDA medium (for 5 days); **c** Hyphae and sporulation structures; **d, e** Conidiophores bearing a conidium initial on one of its denticles; **f, g** Conidiophores; **h–l** Conidia. Scale bars: 10 μm (**c, d**); 5 μm (**e**); 10 μm (**f, g**); 20 μm (**h**); 10 μm (**i–l**).

##### Holotype.

HFJAU10125.

##### Description.

Endophytic fungus isolated from the roots of *Tetradium ruticarpum*. Sexual morph: Undetermined. Asexual morph: ***Mycelium*** consisted of hyaline, smooth, branched, septate, 1.7–2.6 µm diam hyphae. ***Conidiophores*** (6–)8.5–17.5(–23.5) × 1.8–3.5(–4) µm (mean ± SD = 12.5 ± 4.3 × 3 ± 0.4 µm, n = 35), macronematous, mononematous, hyaline, subcylindrical, arising from terminal or intercalary parts of aerial hyphae, mostly reduced to conidiogenous cells (rarely with a supporting cell), tapering towards the base, apex polyblastic, sympodial, inflated or geniculous-sinuous, with conspicuous, cylindrical denticles, up to 0.9 µm wide. ***Conidia*** 20–40 × 1.5–3.5 μm (mean ± SD = 27.8 ± 3.9 × 2.6 ± 0.3 µm, n = 40), hyaline, narrowly fusoid-ellipsoid, 0–3-septate, guttulate, apex sub-obtuse, base truncate.

##### Culture characteristics.

Colonies on PDA at 25 °C for 5 days, convex, white with cream margin, reverse pale brown with cream margin, no pigment in agar.

##### Material examined.

CHINA, Jiangxi Province, Ganzhou City, Ganxian District, 25.6121°N, 115.1211°E 412.9 m asl, isolated from healthy roots of *Tetradium ruticarpum*, 26 June 2022, Lixue Mi, dry culture HFJAU10125 (holotype); ex-type JAUCC 5298 = CCTCC AF 2023034; *ibid*., Yichun City, Zhangshu county-level city, 27.9931°N, 115.2123°E, 46 m asl, 21 September 2021, Lixue Mi, dry culture HFJAU10124; living culture: JAUCC 4255 = CCTCC AF 2023033.

##### Notes.

In the multi-gene analysis, *Funiliomyces jiangxiensis* (strains CCTCC AF 2023033 and CCTCC AF 2023034) forms a distinct lineage that groups with *F. hwasunensis* (≡ *Dactylaria hwasunensis*) and *F. calliandrae* (≡ *D. calliandrae*) as a sister branch, supported by high statistical values in the phylogenetic tree (SH-aLRT/UFB/BPP = 98.8/100/0.98). Morphologically, *Funiliomyces jiangxiensis* can be distinguished from *F. hwasunensis* by their conidiogenous cells and conidia. Specifically, *Funiliomyces jiangxiensis* develops distinct denticles on its conidiogenous cells, a feature that is absent in *F. hwasunensis*. Additionally, there is a notable difference in the number of septa in their conidia: *Funiliomyces jiangxiensis* has conidia with 0–3 septa, whereas *F. hwasunensis* produces conidia with more septa, ranging from 1 to 5. In terms of morphology, *F. jiangxiensis* resembles *F. calliandrae* but can be distinguished from it. Both have denticles on conidiogenous cells, yet those of *F. jiangxiensis* (0.9 μm) are shorter than *F. calliandrae*’s (1–3 μm). Additionally, the conidia of *F. jiangxiensis* (20–40 μm) are shorter than those of *F. calliandrae* [(37–)40–45(–47) μm]. The species was isolated from the roots of *Tetradium ruticarpum* (medicinal plant), which provides a basis for further research on its potential functional properties.

### Other species in *Funiliomyces*

#### Funiliomyces acaciae

Taxon classification

Animalia

AmphisphaerialesFuniliomycetaceae

(Crous) L.X. Mi, H.Y. Song, D.M. Hu & K.D. Hyde comb. nov.

F81157E9-1057-56E8-A44A-9566BD4A5A14

Index Fungorum: IF819073

##### Basionym.

*Dactylaria acaciae* Crous, Persoonia 37: 321 (2016).

##### Holotype.

CBS H-22876.

##### Type information.

USA, Hawaii, Oahu, on leaves of *Acacia koa* (*Fabaceae*), 30 September 2015, J.J. Le Roux (holotype CBS H-22876, culture ex-type CPC 29771 = CBS 142087).

##### Description.

See the original description in D’Souza et al. (2002) on page 141.

#### Funiliomyces bisepatus

Taxon classification

Animalia

AmphisphaerialesFuniliomycetaceae

(Matsushima) L.X. Mi, H.Y. Song, D.M. Hu & K.D. Hyde comb. nov.

35065E27-80F5-5BFF-A470-B6A5F948E900

Index Fungorum: IF312614

##### Basionym.

*Dactylaria biseptata* Matsushima, Icones Microfungorum a Matsushima lectorum: 48 (1975).

##### Holotype.

MFC-4029.

##### Type information.

Japan, Ohdaigahara, Nara Pref, on a rotten leaf of *Rhododendron metternichii* (*Ericaceae*), July 1970, MFC-4029 (holotype).

##### Description.

See the original description in Matsushima (1975) on page 48–49.

#### Funiliomyces calliandrae

Taxon classification

Animalia

AmphisphaerialesFuniliomycetaceae

(Crous) L.X. Mi, H.Y. Song, D.M. Hu & K.D. Hyde comb. nov.

49F7F1F4-0A9A-57DD-8C14-DADE48E5762E

Index Fungorum: IF859210

##### Basionym.

*Dactylaria calliandrae* Crous et al. Persoonia 54: 376–377 (2025).

##### Holotype.

CBS H-25715.

##### Type information.

Brazil, Minas Gerais, Viçosa, Clonar nursery, on living leaf of *Calliandra tweediei* (*Fabaceae*), 25 February, 2024, P.W. Crous, HPC 4399 (holotype CBS H-25715; culture ex-type COAD 3994 = CPC 48004).

##### Description.

See the original description in Crous et al. (2025) on page 376–377.

#### Funiliomyces fragilis

Taxon classification

Animalia

AmphisphaerialesFuniliomycetaceae

(de Hoog) L.X. Mi, H.Y. Song, D.M. Hu & K.D. Hyde comb. nov.

BEA3A81B-7FCA-508A-BFFB-06B2C5C603EE

Index Fungorum: IF104169

##### Basionym.

*Dactylaria fragilis* de Hoog, Studies in Mycology 26: 30 (1985).

##### Holotype.

No.6074(CBS).

##### Type information.

The Netherlands, Opsterland, Oldeterp, on cupules of *Fagus sylvatica* (*Fagaceae*), H.A. van der Aa, October, 1977.

##### Description.

See the original description in de Hoog and van Oorscho (1985) on page 30.

#### Funiliomyces hwasunensis

Taxon classification

Animalia

AmphisphaerialesFuniliomycetaceae

(H.F. Liu & H.K. Sang) L.X. Mi, H.Y. Song, D.M. Hu & K.D. Hyde comb. nov.

0E0930FD-B913-5AEF-BDA3-199EF4DB63F0

Index Fungorum: IF857258

##### Basionym.

*Dactylaria hwasunensis* H.F. Liu & H.K, IMA Fungus 16(e138479): 10 (2025).

##### Holotype.

CMML 20-35H.

##### Type information.

Korea, South Jeolla Province, Hwasun, isolated from roots of *Zoysia japonica* (*Poaceae*), October 2020, H. Liu & H. Sang, holotype CMML 20-35H, ex-holotype CMML 20-35, ex-isotype CMML 20-88.

##### Description.

See the original description in Liu et al. (2025) on page 12–14.

#### Funiliomyces mavisleverae

Taxon classification

Animalia

AmphisphaerialesFuniliomycetaceae

(Y.P. Tan, Bishop-Hurley & Marney) L.X. Mi, H.Y. Song, D.M. Hu & K.D. Hyde comb. nov.

C0E4BDBC-B516-562F-AB90-5A208DABF199

Index Fungorum: IF902836

##### Basionym.

*Dactylaria mavisleverae* Y.P. Tan, Bishop-Hurley & Marney, Index of Australian Fungi 46: 3 (2024).

##### Holotype.

BRIP 76362a.

##### Type information.

Australia, Queensland, Brisbane, phylloplane of unidentified ornamental plant, January, 2024, T.S. Marney, BRIP 76362a (holotype).

##### Description.

See the original description in Tan et al. (2024) on page 3–4.

#### Funiliomyces monticola

Taxon classification

Animalia

AmphisphaerialesFuniliomycetaceae

(R. F. Castañeda & W. B. Kendr.) L.X. Mi, H.Y. Song, D.M. Hu & K.D. Hyde comb. nov.

7E02CE3E-BF51-5EC0-A89D-F02181465D82

Index Fungorum: IF361523

##### Basionym.

*Dactylaria monticola* R.F. Castañeda & W.B. Kendr, University of Waterloo Biology Series, 35: 30 (1991).

##### Holotype.

INIFAT C 91/82.

##### Type information.

Cuba, Granma, Buey Arriba, La Estrella, on dead leaves of *Andira inermis* (*Leguminosae*), R.F. Castañeda, 14 March 1991.

##### Description.

See the original description in Castañeda and Kendr (1991) on page 30.

#### Funiliomyces retrophylli

Taxon classification

Animalia

AmphisphaerialesFuniliomycetaceae

(Crous) L.X. Mi, H.Y. Song, D.M. Hu & K.D. Hyde comb. nov.

D277145E-E22D-5B20-8EEC-CAA7314CC75B

Index Fungorum: IF844283

##### Basionym.

*Dactylaria retrophylli* Crous, Fungal Systematics and Evolution 10: 41 (2022).

##### Holotype.

HPC 3260.

##### Type information.

Colombia, Finca El Cedral, on leaves of *Retrophyllum rospigliosii* (*Podocarpaceae*), M.J. Wingfield, February, 2020, HPC 3260 (holotype CBS H-24817, culture ex-type CPC 39510 = CBS 148271).

##### Description.

See the original description in Crous et al. (2022) on page 41–42.

#### Funiliomyces sparsus

Taxon classification

Animalia

AmphisphaerialesFuniliomycetaceae

(R. F. Castañeda & W. B. Kendr.) L.X. Mi, H.Y. Song, D.M. Hu & K.D. Hyde comb. nov.

0F863F0C-362F-5604-BC23-01C7C748F899

Index Fungorum: IF361528

##### Basionym.

*Dactylaria sparsa* R.F. Castañeda & W.B. Kendr, University of Waterloo Biology Series. 35:33 (1991).

##### Holotype.

INIFAT C 91/68-2.

##### Type information.

Cuba, C. Habana, Santiago de las Vegas, on decaying leaves, R.F. Castañeda, 18 February 1990.

##### Description.

See the original description in Castañeda and Kendr (1991) on page 33.

#### Funiliomyces zapatensis

Taxon classification

Animalia

AmphisphaerialesFuniliomycetaceae

(R.F. Castañeda) L.X. Mi, H.Y. Song, D.M. Hu & K.D. Hyde comb. nov.

8E847D83-3E72-582C-818E-5CFA62B73CD2

Index Fungorum: IF125340

##### Basionym.

*Dactylaria zapatensis* R.F. Castañeda, Fungi Cubenses III (La Habana): 5 (1988)

##### Holotype.

INIFAT C85/98

##### Type information.

Cuba, Matanzas, Ciénaga de Zapata, on fallen leaves of *Nectandra coriacea* (*Lauraceae*), R.F. Castañeda Ruiz, 26 May 1985.

##### Description.

See the original description in Castañeda Ruiz (1988) on page 5.

*Funiliomyces* currently includes only a single sexual morph species, *F. biseptatus*; therefore, we summarized the asexual morphological features, lifestyle, host associations, and distribution for all species (Table 4) to facilitate comparison within the genus.

**Table 4.** Asexual morphological features, lifestyle, host associations, and distribution of *Funiliomyces* species.

**Species**	**Mycelium**	**Conidiophores**	**Conidiogenous cel**ls	**Conidi**a		**Lifestyle**	**Host**	**Country**	**References**
				**Shape/colour**	**Size**				
*Funiliomyces acaciae*	2–2.5 µm, hyaline,	7–60 × 2–3.5 µm, brown, 0–7-septate	7–25 × 2–3.5 µm, brown, with flat-tipped denticles (0.5–1.5 × 0.5 µm)	narrowly fusoid ellipsoid, 2-septate, hyaline	(16–)25– 34(–37) × 2(–2.5) µm	epiphytic	*Acacia koa* (*Fabaceae*)	USA	Crous et al. (2016)
*F. biseptatus*	1–3 µm, hyaline to moderately brown	5–20 µm × 3–3.5 µm, moderately brown, cylindrical, 0–2-septata	cylindrical, moderately brown, with successive denticles and geniculate structure	cylindrical, 2-septata, individually, hyaline to pale smoky	(22–) 27–33 (–35) × 1.5–2 µm	saprobic	*Rhododendron metternichii* (*Ericaceae*)	Japan	Matsushima (1975)
*F. biseptatus*	–	–	–	–	–	saprobic	Undefined (*Bromeliaceae*)	Brazil	Aptroot (2004)
*F. calliandrae*	2–3 µm, hyphae	hyaline (appearing subhyaline with age), mostly reduced to conidiogenous cells	10–25 × 3–4 µm hyaline, prominent cylindrical denticles, 1–3 × 1.5 µm	spindle-shaped, apex subobtuse, base truncate, (3–)5–6(–8)-septate, hyaline	(37–)40–45(–47) × (2.5–)3 µm	epiphytic	*Calliandra tweediei* (*Fabaceae*)	Brazil	Crous et al. (2025)
*F. fragilis*	pale brown	15–30 × 4 μm at the base, 0–3 thin septa, subhyaline to pale brown	thin-walled, hyaline, slightly lobed; denticles absent; rhexolytic secession with inconspicuous scars	clavate, 2-septate, hyaline	18–26 × 1.5, base 0.6 µm wide	saprobic	*Fagus sylvatica* (*Fagaceae*)	Netherlands	de Hoog and van Oorscho (1985)
*F. hwasunensis*	–	6–35 × 2.2–2.8 µm， hyaline, aseptate or septate	2–2.8 μm wide，terminal, integrated, hyaline	clavate, blunt end，hyaline, 1–5 septate	10–60 × 2.2–2.8 μm	endophytic	*Zoysia japonica* (*Poaceae*)	Korea	Liu et al. (2025)
*F. jiangxiensis*	1.7–2.6 µm, hyaline	5–37 × 2–4 µm, hyaline, septate, sometimes reduced to conidiogenous cells	4–18 × 1–4 µm, with conspicuous, cylindrical denticles, up to 0.9 µm wide	narrowly fusoid-ellipsoid, guttulate, 0–3-septate, hyaline	20–40 × 1.5–4 µm	endophytic	*Tetradium ruticarpum* (*Rutaceae*)	China	This study
*F. mavisleverae*	–	–	–	–	–	*epiphytic*	an unidentified ornamental plant	Australia	Tan and Shivas (2024)
*F. monticola*	1–1.5 μm, colorless	15–40 × 2–2.5 μm, colorless, septate	12–17 × 2–3 µm, with conspicuous, truncate denticles in the apical region	fusiform, 1-septate, colourless	30–37 × 1–1.5 µm	saprobic	*Andira inermis* (*Leguminosae*)	Cuba	Castañeda and Kendr (1991)
*F. retrophylli*	1.5–2.5 μm, hyaline	conidiophores reduced to conidiogenous cells or with supporting cell	with 1–2 × 1 μm apex denticulate	medianly 1-septate, straight to narrowly fusoid, 6–20 × 2.5–3.5	(26–)30–33(–37) × (1.5–)2 µm	epiphytic	*Retrophyllum rospigliosii* (*Podocarpaceae*)	Colombia	Crous et al. (2022)
*F. sparsus*	1–1.5 μm, pale brown	0–1-septate, pale brown usually reduced to a conidiogenous cell.	7–12 × 3–4 µm, pale brown or almost colorless, with large, conspicuous, truncate denticles, 1.5–2 μm	subcylindrical, (2–)3-septated with a false septum near each end, colorless or almost colorless	26–36 × 1.5–2 µm	saprobic	decaying leaves (unidentified)	Cuba	Castañeda and Kendr (1991)
*F. zapatensis*	1–1.5 μm, light brown	12–60 × 1–2 µm, septate, light brown, up to 24 µm wide at apex	polyblastic, denticulate, sympodial, inflated at apex	cylindrical, 2-septata, hyaline, septa visible near extremities	18–26 × 1–1.5 µm	saprobic	*Nectandra coriacea* (*Lauraceae*)	Cuba	Castañeda Ruiz (1988)

Notes: The symbol “–” denotes no information available.

## Discussion

Previous studies have consistently demonstrated that *Dactylaria* is polyphyletic, yet the taxonomic placement of its type species, *D. purpurella*, has never been formally resolved. To date, there has been no phylogenetic study specifically designed to address the classification of *D. purpurella*; rather, it has generally been included only as part of broader datasets, without being treated as a focal taxon. As a result, its placement has remained ambiguous. For instance, Bussaban et al. (2005) showed that *D. purpurella* clustered with *Ochroconis* rather than other *Dactylaria* species based on ITS sequence data. Similarly, Réblová (2009) using LSU sequence data, demonstrated that *D. purpurella*, *D. monticola*, and *D. parvispora* fall into distinct clades, with *D. purpurella* forming an isolated lineage, while *D. monticola* grouping within *Xylariales*, and *D. parvispora* clustering with *Papulosa amerospora* in *Papulosaceae*. Subsequent studies further added to this complexity, placing *D. purpurella* within *Magnaporthales* (Klaubauf et al. 2014) or *Venturiales* (Lin et al. 2021). Taken together, these conflicting results highlight not only the unstable phylogenetic position of *D. purpurella*, but also the broader taxonomic challenges in delimiting generic boundaries within *Dactylaria*. In our study, the LSU-based phylogenetic analysis of *Pezizomycotina* demonstrated that *Dactylaria* is polyphyletic, with its species distributed across multiple classes, predominantly within *Sordariomycetes*. However, the phylogenetic placement of four species, *D. purpurella* (the type species), *D. humicola*, *D. dimorpha*, and *D. dimorphospora*, has not been assigned to any of the known classes within *Pezizomycotina* and thus remains unresolved (Fig. 1). As the first systematic investigation of the phylogenetic position of *Dactylaria*, based on all available LSU sequences data, our work provides a comprehensive overview of its taxonomic placement. The results emphasize the necessity of additional sampling and multi-locus phylogenetic analyses to resolve the taxonomy and evolutionary relationships within the genus, thereby laying the foundation for future refinement of its classification.

Although *Dactylaria* is polyphyletic, new species have continued to be introduced into the genus in recent years (Crous et al. 2016, 2022, 2025), all of which are placed within *Amphisphaeriales*, forming a single unresolved lineage. Fortunately, we also collected two new strains that cluster with the recently published *Dactylaria* species. Phylogenetic analysis based on the combined LSU, ITS, and RPB2 loci reveals that ten previously described “*Dactylaria*” species, our newly obtained isolates, and *Funiliomyces biseptatus* (type strain CBS 100373) cluster together into a single clade within *Amphisphaeriales* (Fig. 2). Topologically, this clade is comparable to lineages recovered in multiple previous studies, which remained unplaced within any established family of *Amphisphaeriales* (Crous et al. 2016, 2022, 2025). With the discovery of additional species of *Nothodactylariaceae* (Zhang et al. 2025) and their incorporation into the phylogenetic analyses of this study, this well-distinguished clade is recovered as a sister lineage to *Nothodactylaria*, the sole genus currently recognized in *Nothodactylariaceae*, with strong phylogenetic support (SH-aLRT/UFB/BPP = 99.9/94/0.99). In addition, multi-locus genetic distance analyses (ITS, LSU, and RPB2) revealed substantial divergence between this clade and other families within *Amphisphaeriales*, further supporting its independence from previously recognized families. Based on our results (Fig. 2, Table 3), both the phylogenetic analyses and genetic distance divergence clearly support that this distinct lineage represents a family-level taxon within *Amphisphaeriales*. Therefore, we establish the new family *Funiliomycetaceae* within *Amphisphaeriales* to accommodate this distinct lineage.

Morphologically, *Funiliomycetaceae* integrates both sexual and asexual morphs. *Funiliomyces biseptatus*, the only species with a sexual morph, produces torpedo-shaped ascospores with two nearly central septa and appendages, while the eleven other species represent asexual morphs, producing hyaline, septate conidiophores with sympodial, denticulate conidiogenous cells and solitary, hyaline, clavate or fusoid-ellipsoid conidia. This integration of sexual and asexual morphs within a single lineage highlights the evolutionary significance of the family and provides a robust morphological framework for family-level delimitation. When compared with *Nothodactylariaceae*, morphological evidence corroborates the close relationship between the two families, yet the overall conidial range in *Funiliomycetaceae* is considerably broader, encompassing species with distinctly larger conidia (Table 4). A more definitive diagnostic feature is the diversity of conidiogenous cells, which bear large cylindrical to geniculate denticles or lack denticles entirely, in sharp contrast to the uniform, pimple-like denticles of *Nothodactylariaceae* (Crous et al. 2019b, Zhang et al. 2025). Future studies will require additional species sampling and isolation to uncover sexual morphs, explore ecological adaptations, and expand molecular datasets for a more comprehensive understanding of *Funiliomycetaceae*.

Species within *Funiliomycetaceae* exhibit considerable ecological diversity in lifestyle, host range, and geographical distribution. The family includes endophytic, saprobic, and epiphytic species, colonizing hosts from a broad spectrum of plant families such as *Fabaceae*, *Ericaceae*, *Poaceae*, and *Rutaceae*, as well as decaying unidentified leaves or ornamentals, indicating no strict host specificity. Geographically, *Funiliomycetaceae* species are widely distributed across multiple continents, including North America, Asia, and South America, and occur in diverse climatic zones ranging from temperate to tropical regions (Table 4). By contrast, most *Nothodactylariaceae* species are saprobic, with the exception of *Nothodactylaria nephrolepidis*, which is epiphytic. They show a strong preference for ferns (e.g., *Blechnaceae*, *Lomariopsidaceae*) and are known only from China and South Africa, mostly in tropical and subtropical climates (Zhang et al. 2025). These marked differences in host range, ecological strategy, geographical distribution, and climatic preference underscore the distinct ecological niche occupied by *Funiliomycetaceae*. With the addition of *Funiliomycetaceae*, the order now comprises 21 families, highlighting both the considerable diversity within *Amphisphaeriales* and the presence of previously unrecognized lineages.

The new family comprises *Funiliomyces biseptatus*, ten previously described “*Dactylaria*” species, and our newly obtained isolates. As confirmed by phylogenetic analyses (Fig. 1), these ten “*Dactylaria*” species are not congeneric with the type of *Dactylaria* (*D. purpurella*) but form a coherent, monophyletic group with *F. biseptatus*. This supports their recognition as a single genus, consistent with the “One Fungus, One Name” principle (Hawksworth 2012) and the general taxonomic guideline of recognizing monophyletic genera with coherent morphological characters. Accordingly, we transfer the ten previously described “*Dactylaria*” species into the genus *Funiliomyces* as new combinations (*F. acaciae*, *F. bisepatus*, *F. calliandrae*, *F. fragilis*, *F. hwasunensis*, *F. mavisleverae*, *F. monticola*, *F. retrophylli*, *F. sparsus*, and *F. zapatensis*). Furthermore, *Funiliomyces* is designated as the type genus of *Funiliomycetaceae*. The genus was described by Aptroot (2004), containing only its type species, *F. biseptatus*, which was collected from dead leaves of a *Bromeliaceae* species in a rock field. This species differs from the other newly transferred species in the genus by possessing only a sexual morph. In the present study, we summarize the asexual morphological features, lifestyle, host associations, and distribution of *Funiliomyces* species (Table 4), providing a comprehensive framework for future identification, ecological studies, and taxonomic research of the genus.

In addition to transferring ten “*Dactylaria*” species to *Funiliomyces*, we describe a new species (*F. jiangxiensis*) that further enriches the diversity of this genus. Multi-gene phylogenetic analyses also indicate that the new isolates, *Funiliomyces jiangxiensis*, occupies a distinct position within *Funiliomyces*, supported by high statistical values (SH-aLRT/UFB/PP = 98.8/100/0.90). Morphologically, *F. jiangxiensis* can be distinguished from its closely related species (*F. hwasunensis* and *F. calliandrae*) by differences in conidiophore structure, conidiogenous cell denticulation, and conidial characteristics (Table 4). Additionally, *Funiliomyces jiangxiensis* was isolated from the medicinal plant *Tetradium ruticarpum*. This plant, commonly known as *Evodia*, is renowned for its therapeutic properties, including anti-inflammatory, analgesic, and antimicrobial activities (Xiao et al. 2023). Endophytes have been studied and are known to produce novel biologically active compounds (Liao et al. 2025). For example, *Funiliomyces hwasunensis* CMML 20-35, isolated from the roots of *Zoysia japonica*, inhibited the mycelial growth of *Rhizoctonia solani* AG2-2(IIIB) by 37.97% in dual culture (Liu et al. 2025). To date, endophytic fungal studies on this host are limited, with only a few taxa reported recently, including *Cyphellophora guangxiensis* and *Pseudokeissleriella tetradii* (Mi et al. 2025a, 2025b). These findings emphasize the need for comprehensive surveys and further functional studies to explore the diversity and antimicrobial capabilities of endophytic fungi associated with this medicinal plant.

## Supplementary Material

XML Treatment for Funiliomycetaceae

XML Treatment for Funiliomyces

XML Treatment for Funiliomyces jiangxiensis

XML Treatment for Funiliomyces acaciae

XML Treatment for Funiliomyces bisepatus

XML Treatment for Funiliomyces calliandrae

XML Treatment for Funiliomyces fragilis

XML Treatment for Funiliomyces hwasunensis

XML Treatment for Funiliomyces mavisleverae

XML Treatment for Funiliomyces monticola

XML Treatment for Funiliomyces retrophylli

XML Treatment for Funiliomyces sparsus

XML Treatment for Funiliomyces zapatensis

## Additional information

### Use of AI

No use of AI was reported.

### Adherence to national and international regulations

All the fungal strains used in this study have been legally obtained, respecting the Convention on Biological Diversity (Rio Convention).

### Author ORCIDs

Li-Xue Mi https://orcid.org/0009-0009-0691-7171

Dian-Ming Hu https://orcid.org/0000-0002-4750-2871

Kevin D. Hyde https://orcid.org/0000-0002-2191-0762

Prapassorn D. Eungwanichayapant https://orcid.org/0000-0001-8005-4137

Ausana Mapook https://orcid.org/0000-0001-7929-2429

Danushka S. Tennakoon https://orcid.org/0000-0003-2306-1255

Jing-Yi Zhang https://orcid.org/0000-0003-0606-6169

Hai-Yan Song https://orcid.org/0009-0001-4140-5720

### Data availability

All nucleotide sequences of the species described in this work were deposited in GenBank. The accession numbers of the sequences deposited in GenBank are as follows: ITS: OQ869213, OQ869215; LSU: OQ869216, OQ869214; RPB2: OR046688, OR046687. All phylogenetic datasets generated and analyzed during the present study are publicly available in the TreeBASE repository. Specifically, the dataset corresponding to Fig. 1 is archived under Submission No. TB2:S32460 (URL: http://purl.org/phylo/treebase/phylows/study/TB2:S32460), and the dataset for Fig. 2 is available under Submission No. TB2:S32462 (URL: http://purl.org/phylo/treebase/phylows/study/TB2:S32462).

## Supplementary materials

Supplementary material 1 Alignments for *Pezizomycotina* and *Amphisphaeriales* phylogenetic analyses Data type zip Explanation note Two alignment files for the multi-gene phylogenetic analyses (*Pezizomycotina* and *Amphisphaeriales*) in this study. https://binary.pensoft.net/file/1534206This dataset is made available under the Open Database License (http://opendatacommons.org/licenses/odbl/1.0/). The Open Database License (ODbL) is a license agreement intended to allow users to freely share, modify, and use this Dataset while maintaining this same freedom for others, provided that the original source and author(s) are credited. Li-Xue Mi, Dian-Ming Hu, Kevin D. Hyde, Prapassorn D. Eungwanichayapant, Ausana Mapook, Danushka S. Tennakoon, Jing-Yi Zhang, Hai-Yan Song

## References

[B1] Aptroot A (2004) Two new ascomycetes with long gelatinous appendages collected from monocots in the tropics. Studies in Mycology 50: 1–27.

[B2] Barbosa FR, Raja HA, Shearer CA et al. (2013) Some freshwater fungi from the Brazilian semi-arid region, including two new species of hyphomycetes. Cryptogamie, Mycologie 34(3): 243–258. 10.7872/crym.v34.iss2.2013.243

[B3] Becerra-Hernández CI, González D, De Luna E et al. (2016) First report of pleoanamorphy in *Gyrothrix verticiclada* with an Idriella-like synanamorph. Cryptogamie, Mycologie 37(2): 241–252. 10.7872/crym/v37.iss2.2016.241

[B4] Beimforde C, Schmidt AR, Rikkinen J et al. (2020) *Sareomycetes* cl. nov.: A new proposal for placement of the resinicolous genus *Sarea* (*Ascomycota*, *Pezizomycotina*). Fungal Systematics and Evolution 6: 25–37. 10.3114/fuse.2020.06.02 PMC7451776 32904095

[B5] Bhatt G, Kendrick B (2011) The generic concept of *Diplorhinotrichum* and *Dactylaria*, and a new species of *Dactylaria* from soil. Canadian Journal of Botany 46: 1253–1257. 10.1139/b68-167

[B6] Bhattacharya D, Lutzoni F, Reeb V et al. (2000) Widespread occurrence of spliceosomal introns in the rDNA genes of ascomycetes. Molecular Biology and Evolution 17(12): 1971–1984. 10.1093/oxfordjournals.molbev.a026298 11110913

[B7] Bussaban B, Lumyong S, Lumyong P et al. (2005) Molecular and morphological characterization of *Pyricularia* and allied genera. Mycologia 97: 1002–1011. 10.3852/mycologia.97.5.1002 16596952

[B8] Castañeda Ruíz RF (1988) Fungi cubenses III. Instituto de Investigaciones *Fundamentales* en Agricultura Tropical “Alejandro de Humboldt”, 1–27. http://www.cybertruffle.org.uk/cyberliber/03416/cfo_.htm

[B9] Castañeda Ruíz RF, Kendrick B (1991) Ninety-nine conidial fungi from Cuba and three from Canada. University of Waterloo Biology Series 35: 1–132.

[B10] Chandra H, Yadav A, Prasad R et al. (2024) Fungal endophytes from medicinal plants acting as natural therapeutic reservoir. The Microbe 3: 100073. 10.1016/j.microb.2024.100073

[B11] Chinese Pharmacopoeia Commission (2010) Pharmacopoeia of the People’s Republic of China. China Medical Science and Technology Press, Beijing, 160 pp.

[B12] Cooper JA (2005) New Zealand hyphomycete fungi: Additional records, new species, and notes on interesting collections. New Zealand Journal of Botany 43(1): 323–349. 10.1080/0028825X.2005.9512957

[B13] Crespo A, Lumbsch HT, Mattsson JE et al. (2007) Testing morphology-based hypotheses of phylogenetic relationships in *Parmeliaceae (Ascomycota)* using three ribosomal markers and the nuclear RPB1 gene. Molecular Phylogenetics and Evolution 44(2): 812–824. 10.1016/j.ympev.2006.11.029 17276700

[B14] Currie CR, Wong B, Stuart AE et al. (2003) Ancient tripartite coevolution in the attine ant-microbe symbiosis. Science 299(5605): 386–388. 10.1126/science.1078155 12532015

[B15] Crous PW, Begoude BAD, Boers J et al. (2022) New and Interesting Fungi. 5. Fungal Systematics and Evolution 10: 19–90. 10.3114/fuse.2022.10.02 PMC9903348 36789279

[B16] Crous PW, Carnegie AJ, Wingfield MJ et al. (2019a) Fungal Planet description sheets: 868–950. Persoonia 42: 291–473. 10.3767/persoonia.2019.42.11 PMC6712538 31551622

[B17] Crous PW, Carris LM, Giraldo A et al. (2015a) The Genera of Fungi - fixing the application of the type species of generic names - G 2: *Allantophomopsis*, *Latorua*, *Macrodiplodiopsis*, *Macrohilum*, *Milospium*, *Protostegia*, *Pyricularia*, *Robillarda*, *Rotula*, Sept*o*riella, *Torula*, and *Wojnowicia*. IMA Fungus 6(1): 163–198. 10.5598/imafungus.2015.06.01.11 PMC4500082 26203422

[B18] Crous PW, Catcheside DEA, Catcheside PS et al. (2025) Fungal Planet description sheets: 1781–1866. Persoonia 54: 327–587. 10.3114/persoonia.2025.54.10 PMC12308287 40746709

[B19] Crous PW, Luangsa-Ard JJ, Wingfield MJ et al. (2018a) Fungal Planet description sheets: 785–867. Persoonia 41: 238–417. 10.3767/persoonia.2018.41.12 PMC6344811 30728607

[B20] Crous PW, Shivas RG, Quaedvlieg W et al. (2014a) Fungal Planet description sheets: 214–280. Persoonia 32: 184–306. 10.3767/003158514X682395 PMC4150077 25264390

[B21] Crous PW, Summerell BA, Shivas RG et al. (2011a) Fungal Planet description sheets: 92–106. Persoonia 27: 130–162. 10.3767/003158511X617561 PMC3251320 22403481

[B22] Crous PW, Summerell BA, Shivas RG et al. (2012) Fungal Planet description sheets: 107–127. Persoonia 28: 138–182. 10.3767/003158512X652633 PMC3409410 23105159

[B23] Crous PW, Tanaka K, Summerell BA et al. (2011b) Additions to the Mycosphaerella complex. IMA Fungus 2(1): 49–64. 10.5598/imafungus.2011.02.01.08 PMC3317358 22679588

[B24] Crous PW, Wingfield MJ, Burgess TI et al. (2016) Fungal Planet description sheets: 469–557. Persoonia 37: 218–403. 10.3767/003158516X694499 PMC5315290 28232766

[B25] Crous PW, Wingfield MJ, Burgess TI et al. (2017) Fungal Planet description sheets: 625–715. Persoonia 39: 270–467. 10.3767/persoonia.2017.39.11 PMC5832955 29503478

[B26] Crous PW, Wingfield MJ, Guarro J et al. (2015b) Fungal Planet description sheets: 320–370. Persoonia 34: 167–266. 10.3767/003158515X688433 PMC4510277 26240451

[B27] Crous PW, Wingfield MJ, Jurjević Ž et al. (2024) Fungal Planet description sheets: 1697–1780. Fungal Syst Evol 14: 325–577. 10.3114/fuse.2024.14.19 PMC11736264 39830292

[B28] Crous PW, Wingfield MJ, Lombard L et al. (2019b) Fungal Planet description sheets: 951–1041. Persoonia 43: 223–425. 10.3767/persoonia.2019.43.06 PMC7085856 32214501

[B29] Crous PW, Wingfield MJ, Schumacher RK et al. (2014b) Fungal Planet description sheets: 281–319. Persoonia 33: 212–289. 10.3767/003158514X685680 PMC4312934 25737601

[B30] Crous PW, Wingfield MJ, Schumacher RK et al. (2018b) New and Interesting Fungi. 1. Fungal Systematics and Evolution 1: 169–216. 10.3767/persoonia.2019.43.06 PMC7259438 32490366

[B31] Crous PW, Wingfield MJ, Schumacher RK et al. (2020) New and Interesting Fungi. 3. Fungal Syst Evol 6: 157–231. 10.3114/fuse.2020.06.09 PMC7452156 32904192

[B32] Daros-Pawlyta J, Pawlyta A (2023) The concept of legal interest in the context of the possibility of challenging an air protection programme before an administrative court. Remarks on the CJEU judgment of 22 December 2022, C 61/21. Kwartalnik Prawa Międzynarodowego I(I): 217–251. Polish. 10.5604/01.3001.0016.3295

[B33] Dissanayake LS, Samarakoon MC, Maharachchikumbura SSN et al. (2024) Exploring the taxonomy and phylogeny of *Sordariomycetes* taxa emphasizing *Xylariomycetidae* in Southwestern China. Mycosphere 15(1): 1675–1793. 10.5943/mycosphere/15/1/15

[B34] Deng Z, Zhang R, Shi Y et al. (2014) Characterization of Cd-, Pb-, Zn-resistant endophytic *Lasiodiplodia* sp. MXSF31 from metal accumulating *Portulaca oleracea* and its potential in promoting the growth of rape in metal-contaminated soils. Environmental Science and Pollution Research 21(3): 2436–2457. 10.1007/s11356-013-2163-2 24062066

[B35] de Hoog GS (1985) Taxonomy of the *Dactylaria*-complex, IV. *Dactylaria*, *Neta*, *Subulispora* and *Scolecobasidium*. Studies in Mycology 26: 1–60.

[B36] de Hoog GS, van Oorschot CAN (1985) Taxonomy of the *Dactylaria* complex, VI. Key to the genera and check-list of epithets. Studies in Mycology 26: 97–121.

[B37] de Souza MGAP, Costa-Rezende DH, Castañeda-Ruiz RF et al. (2025) Corrigendum to: A morphological and phylogenetic analysis of *Subulispora tubaki*. Australian Systematic Botany 38(3): SB2402. 10.1071/SB24029

[B38] Doyle JJ, Doyle JL (1987) A rapid DNA isolation procedure for small quantities of fresh leaf tissue. Phytochemical Bulletin 19(1): 11–15.

[B39] Eriksson OE, Hawksworth DL (1986) Notes on ascomycete systematics. Nos. 1-224. Systema Ascomycetum 5: 113–174.

[B40] Flavia RB, Huzefa AR, Carol AS et al. (2013) Some freshwater fungi from the Brazilian Semi-Arid Region, including two new species of hyphomycetes. Cryptogamie, Mycologie 34(3): 243–258. 10.7872/crym.v34.iss2.2013.243

[B41] Gazis R, Miadlikowska J, Lutzoni F et al. (2012) Culture-based study of endophytes associated with rubber trees in Peru reveals a new class of *Pezizomycotina*: *Xylonomycetes*. Molecular Phylogenetics and Evolution 65(1): 294–304. 10.1016/j.ympev.2012.06.019 22772026

[B42] Geiser DM, Gueidan C, Miadlikowska J et al. (2006) *Eurotiomycetes*: *Eurotiomycetidae* and *Chaetothyriomycetidae*. Mycologia 98(6): 1053–1064. 10.3852/mycologia.98.6.1053 17486980

[B43] Giraldo A, Crous PW, Schumacher RK et al. (2017) The Genera of Fungi—G3: *Aleurocystis*, *Blastacervulus*, *Clypeophysalospora*, Licr*o*stroma, *Neohendersonia* and *Spumatoria*. Mycological Progress 16: 325–348. 10.1007/s11557-017-1270-8

[B44] Goh TK, Hyde KD (1997) A revision of *Dactylaria*, with description of *D. tunicata* sp. nov. from submerged wood in Australia. Mycological Research 101(10): 1265–1272. 10.1017/s0953756297004000

[B45] Gueidan C, Villaseñor CR, de Hoog GS et al. (2008) A rock-inhabiting ancestor for mutualistic and pathogen-rich fungal lineages. Studies in Mycology 61: 111–119. 10.3114/sim.2008.61.11 PMC2610302 19287533

[B46] Guindon S, Dufayard JF, Lefort V et al. (2010) New algorithms and methods to estimate maximum-likelihood phylogenies: assessing the performance of PhyML 3.0. Systematic Biology 59(3): 307–321. 10.1093/sysbio/syq010 20525638

[B47] Gupta A, Meshram V, Gupta M et al. (2023) Fungal endophytes: Microfactories of novel bioactive compounds with therapeutic interventions; a comprehensive review on thebiotechnological developments in the field of fungal endophytic biology over the last decade. Biomolecules 13(7): 1038. 10.3390/biom13071038 PMC10377637 37509074

[B48] Hall TA (1999) BioEdit: A user-friendly biological sequence alignment editor and analysis program for Windows 95/98/NT. Nucleic Acids Symposium Series 41: 95–98.

[B49] Haelewaters D, De Kesel A, Gorczak M et al. (2019a) *Laboulbeniales (Ascomycota)* of the Boston Harbor Islands II (and other localities): species parasitizing *Carabidae*, and the *Laboulbenia flagellata* species complex. Northeastern Naturalist 25(sp9): 110–149. 10.1656/045.025.s906

[B50] Haelewaters D, Pfliegler WP, Gorczak M et al. (2019b) Birth of an order: Comprehensive molecular phylogenetic study excludes *Herpomyces* (*Fungi*, *Laboulbeniales*) from *Laboulbeniales*. Molecular Phylogenetics and Evolution 133: 286–301. 10.1016/j.ympev.2019.01.007 30625361

[B51] Hawksworth DL (2012) Managing and coping with names of pleomorphic fungi in a period of transition. Mycosphere 3(2): 143–155. 10.5943/mycosphere/3/2/4/PMC3399099 23155497

[B52] Hernández-Restrepo M, Gené J, Castañeda-Ruiz RF et al. (2017) Phylogeny of saprobic microfungi from Southern Europe. Studies in Mycology 86: 53–97. 10.1016/j.simyco.2017.05.002 PMC5470572 28626275

[B53] Hongsanan S, Hyde KD (2017) Phylogenetic placement of *Micropeltidaceae*. Mycosphere 8(10): 1930–1942. 10.5943/mycosphere/8/10/15

[B54] Hongsanan S, Maharachchikumbura SSN, Hyde KD et al. (2017) An updated phylogeny of *Sordariomycetes* based on phylogenetic and molecular clock evidence. Fungal Diversity 84: 25–41. 10.1007/s13225-017-0384-2

[B55] Ho MY, Chung WC, Huang HC et al. (2012) Identification of endophytic fungi of medicinal herbs of *Lauraceae* and *Rutaceae* with antimicrobial property. Taiwania 57: 229–241. 10.6165/tai.2012.57(3).229

[B56] Hoang D, Chernomor O, von Haeseler A et al. (2017) UFBoot2: Improving the ultrafast bootstrap approximation. Molecular Biology and Evolution 35: 1–10. 10.1093/molbev/msx281 PMC5850222 29077904

[B57] Hyde KD, Noorabadi MT, Thiyagaraja V et al. (2024) The 2024 Outline of fungi and fungus-like taxa. Mycosphere 15(1): 5146–6239. 10.5943/mycosphere/15/1/25

[B58] Hyde KD, Norphanphoun C, Maharachchikumbura SSN et al. (2020) Refined families of *Sordariomycetes*. Mycosphere 11(1): 305–1059. 10.5943/mycosphere/11/1/7

[B59] Index Fungorum (2025) http://www.indexfungorum.org [Accessed on Ausust 2025]

[B60] Jaklitsch WM, Gardiennet A, Voglmayr H et al. (2016) Resolution of morphology-based taxonomic delusions: *Acrocordiella*, *Basiseptospora*, *Blogiascospora*, *Clypeosphaeria*, *Hymenopleella*, *Lepteutypa*, *Pseudapiospora*, *Requienella*, *Seiridium* and *Strickeria*. Persoonia 37: 82–105. 10.3767/003158516X690475 PMC5238940 28100927

[B61] Jaklitsch WM, Voglmayr H (2012) Phylogenetic relationships of five genera of *Xylariales* and *Rosasphaeria* gen. nov. (*Hypocreales*). Fungal Diversity 52: 75–98. 10.1007/s13225-011-0104-2

[B62] James TY, Kauff F, Schoch CL et al. (2006) Reconstructing the early evolution of *Fungi* using a six-gene phylogeny. Nature 443(7113): 818–822. 10.1038/nature05110 17051209

[B63] Jayasiri SC, Hyde KD, Ariyawansa HA et al. (2015) The Faces of Fungi database: fungal names linked with morphology, phylogeny and human impacts. Fungal Diversity 74: 3–18. 10.1007/s13225-015-0351-8

[B64] Jeewon R, Liew EC, Hyde KD et al. (2003) Molecular systematics of the *Amphisphaeriaceae* based on cladistic analyses of partial LSU rDNA gene sequences. Mycological Research 107(Pt 12): 1392–1402. 10.1017/s095375620300875x 15000240

[B65] Katoh K, Standley DM (2013) MAFFT multiple sequence alignment software version 7: improvements in performance and usability. Molecular Biology and Evolution 30(4): 772–780. 10.1093/molbev/mst010 PMC3603318 23329690

[B66] Konta S, Hongsanan S, Tibpromma S et al. (2016) An advance in the endophyte story: *Oxydothidaceae* fam. nov. with six new species of *Oxydothis*. Mycosphere 7(9): 1425–1446. 10.5943/mycosphere/7/9/15

[B67] Klaubauf S, Tharreau D, Fournier E et al. (2014) Resolving the polyphyletic nature of *Pyricularia (Pyriculariaceae)*. Studies in Mycology 79: 85–120. 10.1016/j.simyco.2014.09.004 PMC4255532 25492987

[B68] Kralovic SM, Rhodes JC (1995) Phaeohyphomycosis caused by *Dactylaria* (human dactylariosis): Report of a case with review of the literature. Journal of Infection 31(2): 107–113. 10.1016/S0163-4453(95)92060-9 8666840

[B69] Krizková L, Balan J, Nemec P et al. (1976) Predacious fungi *Dactylaria pyriformis* and *Dactylaria thaumasia*: Production of attractants and nematicides. Folia Microbiologica 21(6): 493–494. 10.1007/BF02876942 1033116

[B70] Kurtzman CP, Robnett CJ (2013) Relationships among genera of the *Saccharomycotina (Ascomycota)* from multigene phylogenetic analysis of type species. FEMS Yeast Research 13(1): 23–33. 10.1111/1567-1364.12006 22978764

[B71] Kumar N, Singh K (2011) Use of *Dactylaria brochopaga*, a predacious fungus, for managing root-knot disease of wheat (*Triticum aestivum*) caused by *Meloidogyne graminicola*. Mycobiology 39: 113–117. 10.4489/MYCO.2011.39.2.113 PMC3385099 22783087

[B72] Kumar S, Stecher G, Li M et al. (2018) MEGA X: Molecular evolutionary genetics analysis across computing platforms. Molecular Biology and Evolution 35(6): 1547–1549. 10.1093/molbev/msy096 PMC5967553 29722887

[B73] Kusari S, Lamshöft M, Spiteller M (2009) *Aspergillus fumigatus* Fresenius, an endophytic fungus from *Juniperus communis* L. Horstmann as a novel source of the anticancer pro-drug deoxypodophyllotoxin. Journal of Applied Microbiology 107(3): 1019–1030. 10.1111/j.1365-2672.2009.04285.x 19486398

[B74] Li ML, Wang CH (2020) Traditional uses, phytochemistry, pharmacology, pharmacokinetics and toxicology of the fruit of *Tetradium ruticarpum*: A review. Journal of Ethnopharmacology 263: 113231. 10.1016/j.jep.2020.113231 32758577

[B75] Li Q, Kang JC, Hyde KD (2015) A multiple gene genealogy reveals the phylogenetic placement of *Iodosphaeria tongrenensis* sp. nov. in *Iodosphaeriaceae (Xylariales)*. Phytotaxa 234(2): 121–132. 10.11646/phytotaxa.234.2.2

[B76] Li Y, Jeewon R, Hyde KD et al. (2006) Two new species of nematode-trapping fungi: relationships inferred from morphology, rDNA and protein gene sequence analyses. Mycological Research 110(Pt 7): 790–800. 10.1016/j.mycres.2006.04.011 16876699

[B77] Lin CG, Dai DQ, Bhat DJ et al. (2017) *Subsessila turbinata* gen. et. sp. nov. (*Beltraniaceae*), a Beltrania-like fungus from Thailand. Mycological Progress 16: 393–401. 10.1007/s11557-017-1279-z

[B78] Lin LC, Tan YL, Lin WR et al. (2021) The effect of dark septate endophytic fungi on *Mahonia oiwakensis*. Plants 10: 1723. 10.3390/plants10081723 PMC8401445 34451768

[B79] Liao CF, Doilom M, Jeewon R et al. (2025) Challenges and update on fungal endophytes: classification, definition, diversity, ecology, evolution and functions. Fungal Diversity 131: 301–367. 10.1007/s13225-025-00550-5

[B80] Liu H, Choi H, Paul NC et al. (2025) Discovering fungal communities in roots of *Zoysia japonica* and characterising novel species and their antifungal activities. IMA Fungus 16: e138479. 10.3897/imafungus.16.138479 PMC11881003 40052078

[B81] Liu JW, Luangharn T, Wan SP et al. (2022) A new edible species of *Gomphus (Gomphaceae)* from southwestern China. Mycoscience 63(6): 293–297. 10.47371/mycosci.2022.09.002 PMC10026079 37089522

[B82] Liu YJ, Whelen S, Hall BD (1999) Phylogenetic relationships among ascomycetes: evidence from an RNA polymerase II subunit. Molecular Biology and Evolution 16(12): 1799–1808. 10.1093/oxfordjournals.molbev.a026092 10605121

[B83] Lombard L, van der Merwe NA, Groenewald JZ et al. (2015) Generic concepts in *Nectriaceae*. Studies in Mycology 80: 189–245. 10.1016/j.simyco.2014.12.002 PMC4779799 26955195

[B84] Lumbsch HT, Schmitt I, Lindemuth R et al. (2005) Performance of four ribosomal DNA regions to infer higher-level phylogenetic relationships of inoperculate euascomycetes (*Leotiomyceta*). Molecular Phylogenetics and Evolution 34(3): 512–524. 10.1016/j.ympev.2004.11.007 15683926

[B85] Lutzoni F, Kauff F, Cox CJ et al. (2004) Assembling the fungal tree of life: progress, classification, and evolution of subcellular traits. American Journal of Botany 91(10): 1446–1480. 10.3732/ajb.91.10.1446 21652303

[B86] Lutzoni F, Pagel M, Reeb V (2001) Major fungal lineages are derived from lichen symbiotic ancestors. Nature 411(6840): 937–940. 10.1038/35082053 11418855

[B87] Magalhães D, Luz E, Magalhães A et al. (2014) Anamorphic fungi of the Atlantic forest of southern Bahia: New records and *Dactylaria pseudomanifesta* sp. nov. Mycotaxon 128: 185–194. 10.5248/128.185

[B88] Marasinghe DS, Samarakoon MC, Hongsanan S et al. (2019) *Iodosphaeria honghensis* sp. nov. (*Iodosphaeriaceae*, *Xylariales*) from Yunnan Province, China. Phytotaxa 420(4): 273–282. 10.11646/phytotaxa.420.4.3

[B89] Matsushima T (1975) Icones Microfungorum a Matsushima Lectorum 1–209.

[B90] McLaughlin DJ, Spatafora JW (2014) Systematics and evolution: Part A. Springer Berlin Heidelberg 7: 245. 10.1007/978-3-642-55318-9

[B91] McCune B (2018) Two new species in the *Umbilicaria torrefacta* group from Alaska and the Pacific Northwest of North America. Graphis Scripta 30(6): 65–77.

[B92] McTaggart AR, Grice KR, Shivas RG (2013) First report of *Vialaea minutella* in Australia, its association with mango branch dieback and systematic placement of *Vialaea* in the *Xylariales*. Australasian Plant Disease Notes, Australasian Plant Pathology Society 8(1): 63–66. 10.1007/s13314-013-0096-8

[B93] Mel’nik VA, Braun U, Alexandrova AV (2013) *Dactylaria mucoglobifera* sp. nov. – a new species from Vietnam. Schlechtendalia 25: 49–52.

[B94] Miadlikowska J, Kauff F, Hofstetter V et al. (2006) New insights into classification and evolution of the *Lecanoromycetes* (*Pezizomycotina*, *Ascomycota*) from phylogenetic analyses of three ribosomal RNA- and two protein-coding genes. Mycologia 98(6): 1088–1103. 10.1080/15572536.2006.11832636 17486983

[B95] Miller AN, Huhndorf SM (2005) Multi-gene phylogenies indicate ascomal wall morphology is a better predictor of phylogenetic relationships than ascospore morphology in the *Sordariales* (*Ascomycota*, *Fungi*). Molecular Phylogenetics and Evolution 35(1): 60–75. 10.1016/j.ympev.2005.01.007 15737582

[B96] Miller MA, Pfeiffer W, Schwartz T (2010) Creating the CIPRES Science Gateway for inference of large phylogenetic trees. In: Cordero Rivera A (Ed.) Proceedings of the Gateway Computing Environments Workshop (GCE). Institute of Electrical and Electronics Engineers, New Orleans, 1–8. 10.1109/GCE.2010.5676129

[B97] Mi LX, Song HY, Eungwanichayapant PD et al. (2025a) Integrative taxonomy reveals *Pseudokeissleriella tetradii* sp. nov. (*Lentitheciaceae*, *Pleosporales*) associated with *Tetradium ruticarpum* in Anhui Province, China. Phytotaxa 704(3): 239–254. 10.11646/phytotaxa.704.3.3

[B98] Mi LX, Song HY, Hyde KD et al. (2025b) Morphological and phylogenetic characterization of a new *Cyphellophora* (*Chaetothyriales*, *Cyphellophoraceae*) species associated with *Tetradium ruticarpum* from Guangxi Province, China. Phytotaxa 704(3): 239–254. 10.11646/phytotaxa.704.3.3

[B99] Nylander JAA (2004) MrModeltest v2. Program distributed by the author. Evolutionary Biology Centre, Uppsala University.

[B100] Paulus B, Gadek P, Hyde KD (2003) Two new species of *Dactylaria* (anamorphic fungi) from Australian rainforests and an update of species in *Dactylaria* sensu lato. Fungal Diversity 14: 1–20.

[B101] Perera RH, Maharachchikumbura SS, Hyde KD et al. (2018) An appendage-bearing coelomycete *Pseudotruncatella arezzoensis* gen. and sp. nov. (*Amphisphaeriales* genera *incertae sedis*) from Italy, with notes on *Monochaetinula*. Phytotaxa 338: 177–188. 10.11646/phytotaxa.338.2.2

[B102] Pérez-Ortega S, Garrido-Benavent I, Grube M et al. (2016) Hidden diversity of marine borderline lichens and a new order of fungi: *Collemopsidiales (Dothideomyceta)*. Fungal Diversity 80: 285–300. 10.1007/s13225-016-0361-1

[B103] Pinnoi A, Jones EBG, McKenzie EHC et al. (2003) Aquatic fungi from peat swamp palms: *Unisetosphaeria penguinoides* gen. et sp. nov., and three new *Dactylaria* species. Mycoscience 44(5): 377–382. 10.1007/S10267-003-0124-1

[B104] Pintos Á, Alvarado P, Planas J et al. (2019) Six new species of *Arthrinium* from Europe and notes about *A. caricicola* and other species found in *Carex* spp. hosts. MycoKeys 49: 15–48. 10.3897/mycokeys.49.32115 PMC6424953 30918449

[B105] Prieto M, Baloch E, Tehler A et al. (2013) Mazaedium evolution in the *Ascomycota (Fungi)* and the classification of mazaediate groups of formerly unclear relationship. Cladistics 29(3): 296–308. 10.1111/j.1096-0031.2012.00429.x 34818827

[B106] Rambaut A (2018) FigTree, a graphical viewer of phylogenetic trees (Version 1.4.4). Institute of Evolutionary Biology, University of Edinburgh, Edinburgh, UK.

[B107] Rannala B, Yang Z (1996) Probability distribution of molecular evolutionary trees: a new method of phylogenetic inference. Journal of Molecular Evolution 43: 304–311. 10.1007/BF02338839 8703097

[B108] Réblová M (2009) Teleomorph of Rhodoveronaea (*Sordariomycetidae*) discovered and re-evaluation of *Pleurophragmium*. Fungal Diversity 36: 129–139.

[B109] Réblová M, Kolařík M, Nekvindová J et al. (2021) Phylogeny, global biogeography and pleomorphism of *Zanclospora*. Microorganisms 9: 706. 10.3390/microorganisms9040706 PMC8066784 33805574

[B110] Réblová M, Seifert KA (2012) Cirrosporium novae-zelandiae, an enigmatic coelomycete with meristem arthroconidia, with ancestors in the *Eurotiomycetes*. Mycologia 104(6): 1315–1324. 10.3852/12-040 22675053

[B111] Reeb V, Lutzoni F, Roux C et al. (2004) Contribution of RPB2 to multilocus phylogenetic studies of the euascomycetes (*Pezizomycotina*, *Fungi*) with special emphasis on the lichen-forming *Acarosporaceae* and evolution of polyspory. Molecular Phylogenetics and Evolution 32(3): 1036–1060. 10.1016/j.ympev.2004.04.012 15288074

[B112] Ronquist F, Teslenko M, Mark P et al. (2012) MrBayes 3.2: Efficient Bayesian phylogenetic inference and model choice across a large model space. Systematic Biology 61(3): 539–542. 10.1093/sysbio/sys029 PMC3329765 22357727

[B113] Saccardo PA (1880) Conspectus generum fungorum Italiae inferiorum nempe ad *Sphaeropsideas*, *Melanconieas* et *Hyphomyceteas* pertinentium systemate sporologico dispositorum. Michelia 2(6): 1–38.

[B114] Samarakoon MC, Hyde KD, Maharachchikumbura SSN et al. (2022) Taxonomy, phylogeny, molecular dating and ancestral state reconstruction of *Xylariomycetidae (Sordariomycetes)*. Fungal Diversity 112: 1–88. 10.1007/s13225-021-00495-5

[B115] Samarakoon MC, Hyde KD, Promputtha I et al. (2016) Evolution of *Xylariomycetidae* (*Ascomycota*: *Sordariomycetes*). Mycosphere 7(11): 1746–1761. 10.5943/mycosphere/7/11/9

[B116] Samarakoon MC, Liu JK, Hyde KD et al. (2019) Two new species of *Amphisphaeria (Amphisphaeriaceae)* from northern Thailand. Phytotaxa 391(3): 207–217. 10.11646/phytotaxa.391.3.4

[B117] Schneider K, Resl P, Spribille T et al. (2016) Escape from the cryptic species trap: lichen evolution on both sides of a cyanobacterial acquisition event. Molecular Ecology 25(14): 3453–3468. 10.1111/mec.13636 PMC5324663 27037681

[B118] Schoch CL, Kohlmeyer J, Volkmann-Kohlmeyer B et al. (2006a) The halotolerant fungus *Glomerobolus gelineus* is a member of the *Ostropales*. Mycological Research 110(Pt 3): 257–263. 10.1016/j.mycres.2005.10.001 16431093

[B119] Schoch CL, Shoemaker RA, Seifert KA et al. (2006b) A multigene phylogeny of the *Dothideomycetes* using four nuclear loci. Mycologia 98(6): 1041–1052. 10.3852/mycologia.98.6.1041 17486979

[B120] Schoch CL, Sung GH, López-Giráldez F et al. (2009a) The *Ascomycota* tree of life: a phylum-wide phylogeny clarifies the origin and evolution of fundamental reproductive and ecological traits. Systems Biology 58(2): 224–239. 10.1093/sysbio/syp020 20525580

[B121] Schoch CL, Wang Z, Townsend JP et al. (2009b) *Geoglossomycetes cl*. nov., *Geoglossales ord*. nov. and taxa above class rank in the *Ascomycota* Tree of Life. Persoonia 22: 129–138. 10.3767/003158509X461486 PMC2776753 19915689

[B122] Senanayake IC, Maharachchikumbura SSN, Hyde KD et al. (2015) Towards unraveling relationships in *Xylariomycetidae (Sordariomycetes)*. Fungal Diversity 73: 73–144. 10.1007/s13225-015-0340-y

[B123] Senwanna C, Mapook A, Samarakoon MC et al. (2021) *Ascomycetes* on Para rubber (*Hevea brasiliensis*). Mycosphere 12(1): 1334–1512. 10.5943/mycosphere/12/1/18

[B124] Suh SO, Houseknecht JL, Gujjari P et al. (2013) *Scheffersomyces parashehatae* f.a., sp. nov., *Scheffersomyces xylosifermentans* f.a., sp. nov., *Candida broadrunensis* sp. nov. and *Candida manassasensis* sp. nov., novel yeasts associated with wood-ingesting insects, and their ecological and biofuel implications. International Journal of Systematic and Evolutionary Microbiology 63(Pt 11): 4330–4339. 10.1099/ijs.0.053009-0 24014624

[B125] Spatafora JW, Sung GH, Johnson D et al. (2006) A five-gene phylogeny of *Pezizomycotina*. Mycologia 98(6): 1018–1028. 10.3852/mycologia.98.6.1018 17486977

[B126] Species Fungorum (2025) https://www.speciesfungorum.org [Accessed on Ausust 2025]

[B127] Summerell BA, Groenewald JZ, Carnegie A et al. (2006) Eucalyptus microfungi known from culture. 2. *Alysidiella*, *Fusculina* and *Phlogicylindrium* genera nova, with notes on some other poorly known taxa. Fungal Diversity 23: 323–350.

[B128] Swofford D (2002) PAUP*-Phylogenetic analysis using parsimony (*and other methods). Version 4.0b10.

[B129] Swe A, Jeewon R, Pointing SB et al. (2008) Taxonomy and molecular phylogeny of *Arthrobotrys mangrovispora*, a new marine nematode-trapping fungal species. Botanica Marina 51(4): 331–338. 10.1515/BOT.2008.043

[B130] Tan YP, Shivas RG (2024) Nomenclatural novelties. Index of Australian Fungi 46 (ISBN 978-1-7636439-5-6), 1–17. 10.5281/zenodo.13905938

[B131] Tiwari P, Bae H (2022) Endophytic fungi: key insights, emerging prospects, and challenges in natural product drug discovery. Microorganism 10(2): 360. 10.3390/microorganisms10020360 PMC8876476 35208814

[B132] Trifinopoulos J, Nguyen LT, von Haeseler A et al. (2016) W-IQ-TREE: a fast online phylogenetic tool for maximum likelihood analysis. Nucleic Acids Research 44(W1): W232–W235. 10.1093/nar/gkw256 PMC4987875 27084950

[B133] Van Vooren N (2020) Validation de *Peziza martinicensis* sp. nov. (*Pezizales*). Ascomycete.org 12(2): 57–60. 10.25664/ART-0298

[B134] Vilgalys R, Hester M (1990) Rapid genetic identification and mapping of enzymatically amplified ribosomal DNA from several *Cryptococcus* species. Journal of Bacteriology 172(8): 4238–4246. 10.1128/jb.172.8.4238-4246.1990 PMC213247 2376561

[B135] Voglmayr H, Fournier J, Jaklitsch WM et al. (2019) Two new classes of *Ascomycota*: *Xylobotryomycetes* and *Candelariomycetes*. Persoonia 42: 36–49. 10.3767/persoonia.2019.42.02 PMC6712537 31551613

[B136] Vu D, Groenewald M, de Vries M et al. (2019) Large-scale generation and analysis of filamentous fungal DNA barcodes boosts coverage for kingdom fungi and reveals thresholds for fungal species and higher taxon delimitation. Studies in Mycology 92: 135–154. 10.1016/j.simyco.2018.05.001 PMC6020082 29955203

[B137] Wang XW, Houbraken J, Groenewald JZ et al. (2016) Diversity and taxonomy of *Chaetomium* and chaetomium-like fungi from indoor environments. Studies in Mycology 84: 145–224. 10.1016/j.simyco.2016.11.005 PMC5226397 28082757

[B138] Wedin M, Wiklund E, Crewe A et al. (2005) Phylogenetic relationships of *Lecanoromycetes (Ascomycota)* as revealed by analyses of mtSSU and nLSU rDNA sequence data. Mycological Research 109(Pt 2): 159–172. 10.1017/s0953756204002102 15839100

[B139] Wen J, Okyere SK, Wang S et al. (2022) Endophytic fungi: an effective alternative source of plant-derived bioactive compounds for pharmacological studies. Journal of Fungi 8(2): 205. 10.3390/jof8020205 PMC8877053 35205959

[B140] White TJ, Bruns T, Lee S et al. (1990) Amplification and direct sequencing of fungal ribosomal RNA genes for phylogenetics. In: PCR Protocols, a Guide to Methods and Applications, 315–322. 10.1016/B978-0-12-372180-8.50042-1

[B141] Wijesinghe SN, Zucconi L, Camporesi E et al. (2022) An updated account of Fagales-inhabiting Italian *Ascomycota* and mycogeography, with additions to *Pezizomycotina*. Asian Journal of Mycology 5(2): 79–186. 10.5943/ajom/5/2/7

[B142] Wood AR, Damm U, van der Linde EJ et al. (2016) Finding the missing link: Resolving the *Coryneliomycetidae* within *Eurotiomycetes*. Persoonia 37: 37–56. 10.3767/003158516X689800 PMC5315291 28232760

[B143] Wu W, Sutton BC, Gange AC (1996) *Dactylaria endophytica* sp. nov., an endophyte from leaves of *Prunus lusitanica*. Mycological Research 100(5): 524–526. 10.1016/S0953-7562(96)80002-5

[B144] Xiao SJ, Xu XK, Chen W et al. (2023) Traditional Chinese medicine Euodiae fructus: botany, traditional use, phytochemistry, pharmacology, toxicity and quality control. Natural Products and Bioprospecting 13(1): 6. 10.1007/s13659-023-00369-0 PMC9931992 36790599

[B145] Xie J, Chen Y, Cai G et al. (2023) Tree Visualization By One Table (tvBOT): a web application for visualizing, modifying and annotating phylogenetic trees. Nucleic Acids Research 51(W1): W587–W592. 10.1093/nar/gkad359 PMC10320113 37144476

[B146] Xie L, Chen YL, Long YY et al. (2019) Three new species of *Conlarium* from sugarcane rhizosphere in southern China. MycoKeys 56: 1–11. 10.3897/mycokeys.56.35857 PMC6626063 31327928

[B147] Yang CL, Xu XL, Liu YG et al. (2019) First report of bamboo blight disease caused by *Arthrinium pseudoparenchymaticum* on *Dendrocalamus latiflorus* in Sichuan, China. Plant Disease 103(6): 1411. 10.1094/PDIS-01-19-0029-PDN

[B148] Zhang D, Gao F, Jakovlić I et al. (2020) PhyloSuite: An integrated and scalable desktop platform for streamlined molecular sequence data management and evolutionary phylogenetics studies. Molecular Ecology Resources 20(1): 348–355. 10.1111/1755-0998.13096 31599058

[B149] Zhang JY, Hyde KD, Bao DF et al. (2025) A worldwide checklist and morpho-molecular systematics of fungi associated with pteridophytes. Fungal Diversity 132: 151–423. 10.1007/s13225-025-00554-1

[B150] Zhu QL (2007) The study on second metabolites of an endophytic fungus from *Evodia rutaecarpa* and chemical constituents of *Lysimachia lobelioides* Wall. Master’s Thesis, Guizhou University, China, 33–34.

[B151] Zare R, Gams W (2016) More white verticillium-like anamorphs with erect conidiophores. Mycological Progress 15: 993–1030. 10.1007/s11557-016-1214-8

