# Corrigendum: Mi L-X, Hu D-M, Hyde KD, Eungwanichayapant PD, Mapook A, Tennakoon DS, Zhang J-Y, Song H-Y (2026) *Funiliomycetaceae* fam. nov. (*Amphisphaeriales*, *Ascomycota*) accommodating *Funiliomyces*, including *F. jiangxiensis* sp. nov. from Tetradium ruticarpum and ten new combinations. IMA Fungus 17: e179140. https://doi.org/10.3897/imafungus.17.179140

**DOI:** 10.3897/imafungus.17.195349

**Published:** 2026-05-11

**Authors:** Li-Xue Mi, Dian-Ming Hu, Kevin D. Hyde, Prapassorn D. Eungwanichayapant, Ausana Mapook, Danushka S. Tennakoon, Jing-Yi Zhang, Hai-Yan Song

**Affiliations:** 1 School of Agricultural Sciences, Jiangxi Agricultural University, Nanchang 330045, China Bioengineering and Technological Research Centre for Edible and Medicinal Fungi, Jiangxi Agricultural University Nanchang China; 2 Bioengineering and Technological Research Centre for Edible and Medicinal Fungi, Jiangxi Agricultural University, Nanchang 330045, China Nanchang Key Laboratory of Edible and Medicinal Fungi, Jiangxi Agricultural University Nanchang China; 3 Nanchang Key Laboratory of Edible and Medicinal Fungi, Jiangxi Agricultural University, Nanchang 330045, China School of Agricultural Sciences, Jiangxi Agricultural University Nanchang China; 4 Center of Excellence in Fungal Research, Mae Fah Luang University, Chiang Rai 57100, Thailand Center of Excellence in Fungal Research, Mae Fah Luang University Chiang Rai Thailand; 5 School of Science, Mae Fah Luang University, Chiang Rai 57100, Thailand School of Science, Mae Fah Luang University Chiang Rai Thailand; 6 Shenzhen Key Laboratory of Microbial Genetic Engineering, College of Life Science and Oceanography, Shenzhen University, Shenzhen 518060, China Shenzhen Key Laboratory of Microbial Genetic Engineering, College of Life Science and Oceanography, Shenzhen University Shenzhen China; 7 School of Food and Pharmaceutical Engineering, Guizhou Institute of Technology, Guiyang 550025, China School of Food and Pharmaceutical Engineering, Guizhou Institute of Technology Guiyang China

**Keywords:** Homonymy, Index Fungorum registration, replacement name

## Abstract

In Mi et al. (2026; *IMA Fungus* 17: e179140), ten new combinations were proposed in *Funiliomyces*. Subsequent review revealed that certain requirements of the International Code of Nomenclature for algae, fungi, and plants (ICNafp; Turland et al. 2025, Madrid Code) were not fully met. Specifically, for *Funiliomyces biseptatus* (Mi et al. 2026), the basionym indicates that the intended spelling of the epithet should be *F. biseptatus*. However, this spelling would result in a later homonym of *Funiliomyces biseptatus* (Aptroot 2004), the type species of the genus, thereby rendering the name illegitimate. In addition, the newly proposed combinations were not registered in Index Fungorum prior to publication as required under Art. F.5.2, and the identifiers cited corresponded to the basionyms rather than to the new combinations. This corrigendum provides a replacement name, *Funiliomyces biseptatisporus* nom. nov., and presents a corrected, Code-compliant treatment of all nine combinations, including newly issued Index Fungorum identifiers.

## Introduction

The genus *Funiliomyces* was recently revised, and ten new combinations were introduced in Mi et al. (2026) to reflect updated phylogenetic relationships. Following publication, it was brought to our attention that certain nomenclatural requirements of the International Code of Nomenclature for algae, fungi, and plants (ICNafp; Turland et al. 2025) had not been fully met.

Firstly, based on the basionym, the correct spelling of the name *Funiliomyces bisepatus* in Mi et al. (2026) should be *F. biseptatus*. However, this name constitutes a later homonym of *Funiliomyces biseptatus* (Aptroot 2004), the type species of the genus, and is therefore illegitimate under Art. 53.1 of the Code. To resolve this issue, a replacement name (nomen novum) is here proposed.

Secondly, under Art. F.5.2 of the Code, all new fungal names and combinations must be registered in a recognized repository prior to publication. The combinations proposed in the original paper were not registered before publication, and the identifiers cited referred to the basionyms rather than to the newly established combinations.

The purpose of this corrigendum is therefore to provide a fully Code-compliant treatment of the ten taxa in *Funiliomyces*, including the proposal of a replacement name for the illegitimate homonym and the citation of newly issued Index Fungorum identifiers. These nomenclatural amendments do not alter the phylogenetic framework or taxonomic conclusions presented in the original publication.

**Corrected “Title” (p. 1)**:

*Funiliomycetaceae* fam. nov. (*Amphisphaeriales*, *Ascomycota*) accommodating *Funiliomyces*, including *F. jiangxiensis* sp. nov. from *Tetradium ruticarpum*, nine new combinations and one replacement name

**Corrected “Abstract” (p. 1)**:

The diversity of *Funiliomycetaceae* is expanded here by the description of one new species, *Funiliomyces jiangxiensis* sp. nov., nine new combinations (*F. acaciae* comb. nov., *F. calliandrae* comb. nov., *F. fragilis* comb. nov., *F. hwasunensis* comb. nov., *F. mavisleverae* comb. nov., *F. monticola* comb. nov., *F. retrophylli* comb. nov., *F. sparsus* comb. nov., and *F. zapatensis* comb. nov.). and one replacement name (*F. biseptatisporus* nom. nov.).

**Corrected “Table 1” (p. 7)**:

**Table 1.** Taxa, isolate information, and GenBank accession numbers for sequences used in the phylogenetic analyses of *Pezizomycotina*.

**Taxon**	**Strain**	**GenBank accession numbers**	**References**
		**LSU**	
*Acarosporina microspora*	CBS 338.39	AY584643	Lutzoni et al. (2004)
*Amphisphaeria umbrina*	HKUCC 994	AF452029	Jeewon et al. (2003)
*Anungitiomyces stellenboschiensis*	CPC 34726^T^	NG_067874	Crous et al. (2019a)
*Appendicospora hongkongensis*	HKAS 107015	MW240581	Samarakoon et al. (2022)
*Arthonia dispersa*	UPSC 2583	AY571381	Lumbsch et al. (2005)
*Arthrobotrys macroides*	CBS 120.54	MH868799	Vu et al. (2019)
*Arthrobotrys mangrovispora*	MGDW17	EU573355	Swe et al. (2008)
*Ascosphaera apis*	CBS 402.96	FJ358275	Gueidan et al. (2008)
*Aspergillus fumigatus*	INFU/Jc/KF/6	FM179606	Kusari et al. (2009)
*Barrmaelia serenoae*	CPC:37572^T^	MT223876	Crous et al. (2020)
*Caliciopsis pinea*	CBS 139.64	DQ678097	Schoch et al. (2006b)
*Camarops ustulinoides*	AFTOL-ID 72	DQ470941	Spatafora et al. (2006)
*Candelaria concolor*	AFTOL-ID 1706	DQ986791	Miadlikowska et al. (2006)
*Candelariella reflexa*	AFTOL-ID 1271	DQ912331	Miadlikowska et al. (2006)
*Candelariella terrigena*	AFTOL-ID 227	DQ986745	Miadlikowska et al. (2006)
*Candida albicans*	ATCC 18804^T^	NG_054826	Suh et al. (2013)
*Capnodium coffeae*	CBS 147.52	DQ247800	Schoch et al. (2006a)
*Chaenotheca furfuracea*	Wedin 6366 (UPS)^T^	JX000087	Prieto et al. (2013)
*Cirrosporium novae-zelandiae*	CBS 125236	HQ878612	Réblová et al. (2012)
*Cladonia caroliniana*	AFTOL-ID 3	AY584640	Lutzoni et al. (2004)
*Cladosporium herbarum*	CBS 121621	MH874676	Vu et al. (2019)
*Coccomyces strobi*	AFTOL-ID 1250	DQ470975	Spatafora et al. (2006)
*Collemopsidium angermannicum*	s1473	KU556871	Pérez-Ortega et al. (2016)
*Collemopsidium pelvetiae*	RO25	KU556868	Pérez-Ortega et al. (2016)
*Conlarium baiseense*	TD17	MK164655	Xie et al. (2019)
*Cordyceps militaris*	CBS 110.70	MH878247	Vu et al. (2019)
*Corynelia africana*	PREM 57242^T^	NG_058910	Wood et al. (2016)
*Dactylaria acicularis*	CBS 511.72	MH872253	Vu et al. (2019)
*Dactylaria aquatica*	P003	EU107312	GenBank, direct submission
*Dactylaria belliana*	P020	EU107295	GenBank, direct submission
*Dactylaria dimorpha*	P067	EU107305	GenBank, direct submission
*Dactylaria dimorphospora*	CBS 256.70	MH871358	Vu et al. (2019)
*Dactylaria flammulicornuta*	P005	EU107313	GenBank, direct submission
*Dactylaria humicola*	P006	EU107304	GenBank, direct submission
*Dactylaria hyalotunicata*	P010	EU107298	GenBank, direct submission
*Dactylaria longispora*	P001	EU107299	GenBank, direct submission
*Dactylaria lunata*	CBS 689.93	MH874102	Vu et al. (2019)
*Dactylaria parvispora*	P024	EU107296	GenBank, direct submission
*Dactylaria purpurella*	P048	EU107301	GenBank, direct submission
*Dactylaria sahelensis*	CBS 247.93	MH874055	Vu et al. (2019)
*Dactylaria simonensis*	CBS 367.90	MH873904	Vu et al. (2019)
*Dactylaria sporexamorpha*	CBS 690.93	MH874103	Vu et al. (2019)
*Dactylaria uliginicola*	P007	EU107311	GenBank, direct submission
*Dactylellina appendiculata*	CBS 206.64^T^	NG_059071	Li et al. (2006)
*Dactylellina leptospora*	CBS 560.92	AY261162	Crous et al. (2022)
*Dactylospora haliotrepha*	AFTOL-ID 758	FJ176855	Schoch et al. (2009a)
*Dactylospora mangrovei*	AFTOL-ID 2108	FJ176890	Schoch et al. (2009a)
*Delphinella strobiligena*	AFTOL-ID 1257	DQ470977	Spatafora et al. (2006)
*Dendrographa minor*	R. Ornduff 10070	AF279382	Bhattacharya et al. (2000)
*Diatrype disciformis*	AFTOL-ID 927	DQ470964	Spatafora et al. (2006)
*Dothiora cannabinae*	CBS 737.71	DQ470984	Spatafora et al. (2006)
*Elaphomyces granulatus*	AFTOL-ID 436^T^	KT232217	Voglmayr et al. (2019)
*Eremascus albus*	CBS 975.69	MH871279	Voglmayr et al. (2019)
*Exophiala dermatitidis*	AFTOL-ID 668^T^	DQ823100	Voglmayr et al. (2019)
*Funiliomyces acaciae*	CPC 29771^T^	KY173493	Crous et al. (2016)
*Funiliomyces biseptatisporus*	P062	EU107288	GenBank, direct submission
*Funiliomyces calliandrae*	CPC 48004^T^	PV664963	Crous et al. (2025)
*Funiliomyces fragilis*	P057	EU107290	GenBank, direct submission
*Funiliomyces hwasunensis*	CMML 20-35	PQ741487	Liu et al. (2025)
*Funiliomyces jiangxiensis*	CCTCC AF2023034^T^	OQ869216	This study
*Funiliomyces mavisleverae*	BRIP 76362a^T^	PQ431199	Tan and Shivas (2024)
*Funiliomyces monticola*	P060	EU107289	GenBank, direct submission
*Funiliomyces retrophylli*	CBS:148271	ON811548	Crous et al. (2022)
*Funiliomyces sparsus*	P055	EU107291	GenBank, direct submission
*Funiliomyces zapatensis*	P056	EU107287	GenBank, direct submission
*Fusarium graminearum*	CBS 132906	MH877513	Vu et al. (2019)
*Gelasinospora tetrasperma*	CBS 178.33	DQ470980	Spatafora et al. (2006)
*Geoglossum nigritum*	AFTOL-ID 56^T^	AY544650	Schoch et al. (2009b)
*Halosphaeria appendiculata*	CBS 197.60	MH869504	Vu et al. (2019)
*Herpomyces chaetophilus*	D. Haelew. 1097b	MG438352	Haelewaters et al. (2019b)
*Hypocrea lutea*	ATCC 208838	AF543791	Currie et al. (2003)
*Hyponectria buxi*	UME 31430	AY083834	Smith et al. (2003)
*Laboulbenia bruchii*	D. Haelew 1346b	MN394843	Haelewaters et al. (2019a)
*Lecanora contractula*	AFTOL-ID 877	DQ986746	Miadlikowska et al. (2006)
*Lecanora paramerae*	Lumbsch s.n. (F)	EF105422	Crespo et al. (2007)
*Lempholemma polyanthes*	Zoladeski & Lutzoni 11294-L1(2/2) (CANL)	AF356691	Lutzoni et al. (2001)
*Lepraria lobificans*	AFTOL-ID 325	DQ986768	Miadlikowska et al. (2006)
*Lichina confinis*	I1024	MT877184	Garrido-Benavent (2023)
*Lichina pygmaea*	I1028	MT877182	Garrido-Benavent (2023)
*Marcelleina persoonii*	AFTOL-ID 164	DQ470943	Spatafora et al. (2006)
*Microascus longirostris*	CBS 196.61	NG_058479	Daros-Pawlyta and Pawlyta (2023)
*Mycocalicium subtile*	Wedin 6889 (UPS)^T^	AY853379	Wedin et al. (2005)
*Mycosphaerella etlingerae*	CBS 129062	NG_069080	Crous et al. (2011b)
*Myrmecridium schulzeri*	CBS 188.96^T^	EU041829	Arzanlou et al. (2007)
*Nectria cinnabarina*	CBS_114055	KU382228	Zare and Gams (2016)
*Neomyrmecridium asiaticum*	CBS:145080	NG_066291	Crous et al. (2018)
*Nothodactylaria nephrolepidis*	CPC 37028^T^	NG_068668	Crous et al. (2019b)
*Orbilia albidorosea*	CBS 140818^T^	NG_073646	Vu et al. (2019)
*Orbilia vinosa*	AFTOL-ID 905^T^	DQ470952	Spatafora et al. (2006)
*Peltula umbilicata*	AFTOL-ID 891	DQ832334	James et al. (2006)
*Petriella setifera*	AFTOL-ID 956	DQ470969	Spatafora et al. (2006)
*Peziza varia*	JC19031901	MT247057	Van Vooren (2020)
*Peziza vesiculosa*	AFTOL-ID 507	DQ470948	Spatafora et al. (2006)
*Plicaria leiocarpa*	CBS 144.92	DQ842029	Spatafora et al. (2006)
*Pseudodactylaria xanthorrhoeae*	CBS:143414	NG_058521	Crous et al. (2017)
*Pseudotruncatella arezzoensis*	MFLUCC 14-0988^T^	NG_070426	Perera et al. (2018)
*Pyrenula reebiae*	DUKE:0047599^T^	NG_068722	Reeb et al. (2004)
*Ramichloridium anceps*	AFTOL-ID 659^T^	DQ823102	James et al. (2006)
*Roccella fuciformis*	AFTOL-ID 126^T^	AY584654	Lutzoni et al. (2004)
*Roccellographa cretacea*	AFTOL-ID 93	DQ883696	Spatafora et al. (2006)
*Sabahriopsis eucalypti*	CPC:24957	NG_058167	Crous et al. (2015b)
*Saccharomyces cerevisiae*	NRRL Y-12632^T^	NG_042623	Kurtzman and Robnett (2013)
*Sarea difformis*	JR6451^T^	MN938401	Beimforde et al. (2020)
*Sarea resinae*	JR6450	MN938402	Beimforde et al. (2020)
*Schismatomma decolorans*	Ertz 5003 (BR)	AY548815	Lutzoni et al. (2004)
*Sclerophora farinacea*	Wedin 6414 (UPS)	JX000095	Prieto et al. (2013)
*Sordaria fimicola*	SMH4106	AY780079	Miller and Huhndorf (2005)
*Spathularia velutipes*	AFTOL-ID 1291	FJ997861	Voglmayr et al. (2019)
*Sphinctrina turbinata*	AFTOL-ID 1721	EF413632	Geiser et al. (2006)
*Teloschistes exilis*	AFTOL-ID 87	AY584647	Lutzoni et al. (2004)
*Toxicocladosporium chlamydosporum*	CBS 124157^T^	NG_069916	Vu et al. (2019)
*Trapelia placodioides*	KS163	KU844623	Schneider et al. (2016)
*Trichocoma paradoxa*	CBS 788.83	FJ358290	Gueidan et al. (2008)
*Trichoglossum hirsutum*	AFTOL-ID 64	AY544653	Voglmayr et al. (2019)
*Trinosporium guianense*	CBS 132537^T^	NG_042682	Crous et al. (2012)
*Tryblidiopsis pinastri*	AFTOL-ID 1319	DQ470983	Spatafora et al. (2006)
*Umbilicaria arctica*	AFTOL-ID 1266	DQ986772	Miadlikowska et al. (2006)
*Umbilicaria hyperborea*	OSC:McCune 35477	MH290350	McCune (2018)
*Volutella ciliata*	CBS 127312	MH875955	Vu et al. (2019)
*Xylaria acuta*	AFTOL-ID 63	AY544676	Hongsanan and Hyde (2017)
*Xylaria hypoxylon*	AFTOL-ID 51	AY544648	Voglmayr et al. (2019)
*Xylobotryum andinum*	XA1	MH468791	Voglmayr et al. (2019)
*Xylobotryum portentosum*	XP	MH468792	Voglmayr et al. (2019)
*Xylona heveae*	CBS 132557^T^	NG_066160	Gazis et al. (2012)

Notes: New sequences determined for this study and taxonomic novelties are given in Light orange. New combinations are given in blue. Superscript T denotes ex-type strains.

In Table 1, *Funiliomyces bisepatus* has been corrected to *Funiliomyces biseptatisporus*.

**Corrected “Table 2” (p. 9)**:

**Table 2.** Taxa, isolate information, and GenBank accession numbers for sequences used in the phylogenetic analyses of *Amphisphaeriales*.

**Taxon**	**Strain**	**GenBank accession numbers**			**References**
		**LSU**	**ITS**	**RPB2**	
*Achaetomium macrosporum*	CBS 532.94	KX976699	KX976574	KX976797	Wang et al. (2016)
*Amphisphaeria fuckelii*	CBS:140409^T^	KT949902	KT949902	MH554918	Jaklitsch et al. (2016)
*Amphisphaeria thailandica*	MFLU 18-0794^T^	MH971235	MH971225	MK033640	Samarakoon et al. (2019)
*Anungitiomyces stellenboschiensis*	CPC:34726^T^	MK876415	MK876376	NA	Crous et al. (2019b)
*Appendicospora hongkongensis*	HKAS 107015	MW240581	MW240651	MW658638	Samarakoon et al. (2022)
*Arthrinium hysterinum*	ICMP 6889	MK014841	MK014874	DQ368649	Pintos et al. (2019)
*Arthrinium pseudoparenchymaticum*	SICAUCC 18-0008	MK346321	MK346319	MK359207	Yang et al. (2019)
*Beltrania pseudorhombica*	CBS:138003^T^	KJ869215	MH554124	MH555032	Crous et al. (2014a)
*Beltraniopsis neolitseae*	CBS:137974^T^	KJ869183	KJ869126	NA	Crous et al. (2014a)
*Brachiampulla verticillata*	ICMP 15993	MW144403	MW144419	NA	Réblová et al. (2021)
*Castanediella acaciae*	CBS 139896^T^	MH878661	KR476728	NA	Vu et al. (2019)
*Castanediella cagnizarii*	MUCL 41095	KC775707	KC775732	NA	Becerra-Hernández et al. (2016)
*Castanediella ramosa*	MUCL 39857	KC775711	KC775736	NA	Becerra-Hernández et al. (2016)
*Chaetomium elatum*	CBS 374.66	MH870466	KC109758	KF001820	Vu et al. (2019)
*Clypeophysalospora latitans*	CBS 141463^T^	KX820261	KX820250	NA	Giraldo et al. (2017)
*Cylindrium elongatum*	CBS 115974	KM231733	KM231853	KM232429	Lombard et al. (2015)
*Cylindrium grande*	CBS:145578	MK876426	MK876385	MK876482	Crous et al. (2019a)
*Funiliomyces acaciae*	CPC 29771	KY173493	KY173400	NA	Crous et al. (2016)
*Funiliomyces biseptatisporus*	P062	EU107288	NA	NA	GenBank, direct submission
*Funiliomyces biseptatus*	CBS 100373^T^	NG_067443	NR_159862	NA	Aptroot (2004)
*Funiliomyces calliandrae*	CPC 48004	PV664963	PV664937	PV664022	Crous et al. (2025)
*Funiliomyces fragilis*	P057	EU107290	NA	NA	GenBank, direct submission
*Funiliomyces hwasunensis*	CMML 20-35	PQ741487	PQ741486	NA	Liu et al. (2025)
*Funiliomyces hwasunensis*	CMML 20-88	PQ741488	NA	NA	Liu et al. (2025)
*Funiliomyces jiangxiensis*	CCTCC AF2023034^T^	OQ869216	OQ869213	OR046688	This study
*Funiliomyces jiangxiensis*	CCTCC AF2023033	OQ869214	OQ869215	OR046687	This study
*Funiliomyces mavisleverae*	BRIP 76362a	PQ431199	PQ431206	NA	Tan and Shivas (2024)
*Funiliomyces monticola*	P060	EU107289	NA	NA	GenBank, direct submission
*Funiliomyces retrophylli*	CBS:148271	ON811548	ON811489	NA	Crous et al. (2022)
*Funiliomyces sparsus*	P055	EU107291	NA	NA	GenBank, direct submission
*Funiliomyces zapatensis*	P056	EU107287	NA	NA	GenBank, direct submission
*Hyponectria buxi*	UME 31430	AY083834	NA	NA	Smith et al. (2003)
*Iodosphaeria honghensis*	MFLU 19-0719^T^	MK722172	MK737501	MK791287	Marasinghe et al. (2019)
*Iodosphaeria tongrenensis*	MFLU:15-0393	KR095283	KR095282	NA	Li et al. (2015)
*Leiosphaerella praeclara*	CBS:125586	JF440976	JF440976	NA	Jaklitsch and Voglmayr (2012)
*Melogramma campylosporum*	MFLU 17-0348	MW240575	MW240645	MW658632	Samarakoon et al. (2022)
*Melogramma campylosporum*	MFLU 18-0778	MW240576	MW240646	MW658633	Samarakoon et al. (2022)
*Neoamphisphaeria hyalinospora*	MFLU 19-2131^T^	MW240579	MW240649	MW658636	Samarakoon et al. (2022)
*Neoamphisphaeria shangrilaensis*	HKAS 130274	PP584800	PP584703	NA	Dissanayake et al. (2024)
*Neophysalospora eucalypti*	CBS:138864^T^	KP004490	KP004462	NA	Crous et al. (2014b)
*Nothodactylaria comitabilis*	CPC 45173	PQ498974	PQ498925	NA	Crous et al. (2024)
*Nothodactylaria fusiformis*	KUNCC:23-13961^T^	PQ671162	PQ671242	PQ662509	Zhang et al. (2025)
*Nothodactylaria guizhouensis*	KUNCC:23-14080^T^	PQ671163	PQ671243	PQ662510	Zhang et al. (2025)
*Nothodactylaria nephrolepidis*	CPC:37028	MN567639	MN562132	MN556809	Crous et al. (2019b)
*Nothodactylaria nephrolepidis*	CBS:146078^T^	NG_068668	NR_166331	NA	Crous et al. (2019b)
*Nothodactylaria polyblastis*	KUNCC:23-13922^T^	PQ671164	PQ671244	PQ662511	Zhang et al. (2025)
*Nothodactylaria woodwardiae*	KUNCC:23-13927^T^	PQ671165	PQ671245	PQ662512	Zhang et al. (2025)
*Nothodactylaria woodwardiae*	KUNCC:23-13886	PQ671166	PQ671246	PQ662513	Zhang et al. (2025)
*Nothodactylaria woodwardiae*	KUNCC:23-14045	PQ671167	PQ671247	PQ662514	Zhang et al. (2025)
*Nothodactylaria woodwardiae*	KUNCC:23-13954	PQ671168	PQ671248	NA	Zhang et al. (2025)
*Nothodactylaria woodwardiae*	KUNCC23-14006	PQ671169	PQ671249	NA	Zhang et al. (2025)
*Oxydothis metroxyli*	MFLUCC15-0283	KY206764	KY206775	NA	Konta et al. (2016)
*Oxydothis metroxylonicola*	MFLUCC 15-0281^T^	KY206763	KY206774	KY206781	Konta et al. (2016)
*Oxydothis palmicola*	MFLUCC 15-0806^T^	KY206765	KY206776	KY206782	Konta et al. (2016)
*Phlogicylindrium eucalypti*	CBS 120080^T^	DQ923534	DQ923534	MH554893	Summerell et al. (2006)
*Phlogicylindrium uniforme*	CBS 131312^T^	JQ044445	JQ044426	NA	Crous et al. (2011a)
*Polyscytalum eucalyptorum*	CPC 17207^T^	KJ869176	KJ869118	NA	Crous et al. (2014a)
*Pseudapiospora corni*	WU:36852^T^	KT949907	KT949907	NA	Jaklitsch et al. (2016)
*Pseudomassaria chondrospora*	CBS:125600	JF440981	JF440981	NA	Jaklitsch and Voglmayr (2012)
*Pseudosporidesmium knawiae*	CBS:123529^T^	MH874823	MH863299	NA	Vu et al. (2019)
*Pseudosporidesmium lambertiae*	CBS:143169^T^	MG386087	MG386034	NA	Crous et al. (2017)
*Pseudotruncatella arezzoensis*	MFLUCC:14-0988^T^	MG192317	MG192320	NA	Perera et al. (2018)
*Pseudotruncatella bolusanthi*	CBS:145532^T^	MK876448	MK876407	NA	Crous et al. (2019a)
*Robillarda sessilis*	CBS 114312^T^	KR873284	KR873256	NA	Crous et al. (2015a)
*Seiridium marginatum*	CBS:140403^T^	KT949914	KT949914	MK523301	Jaklitsch et al. (2016)
*Sordaria fimicola*	CBS 723.96	NA	MH862606	NA	Vu et al. (2019)
*Strelitziomyces knysnanus*	CPC:37067^T^	MN567642	MN562135	MN556810	Crous et al. (2019b)
*Subsessila turbinata*	MFLUCC:15-0831^T^	KX762289	KX762288	NA	Lin et al. (2017)
*Vialaea insculpta*	DAOM:240257	JX139726	JX139726	NA	Shoemaker et al. (2013)
*Vialaea minutella*	BRIP:56959	KC181924	KC181926	NA	McTaggart et al. (2013)
*Xyladictyochaeta lusitanica*	CBS:142290^T^	KY853543	KY853479	NA	Hernández-Restrepo et al. (2017)

Notes: New sequences determined for this study and taxonomic novelties are given in light orange. New combinations are given in blue. Superscript T denotes ex-type strains. NA indicates the unavailability of data.

In Table 2, *Funiliomyces bisepatus* has been corrected to *Funiliomyces biseptatisporus*.

**Corrected “Figure 1” (p. 12)**:

**Figure 1.**
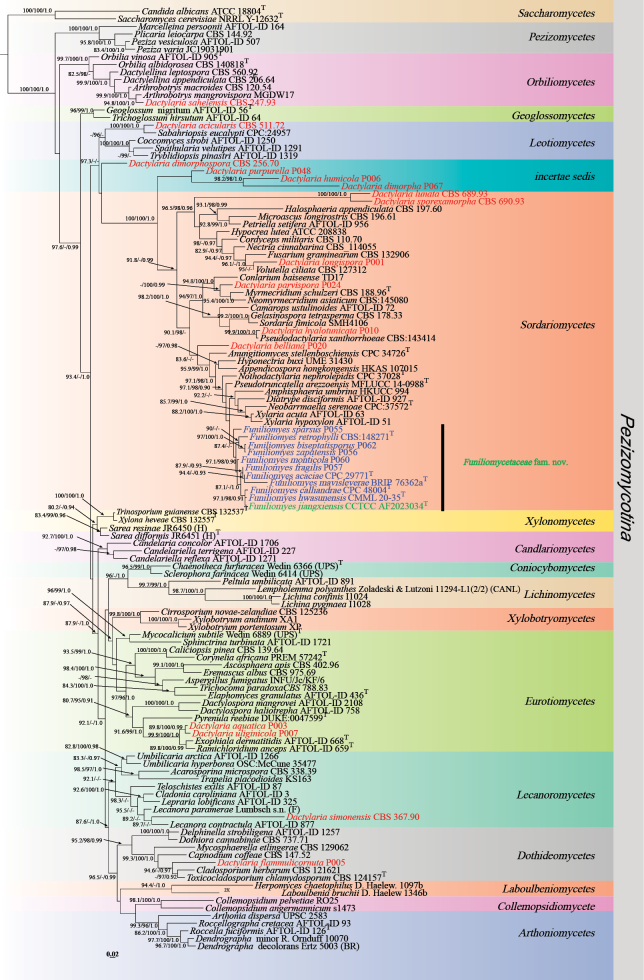
Maximum likelihood phylogenetic tree of *Pezizomycotina* based on LSU sequences, with Shimodaira-Hasegawa-like approximate likelihood ratio test (SH-aLRT) (left), ultrafast bootstrap (UFB) (middle), and Bayesian posterior probabilities (BPP) values (right) near the corresponding node. Only one of SH-aLRT > 80 or UFB > 95 for ML and BYPP > 0.90 for BI is indicated along the branches (SH-aLRT/UFB/BPP). The “*Dactylaria*” species are indicated in red, the newly generated sequences are indicated in green, and species for reclassification from *Dactylaria to Funiliomyces* are in blue. Ex-type strains are marked with T after the strain number. The ML phylogram is available in TreeBASE (study accession S32460; http://purl.org/phylo/treebase/phylows/study/TB2:S32460).

In Figure 1, *Funiliomyces bisepatus* has been corrected to *Funiliomyces biseptatisporus*.

**Corrected “Figure 2” (p. 14)**:

**Figure 2.**
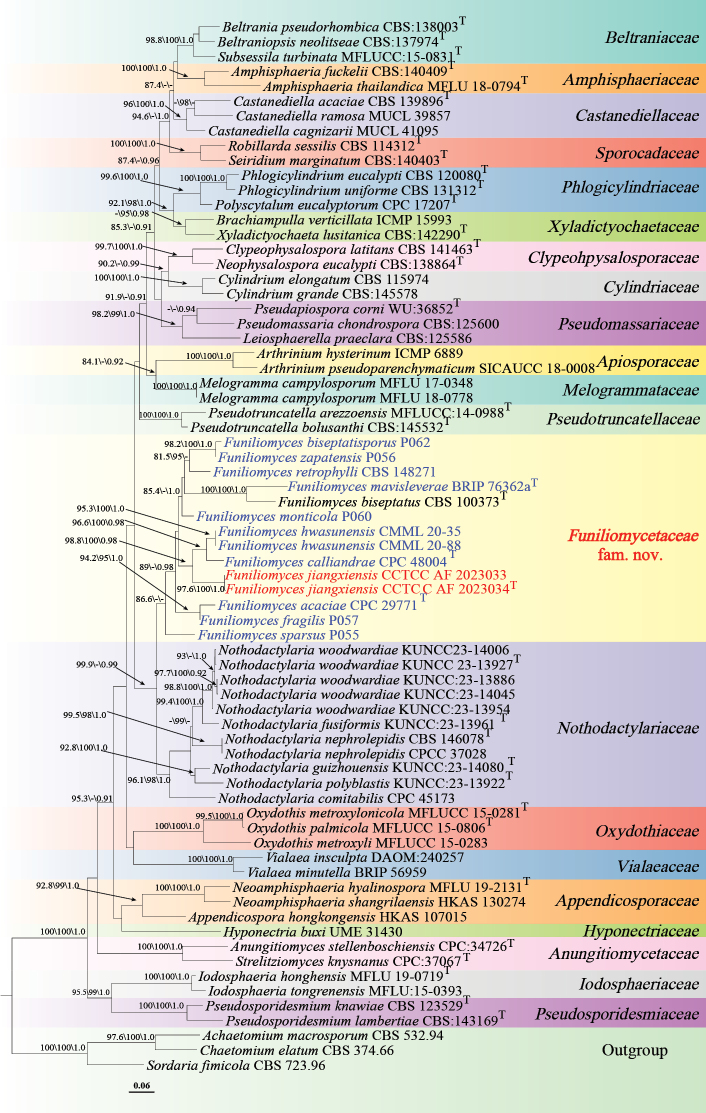
Maximum likelihood phylogenetic tree of *Amphisphaeriales* based on combined LSU, ITS, and RPB2 sequences, with Shimodaira-Hasegawa-like approximate likelihood ratio test (SH-aLRT) (left), ultrafast bootstrap (UFB) (middle), and Bayesian posterior probabilities (BPP) values (right) near the corresponding node. Only one of SH-aLRT > 80 or UFB > 95 for ML and BYPP > 0.90 for BI is indicated along the branches (SH-aLRT/UFB/BPP). Ex-type strains are marked with T after the strain number. The newly generated sequences are indicated in red and species for reclassification are in blue. The ML phylogram is available in TreeBASE (study accession S32462; http://purl.org/phylo/treebase/phylows/study/TB2:S32462).

In Figure 2, *Funiliomyces bisepatus* has been corrected to *Funiliomyces biseptatisporus*.

## Corrected “Taxonomy” (pp. 20–22):

The name *Funiliomyces bisepatus* (Mi et al. 2026) was proposed based on a basionym for which the correct spelling of the epithet should be *F. biseptatus*. Consequently, the name would constitute a later homonym of *Funiliomyces biseptatus* (Aptroot 2004), the type species of the genus, and is therefore illegitimate. In accordance with the Code, we here propose a replacement name:

### Funiliomyces biseptatisporus

Taxon classification

Animalia



Amphisphaeriales



Amphisphaeriaceae



L.X. Mi, H.Y. Song, D.M. Hu & K.D. Hyde nom. nov.

EB227A60-C2EF-50FA-810F-8D42C6A2FE3C

Index Fungorum: IF905139

#### Etymology.

The epithet biseptatisporus refers to the two-septate spore (Latin *bi-* = two; *septati* = septate; *sporus* = spore).

#### Replaced synonym.

*Dactylaria biseptata* Matsush., Icones Microfungorum a Matsushima lectorum: 48 (1975), non *Funiliomyces biseptatus*Aptroot (2004).

#### Holotype.

MFC-4029.

#### Type information.

Japan, Ohdaigahara, Nara Pref, on a rotten leaf of *Rhododendron metternichii* (*Ericaceae*), July 1970, MFC-4029 (holotype).

#### Description.

See the original description in Matsushima (1975) on page 48–49.

##### Other new combination species in *Funiliomyces* were registered in Index Fungorum:

### 1. Funiliomyces acaciae

Taxon classification

Animalia



Amphisphaeriales



Amphisphaeriaceae



(Crous) L.X. Mi, H.Y. Song, D.M. Hu & K.D. Hyde comb. nov.

E564D8CF-3E75-5DE6-B27D-382B98D43D7F

Index Fungorum: IF905125

#### Basionym.

*Dactylaria acaciae* Crous, Persoonia 37: 321 (2016).

#### Holotype.

CBS H-22876.

#### Type information.

USA, Hawaii, Oahu, on leaves of *Acacia koa* (*Fabaceae*), 30 September 2015, J.J. Le Roux (holotype CBS H-22876, culture ex-type CPC 29771 = CBS 142087).

#### Description.

See the original description in Crous et al. (2016) on page 321.

### 2. Funiliomyces calliandrae

Taxon classification

Animalia



Amphisphaeriales



Amphisphaeriaceae



(Crous) L.X. Mi, H.Y. Song, D.M. Hu & K.D. Hyde comb. nov.

AC5C33D7-DF29-53C9-BD05-15AE3CA8DFED

Index Fungorum: IF905126

#### Basionym.

*Dactylaria calliandrae* Crous, Persoonia 54: 376–377 (2025).

#### Holotype.

CBS H-25715.

#### Type information.

Brazil, Minas Gerais, Viçosa, Clonar nursery, on living leaf of *Calliandra tweediei* (*Fabaceae*), 25 February, 2024, P.W. Crous, HPC 4399 (holotype CBS H-25715; culture ex-type COAD 3994 = CPC 48004).

#### Description.

See the original description in Crous et al. (2025) on page 376–377.

### 3. Funiliomyces fragilis

Taxon classification

Animalia



Amphisphaeriales



Amphisphaeriaceae



(de Hoog) L.X. Mi, H.Y. Song, D.M. Hu & K.D. Hyde comb. nov.

0BB13043-8328-5EB1-8C57-17E16E750F76

Index Fungorum: IF905132

#### Basionym.

*Dactylaria fragilis* de Hoog, Studies in Mycology 26: 30 (1985).

#### Holotype.

No.6074(CBS).

#### Type information.

The Netherlands, Opsterland, Oldeterp, on cupules of *Fagus sylvatica* (*Fagaceae*), H.A. van der Aa, October, 1977.

#### Description.

See the original description in de Hoog and van Oorschot (1985) on page 30.

### 4. Funiliomyces hwasunensis

Taxon classification

Animalia



Amphisphaeriales



Amphisphaeriaceae



(H.F. Liu & H.K. Sang) L.X. Mi, H.Y. Song, D.M. Hu & K.D. Hyde comb. nov.

B6B5283C-4885-588C-982E-18018DE012F3

Index Fungorum: IF905133

#### Basionym.

*Dactylaria hwasunensis* H.F. Liu & H.K. Sang, IMA Fungus 16(e138479): 10 (2025).

#### Holotype.

CMML 20-35H.

#### Type information.

Korea, South Jeolla Province, Hwasun, isolated from roots of *Zoysia japonica* (*Poaceae*), October 2020, H. Liu & H. Sang, holotype CMML 20-35H, ex-holotype CMML 20-35, ex-isotype CMML 20-88.

#### Description.

See the original description in Liu et al. (2025) on page 10–14.

### 5. Funiliomyces mavisleverae

Taxon classification

Animalia



Amphisphaeriales



Amphisphaeriaceae



(Y.P. Tan, Bishop-Hurley & Marney) L.X. Mi, H.Y. Song, D.M. Hu & K.D. Hyde comb. nov.

E54B6830-154F-5DA2-B72E-E0D3E25312DE

Index Fungorum: IF905134

#### Basionym.

*Dactylaria mavisleverae* Y.P. Tan, Bishop-Hurley & Marney, Index of Australian Fungi 46: 3 (2024).

#### Holotype.

BRIP 76362a.

#### Type information.

Australia, Queensland, Brisbane, phylloplane of unidentified ornamental plant, January, 2024, T.S. Marney, BRIP 76362a (holotype).

#### Description.

See the original description in Tan and Shivas (2024) on page 3.

### 6. Funiliomyces monticola

Taxon classification

Animalia



Amphisphaeriales



Amphisphaeriaceae



(R.F. Castañeda & W.B. Kendr.) L.X. Mi, H.Y. Song, D.M. Hu & K.D. Hyde comb. nov.

E48F7902-6A21-5E7D-882B-B12108163958

Index Fungorum: IF905135

#### Basionym.

*Dactylaria monticola* R.F. Castañeda & W.B. Kendr., University of Waterloo Biology Series, 35: 30 (1991).

#### Holotype.

INIFAT C 91/82.

#### Type information.

Cuba, Granma, Buey Arriba, La Estrella, on dead leaves of *Andira inermis* (*Leguminosae*), R.F. Castañeda Ruíz, 14 March 1991.

#### Description.

See the original description in Castañeda Ruíz and Kendrick (1991) on page 30.

### 7. Funiliomyces retrophylli

Taxon classification

Animalia



Amphisphaeriales



Amphisphaeriaceae



(Crous) L.X. Mi, H.Y. Song, D.M. Hu & K.D. Hyde comb. nov.

48274337-7E1B-5868-B562-682E8B96A454

Index Fungorum: IF905136

#### Basionym.

*Dactylaria retrophylli* Crous, Fungal Systematics and Evolution 10: 41 (2022).

#### Holotype.

HPC 3260.

#### Type information.

Colombia, Finca El Cedral, on leaves of *Retrophyllum rospigliosii* (*Podocarpaceae*), M.J. Wingfield, February, 2020, HPC 3260 (holotype CBS H-24817, culture ex-type CPC 39510 = CBS 148271).

#### Description.

See the original description in Crous et al. (2022) on page 41–42.

### 8. Funiliomyces sparsus

Taxon classification

Animalia



Amphisphaeriales



Amphisphaeriaceae



(R.F. Castañeda & W.B. Kendr.) L.X. Mi, H.Y. Song, D.M. Hu & K.D. Hyde comb. nov.

D1921547-3059-5D2E-ADC9-503C3FF092A8

Index Fungorum : IF905137

#### Basionym.

*Dactylaria sparsa* R.F. Castañeda & W.B. Kendr., University of Waterloo Biology Series. 35:33 (1991).

#### Holotype.

INIFAT C 91/68-2.

#### Type information.

Cuba, C. Habana, Santiago de las Vegas, on decaying leaves, R.F. Castañeda Ruíz, 18 February 1990.

#### Description.

See the original description in Castañeda Ruíz and Kendrick (1991) on page 33.

### 9. Funiliomyces zapatensis

Taxon classification

Animalia



Amphisphaeriales



Amphisphaeriaceae



(R.F. Castañeda) L.X. Mi, H.Y. Song, D.M. Hu & K.D. Hyde comb. nov.

BF770A3C-2A2E-5228-808A-630CFB272414

Index Fungorum: IF905138

#### Basionym.

*Dactylaria zapatensis* R.F. Castañeda, Fungi Cubenses III (La Habana): 5 (1988)

#### Holotype.

INIFAT C85/98

#### Type information.

Cuba, Matanzas, Ciénaga de Zapata, on fallen leaves of *Nectandra coriacea* (*Lauraceae*), R.F. Castañeda Ruíz, 26 May 1985.

#### Description.

See the original description in Castañeda Ruíz (1988) on page 5.

##### Corrected “Table 4” (p.25):

In Table 4, *F. bisepatus* has been corrected to *F. biseptatisporus*.

**Table 4.** Asexual morphological features, lifestyle, host associations, and distribution of *Funiliomyces* species.

**Species**	**Mycelium**	**Conidiophores**	**Conidiogenous cel**ls	**Conidi**a		**Life style**	**Host**	**Country**	**References**
				**Shape/colour**	**Size**				
*Funiliomyces acaciae*	2–2.5 µm, hyaline	7–60 × 2–3.5 µm, brown, 0–7-septate	7–25 × 2–3.5 µm, brown, with flat-tipped denticles (0.5–1.5 × 0.5 µm)	narrowly fusoid ellipsoid, 2-septate, hyaline	(16–)25– 34(–37) × 2(–2.5) µm	epiphytic	*Acacia koa* (*Fabaceae*)	USA	Crous et al. (2016)
*F. biseptatisporus*	1–3 µm, hyaline to moderately brown	5–20 µm × 3–3.5 µm, moderately brown, cylindrical, 0–2-septata	cylindrical, moderately brown, with successive denticles and geniculate structure	cylindrical, 2-septata, individually, hyaline to pale smoky	(22–) 27–33 (–35) × 1.5–2 µm	saprobic	*Rhododendron metternichii* (*Ericaceae*)	Japan	Matsushima (1975)
*F. biseptatus*	–	–	–	–	–	saprobic	Undefined (*Bromeliaceae*)	Brazil	Aptroot (2004)
*F. calliandrae*	2–3 µm, hyphae	hyaline (appearing subhyaline with age), mostly reduced to conidiogenous cells	10–25 × 3–4 µm hyaline, prominent cylindrical denticles, 1–3 × 1.5 µm	spindle-shaped, apex subobtuse, base truncate, (3–)5–6(–8)-septate, hyaline	(37–)40–45(–47) × (2.5–)3 µm	epiphytic	*Calliandra tweediei* (*Fabaceae*)	Brazil	Crous et al. (2025)
*F. fragilis*	pale brown	15–30 × 4 μm at the base, 0–3 thin septa, subhyaline to pale brown	thin-walled, hyaline, slightly lobed; denticles absent; rhexolytic secession with inconspicuous scars	clavate, 2-septate, hyaline	18–26 × 1.5, base 0.6 µm wide	saprobic	*Fagus sylvatica* (*Fagaceae*)	Netherlands	de Hoog and van Oorscho (1985)
*F. hwasunensis*	–	6–35 × 2.2–2.8 µm， hyaline, aseptate or septate	2–2.8 μm wide，terminal, integrated, hyaline	clavate, blunt end，hyaline, 1–5 septate	10–60 × 2.2–2.8 μm	endophytic	*Zoysia japonica* (*Poaceae*)	Korea	Liu et al. (2025)
*F. jiangxiensis*	1.7–2.6 µm, hyaline	5–37 × 2–4 µm, hyaline, septate, sometimes reduced to conidiogenous cells	4–18 × 1–4 µm, with conspicuous, cylindrical denticles, up to 0.9 µm wide	narrowly fusoid-ellipsoid, guttulate, 0–3-septate, hyaline	20–40 × 1.5–4 µm	endophytic	*Tetradium ruticarpum* (*Rutaceae*)	China	This study
*F. mavisleverae*	–	–	–	–	–	epiphytic	an unidentified ornamental plant	Australia	Tan and Shivas (2024)
*F. monticola*	1–1.5μm, colourless	15–40 × 2–2.5 μm, colourless, septate	12–17× 2–3 µm, with conspicuous, truncate denticles in the apical region	fusiform, 1-septate, colourless	30–37 × 1–1.5 µm	saprobic	*Andira inermis* (*Leguminosae*)	Cuba	Castañeda Ruíz and Kendrick (1991)
* retrophylli *	1.5–2.5 μm, hyaline	conidiophores reduced to conidiogenous cells or with supporting cell	with 1–2 × 1 μm apex denticulate	medianly 1-septate, straight to narrowly fusoid, 6–20 × 2.5–3.5	(26–)30–33(–37) × (1.5–)2 µm	epiphytic	*Retrophyllum rospigliosii* (*Podocarpaceae*)	Colombia	Crous et al. (2022)
*F. sparsus*	1–1.5 μm, pale brown	0–1-septate, pale brown uasually reduced to a conidiogenous cell.	7–12 × 3–4 µm, pale brown or almost colourless, with large, conspicuous, truncate denticles, 1.5–2 μm	subcylindrical, (2–)3-septated with a false septum near each end, colouress or almost colourless	26–36 × 1.5–2 µm	saprobic	decaying leaves (unidentified)	Cuba	Castañeda Ruíz and Kendrick (1991)
* zapatensis *	1–1.5 μm, light brown	12–60 × 1–2 µm, septate, light brown, up to 24 µm wide at apex	polyblastic, denticulate, sympodial, inflated at apex	cylindrical, 2-septata, hyaline, septa visible near extremities	18–26 × 1–1.5 µm	saprobic	*Nectandra coriacea* (*Lauraceae*)	Cuba	Castañeda Ruíz (1988)

Notes: The symbol “–” denotes no information available.

**Corrected “Discussion” (p.26)**:

Accordingly, we transfer the ten previously described “*Dactylaria*” species into the genus *Funiliomyces* including nine new combinations (*F. acaciae*, *F. calliandrae*, *F. fragilis*, *F. hwasunensis*, *F. mavisleverae*, *F. monticola*, *F. retrophylli*, *F. sparsus*, and *F. zapatensis*) and one replacement name (nomen novum), *F. biseptatisporus*.

## Supplementary Material

XML Treatment for Funiliomyces biseptatisporus

XML Treatment for Funiliomyces acaciae

XML Treatment for Funiliomyces calliandrae

XML Treatment for Funiliomyces fragilis

XML Treatment for Funiliomyces hwasunensis

XML Treatment for Funiliomyces mavisleverae

XML Treatment for Funiliomyces monticola

XML Treatment for Funiliomyces retrophylli

XML Treatment for Funiliomyces sparsus

XML Treatment for Funiliomyces zapatensis

## Additional information

### Artificial Intelligence (AI) use

The authors accept full responsibility for the content of the manuscript, including the disclosure of any use of AI.

No AI tools were used in the preparation of this manuscript.

### Adherence to national and international regulations

All the fungal strains used in this study have been legally obtained, respecting the Convention on Biological Diversity (Rio Convention).

### Author ORCIDs

Li-Xue Mi https://orcid.org/0009-0009-0691-7171

Dian-Ming Hu https://orcid.org/0000-0002-4750-2871

Kevin D. Hyde https://orcid.org/0000-0002-2191-0762

Prapassorn D. Eungwanichayapant https://orcid.org/0000-0001-8005-4137

Ausana Mapook https://orcid.org/0000-0001-7929-2429

Danushka S. Tennakoon https://orcid.org/0000-0003-2306-1255

Jing-Yi Zhang https://orcid.org/0000-0003-0606-6169

Hai-Yan Song https://orcid.org/0009-0001-4140-5720

### Data availability

All of the data that support the findings of this study are available in the main text.

## References

[B1] Aptroot A (2004) Two new ascomycetes with long gelatinous appendages collected from monocots in the tropics. Studies in Mycology 50: 1–27.

[B2] Arzanlou M, Groenewald JZ, Gams W et al (2007) Phylogenetic and morphotaxonomic revision of *Ramichloridium* and allied genera. Studies in Mycology 58: 57–93. 10.3114/sim.2007.58.03 PMC2104745 18490996

[B3] Becerra-Hernández CI, González D, De Luna E et al. (2016) First report of pleoanamorphy in *Gyrothrix verticiclada* with an Idriella-like synanamorph. Cryptogamie, Mycologie 37(2): 241–252. 10.7872/crym/v37.iss2.2016.241

[B4] Beimforde C, Schmidt AR, Rikkinen J et al. (2020) *Sareomycetes* cl. nov.: A new proposal for placement of the resinicolous genus *Sarea* (*Ascomycota*, *Pezizomycotina*). Fungal Systematics and Evolution 6: 25–37. 10.3114/fuse.2020.06.02 PMC7451776 32904095

[B5] Bhattacharya D, Lutzoni F, Reeb V et al. (2000) Widespread occurrence of spliceosomal introns in the rDNA genes of ascomycetes. Molecular Biology and Evolution 17(12): 1971–1984. 10.1093/oxfordjournals.molbev.a026298 11110913

[B6] Castañeda Ruíz RF (1988) Fungi cubenses III. Instituto de Investigaciones *Fundamentales* en Agricultura Tropical “Alejandro de Humboldt”, 1–27. http://www.cybertruffle.org.uk/cyberliber/03416/cfo_.htm

[B7] Castañeda Ruíz RF, Kendrick B (1991) Ninety-nine conidial fungi from Cuba and three from Canada. University of Waterloo Biology Series 35: 1–132.

[B8] Crespo A, Lumbsch HT, Mattsson JE et al. (2007) Testing morphology-based hypotheses of phylogenetic relationships in *Parmeliaceae (Ascomycota)* using three ribosomal markers and the nuclear RPB1 gene. Molecular Phylogenetics and Evolution 44(2): 812–824. 10.1016/j.ympev.2006.11.029 17276700

[B9] Currie CR, Wong B, Stuart AE et al. (2003) Ancient tripartite coevolution in the attine ant-microbe symbiosis. Science 299(5605): 386–388. 10.1126/science.1078155 12532015

[B10] Crous PW, Begoude BAD, Boers J et al. (2022) New and Interesting Fungi. 5. Fungal Systematics and Evolution 10: 19–90. 10.3114/fuse.2022.10.02 PMC9903348 36789279

[B11] Crous PW, Carnegie AJ, Wingfield MJ et al. (2019a) Fungal Planet description sheets: 868–950. Persoonia 42: 291–473. 10.3767/persoonia.2019.42.11 PMC6712538 31551622

[B12] Crous PW, Carris LM, Giraldo A et al. (2015a) The Genera of Fungi - fixing the application of the type species of generic names - G 2: *Allantophomopsis*, *Latorua*, *Macrodiplodiopsis*, *Macrohilum*, *Milospium*, *Protostegia*, *Pyricularia*, *Robillarda*, *Rotula*, *Septoriella*, *Torula*, and *Wojnowicia*. IMA Fungus 6(1): 163–198. 10.5598/imafungus.2015.06.01.11 PMC4500082 26203422

[B13] Crous PW, Catcheside DEA, Catcheside PS et al. (2025) Fungal Planet description sheets: 1781–1866. Persoonia 54: 327–587. 10.3114/persoonia.2025.54.10 PMC12308287 40746709

[B14] Crous PW, Luangsa-Ard JJ, Wingfield MJ et al. (2018) Fungal Planet description sheets: 785–867. Persoonia 41: 238–417. 10.3767/persoonia.2018.41.12 PMC6344811 30728607

[B15] Crous PW, Shivas RG, Quaedvlieg W et al. (2014a) Fungal Planet description sheets: 214–280. Persoonia 32: 184–306. 10.3767/003158514X682395 PMC4150077 25264390

[B16] Crous PW, Summerell BA, Shivas RG et al. (2011a) Fungal Planet description sheets: 92–106. Persoonia 27: 130–162. 10.3767/003158511X617561 PMC3251320 22403481

[B17] Crous PW, Summerell BA, Shivas RG et al. (2012) Fungal Planet description sheets: 107–127. Persoonia 28: 138–182. 10.3767/003158512X652633 PMC3409410 23105159

[B18] Crous PW, Tanaka K, Summerell BA et al. (2011b) Additions to the Mycosphaerella complex. IMA Fungus 2(1): 49–64. 10.5598/imafungus.2011.02.01.08 PMC3317358 22679588

[B19] Crous PW, Wingfield MJ, Burgess TI et al. (2016) Fungal Planet description sheets: 469–557. Persoonia 37: 218–403. 10.3767/003158516X694499 PMC5315290 28232766

[B20] Crous PW, Wingfield MJ, Burgess TI et al. (2017) Fungal Planet description sheets: 625–715. Persoonia 39: 270–467. 10.3767/persoonia.2017.39.11 PMC5832955 29503478

[B21] Crous PW, Wingfield MJ, Guarro J et al. (2015b) Fungal Planet description sheets: 320–370. Persoonia 34: 167–266. 10.3767/003158515X688433 PMC4510277 26240451

[B22] Crous PW, Wingfield MJ, Jurjević Ž et al. (2024) Fungal Planet description sheets: 1697–1780. Fungal Syst Evol 14: 325–577. 10.3114/fuse.2024.14.19 PMC11736264 39830292

[B23] Crous PW, Wingfield MJ, Lombard L et al. (2019b) Fungal Planet description sheets: 951–1041. Persoonia 43: 223–425. 10.3767/persoonia.2019.43.06 PMC7085856 32214501

[B24] Crous PW, Wingfield MJ, Schumacher RK et al. (2014b) Fungal Planet description sheets: 281–319. Persoonia 33: 212–289. 10.3767/003158514X685680 PMC4312934 25737601

[B25] Crous PW, Wingfield MJ, Schumacher RK et al. (2020) New and Interesting Fungi. 3. Fungal Syst Evol 6: 157–231. 10.3114/fuse.2020.06.09 PMC7452156 32904192

[B26] Daros-Pawlyta J, Pawlyta A (2023) The concept of legal interest in the context of the possibility of challenging an air protection programme before an administrative court. Remarks on the CJEU judgment of 22 December 2022, C 61/21. Kwartalnik Prawa Międzynarodowego I(I): 217–251. 10.5604/01.3001.0016.3295

[B27] Dissanayake LS, Samarakoon MC, Maharachchikumbura SSN et al. (2024) Exploring the taxonomy and phylogeny of *Sordariomycetes* taxa emphasizing *Xylariomycetidae* in Southwestern China. Mycosphere 15(1): 1675–1793. 10.5943/mycosphere/15/1/15

[B28] de Hoog GS, van Oorschot CAN (1985) Taxonomy of the *Dactylaria* complex, VI. Key to the genera and check-list of epithets. Studies in Mycology 26: 97–121.

[B29] Garrido-Benavent I, de Los Ríos A, Núñez-Zapata J et al. (2023) Ocean crossers: A tale of disjunctions and speciation in the dwarf-fruticose *Lichina* (lichenized *Ascomycota*). Molecular Phylogenetics and Evolution 185: 107829. 10.1016/j.ympev.2023.107829 37247701

[B30] Gazis R, Miadlikowska J, Lutzoni F et al. (2012) Culture-based study of endophytes associated with rubber trees in Peru reveals a new class of *Pezizomycotina*: *Xylonomycetes*. Molecular Phylogenetics and Evolution 65(1): 294–304. 10.1016/j.ympev.2012.06.019 22772026

[B31] Geiser DM, Gueidan C, Miadlikowska J et al. (2006) *Eurotiomycetes*: *Eurotiomycetidae* and *Chaetothyriomycetidae*. Mycologia 98(6): 1053–1064. 10.1080/15572536.2006.11832633 17486980

[B32] Giraldo A, Crous PW, Schumacher RK et al. (2017) The Genera of Fungi—G3: *Aleurocystis*, *Blastacervulus*, *Clypeophysalospora*, Licr*o*stroma, *Neohendersonia* and *Spumatoria*. Mycological Progress 16: 325–348. 10.1007/s11557-017-1270-8

[B33] Gueidan C, Villaseñor CR, de Hoog GS et al. (2008) A rock-inhabiting ancestor for mutualistic and pathogen-rich fungal lineages. Studies in Mycology 61: 111–119. 10.3114/sim.2008.61.11 PMC2610302 19287533

[B34] Haelewaters D, De Kesel A, Gorczak M et al. (2019a) *Laboulbeniales (Ascomycota)* of the Boston Harbor Islands II (and other localities): species parasitizing *Carabidae*, and the *Laboulbenia flagellata* species complex. Northeastern Naturalist 25(sp9): 110–149. 10.1656/045.025.s906

[B35] Haelewaters D, Pfliegler WP, Gorczak M et al. (2019b) Birth of an order: Comprehensive molecular phylogenetic study excludes *Herpomyces* (*Fungi*, *Laboulbeniales*) from *Laboulbeniales*. Molecular Phylogenetics and Evolution 133: 286–301. 10.1016/j.ympev.2019.01.007 30625361

[B36] Hernández-Restrepo M, Gené J, Castañeda Ruíz RF et al. (2017) Phylogeny of saprobic microfungi from Southern Europe. Studies in Mycology 86: 53–97. 10.1016/j.simyco.2017.05.002 PMC5470572 28626275

[B37] Hongsanan S, Hyde KD (2017) Phylogenetic placement of *Micropeltidaceae*. Mycosphere 8(10): 1930–1942. 10.5943/mycosphere/8/10/15

[B38] Jaklitsch WM, Gardiennet A, Voglmayr H et al. (2016) Resolution of morphology-based taxonomic delusions: *Acrocordiella*, *Basiseptospora*, *Blogiascospora*, *Clypeosphaeria*, *Hymenopleella*, *Lepteutypa*, *Pseudapiospora*, *Requienella*, *Seiridium* and *Strickeria*. Persoonia 37: 82–105. 10.3767/003158516X690475 PMC5238940 28100927

[B39] Jaklitsch WM, Voglmayr H (2012) Phylogenetic relationships of five genera of *Xylariales* and *Rosasphaeria* gen. nov. (*Hypocreales*). Fungal Diversity 52: 75–98. 10.1007/s13225-011-0104-2

[B40] James TY, Kauff F, Schoch CL et al. (2006) Reconstructing the early evolution of *Fungi* using a six-gene phylogeny. Nature 443(7113): 818–822. 10.1038/nature05110 17051209

[B41] Jeewon R, Liew EC, Hyde KD et al. (2003) Molecular systematics of the *Amphisphaeriaceae* based on cladistic analyses of partial LSU rDNA gene sequences. Mycological Research 107(Pt 12): 1392–1402. 10.1017/s095375620300875x 15000240

[B42] Konta S, Hongsanan S, Tibpromma S et al. (2016) An advance in the endophyte story: *Oxydothidaceae* fam. nov. with six new species of *Oxydothis*. Mycosphere 7(9): 1425–1446. 10.5943/mycosphere/7/9/15

[B43] Kurtzman CP, Robnett CJ (2013) Relationships among genera of the *Saccharomycotina (Ascomycota)* from multigene phylogenetic analysis of type species. FEMS Yeast Research 13(1): 23–33. 10.1111/1567-1364.12006 22978764

[B44] Kusari S, Lamshöft M, Spiteller M (2009) *Aspergillus fumigatus* Fresenius, an endophytic fungus from *Juniperus communis* L. Horstmann as a novel source of the anticancer pro-drug deoxypodophyllotoxin. Journal of Applied Microbiology 107(3): 1019–1030. 10.1111/j.1365-2672.2009.04285.x 19486398

[B45] Li Q, Kang JC, Hyde KD (2015) A multiple gene genealogy reveals the phylogenetic placement of *Iodosphaeria tongrenensis* sp. nov. in *Iodosphaeriaceae (Xylariales)*. Phytotaxa 234(2): 121–132. 10.11646/phytotaxa.234.2.2

[B46] Li Y, Jeewon R, Hyde KD et al. (2006) Two new species of nematode-trapping fungi: relationships inferred from morphology, rDNA and protein gene sequence analyses. Mycological Research 110(Pt 7): 790–800. 10.1016/j.mycres.2006.04.011 16876699

[B47] Lin CG, Dai DQ, Bhat DJ et al. (2017) *Subsessila turbinata* gen. et. sp. nov. (*Beltraniaceae*), a Beltrania-like fungus from Thailand. Mycological Progress 16: 393–401. 10.1007/s11557-017-1279-z

[B48] Liu H, Choi H, Paul NC et al. (2025) Discovering fungal communities in roots of *Zoysia japonica* and characterising novel species and their antifungal activities. IMA Fungus 16: e138479. 10.3897/imafungus.16.138479 PMC11881003 40052078

[B49] Lombard L, van der Merwe NA, Groenewald JZ et al. (2015) Generic concepts in *Nectriaceae*. Studies in Mycology 80: 189–245. 10.1016/j.simyco.2014.12.002 PMC4779799 26955195

[B50] Lumbsch HT, Schmitt I, Lindemuth R et al. (2005) Performance of four ribosomal DNA regions to infer higher-level phylogenetic relationships of inoperculate euascomycetes (*Leotiomyceta*). Molecular Phylogenetics and Evolution 34(3): 512–524. 10.1016/j.ympev.2004.11.007 15683926

[B51] Lutzoni F, Kauff F, Cox CJ et al. (2004) Assembling the fungal tree of life: progress, classification, and evolution of subcellular traits. American Journal of Botany 91(10): 1446–1480. 10.3732/ajb.91.10.1446 21652303

[B52] Lutzoni F, Pagel M, Reeb V (2001) Major fungal lineages are derived from lichen symbiotic ancestors. Nature 411(6840): 937–940. 10.1038/35082053 11418855

[B53] Marasinghe DS, Samarakoon MC, Hongsanan S et al. (2019) *Iodosphaeria honghense* sp. nov. (*Iodosphaeriaceae*, *Xylariales*) from Yunnan Province, China. Phytotaxa 420(4): 273–282. 10.11646/phytotaxa.420.4.3

[B54] Matsushima T (1975) Icones Microfungorum a Matsushima Lectorum 1–209.

[B55] McCune B (2018) Two new species in the *Umbilicaria torrefacta* group from Alaska and the Pacific Northwest of North America. Graphis Scripta 30(6): 65–77.

[B56] McTaggart AR, Grice KR, Shivas RG (2013) First report of *Vialaea minutella* in Australia, its association with mango branch dieback and systematic placement of *Vialaea* in the *Xylariales*. Australasian Plant Disease Notes, Australasian Plant Pathology Society 8(1): 63–66. 10.1007/s13314-013-0096-8

[B57] Miadlikowska J, Kauff F, Hofstetter V et al. (2006) New insights into classification and evolution of the *Lecanoromycetes* (*Pezizomycotina*, *Ascomycota*) from phylogenetic analyses of three ribosomal RNA- and two protein-coding genes. Mycologia 98(6): 1088–1103. 10.1080/15572536.2006.11832636 17486983

[B58] Miller AN, Huhndorf SM (2005) Multi-gene phylogenies indicate ascomal wall morphology is a better predictor of phylogenetic relationships than ascospore morphology in the *Sordariales* (*Ascomycota*, *Fungi*). Molecular Phylogenetics and Evolution 35(1): 60–75. 10.1016/j.ympev.2005.01.007 15737582

[B59] Mi LX, Hu DM, Hyde KD et al. (2026) *Funiliomycetaceae* fam. nov. (*Amphisphaeriales*, *Ascomycota*) accommodating *Funiliomyces*, including *F. jiangxiensis* sp. nov. from *Tetradium ruticarpum* and ten new combinations. IMA Fungus 17: e179140. 10.3897/imafungus.17.179140 PMC12905588 41695783

[B60] Perera RH, Maharachchikumbura SS, Hyde KD et al. (2018) An appendage-bearing coelomycete *Pseudotruncatella arezzoensis* gen. and sp. nov. (*Amphisphaeriales* genera *incertae sedis*) from Italy, with notes on *Monochaetinula*. Phytotaxa 338: 177–188. 10.11646/phytotaxa.338.2.2

[B61] Pérez-Ortega S, Garrido-Benavent I, Grube M et al. (2016) Hidden diversity of marine borderline lichens and a new order of fungi: *Collemopsidiales (Dothideomyceta)*. Fungal Diversity 80: 285–300. 10.1007/s13225-016-0361-1

[B62] Pintos Á, Alvarado P, Planas J et al. (2019) Six new species of *Arthrinium* from Europe and notes about *A. caricicola* and other species found in *Carex* spp. hosts. MycoKeys 49: 15–48. 10.3897/mycokeys.49.32115 PMC6424953 30918449

[B63] Prieto M, Baloch E, Tehler A et al. (2013) Mazaedium evolution in the *Ascomycota (Fungi)* and the classification of mazaediate groups of formerly unclear relationship. Cladistics 29(3): 296–308. 10.1111/j.1096-0031.2012.00429.x 34818827

[B64] Réblová M, Kolařík M, Nekvindová J et al. (2021) Phylogeny, global biogeography and pleomorphism of *Zanclospora*. Microorganisms 9: 706. 10.3390/microorganisms9040706 PMC8066784 33805574

[B65] Réblová M, Seifert KA (2012) Cirrosporium novae-zelandiae, an enigmatic coelomycete with meristem arthroconidia, with ancestors in the *Eurotiomycetes*. Mycologia 104(6): 1315–1324. 10.3852/12-040 22675053

[B66] Reeb V, Lutzoni F, Roux C et al. (2004) Contribution of RPB2 to multilocus phylogenetic studies of the euascomycetes (*Pezizomycotina*, *Fungi*) with special emphasis on the lichen-forming *Acarosporaceae* and evolution of polyspory. Molecular Phylogenetics and Evolution 32(3): 1036–1060. 10.1016/j.ympev.2004.04.012 15288074

[B67] Samarakoon MC, Hyde KD, Maharachchikumbura SSN et al. (2022) Taxonomy, phylogeny, molecular dating and ancestral state reconstruction of *Xylariomycetidae (Sordariomycetes)*. Fungal Diversity 112: 1–88. 10.1007/s13225-021-00495-5

[B68] Samarakoon MC, Liu JK, Hyde KD et al. (2019) Two new species of *Amphisphaeria (Amphisphaeriaceae)* from northern Thailand. Phytotaxa 391(3): 207–217. 10.11646/phytotaxa.391.3.4

[B69] Schneider K, Resl P, Spribille T et al. (2016) Escape from the cryptic species trap: lichen evolution on both sides of a cyanobacterial acquisition event. Molecular Ecology 25(14): 3453–3468. 10.1111/mec.13636 PMC5324663 27037681

[B70] Schoch CL, Kohlmeyer J, Volkmann-Kohlmeyer B et al. (2006a) The halotolerant fungus *Glomerobolus gelineus* is a member of the *Ostropales*. Mycological Research 110(Pt 3): 257–263. 10.1016/j.mycres.2005.10.001 16431093

[B71] Schoch CL, Shoemaker RA, Seifert KA et al. (2006b) A multigene phylogeny of the *Dothideomycetes* using four nuclear loci. Mycologia 98(6): 1041–1052. 10.1080/15572536.2006.11832632 17486979

[B72] Schoch CL, Sung GH, López-Giráldez F et al. (2009a) The *Ascomycota* tree of life: a phylum-wide phylogeny clarifies the origin and evolution of fundamental reproductive and ecological traits. Systematic Biology 58(2): 224–239. 10.1093/sysbio/syp020 20525580

[B73] Schoch CL, Wang Z, Townsend JP et al. (2009b) *Geoglossomycetes cl*. nov., *Geoglossales ord*. nov. and taxa above class rank in the *Ascomycota* Tree of Life. Persoonia 22: 129–138. 10.3767/003158509X461486 PMC2776753 19915689

[B74] Shoemaker RA, Hambleton S, Liu M (2013) *Vialaea insculpta* revisited. North American Fungi 8(10): 1–13.

[B75] Smith GJD, Liew ECY, Hyde KD (2003) The *Xylariales*: a monophyletic order containing 7 families. Fungal Diversity 13: 185–218.

[B76] Suh SO, Houseknecht JL, Gujjari P et al. (2013) *Scheffersomyces parashehatae* f.a., sp. nov., *Scheffersomyces xylosifermentans* f.a., sp. nov., *Candida broadrunensis* sp. nov. and *Candida manassasensis* sp. nov., novel yeasts associated with wood-ingesting insects, and their ecological and biofuel implications. International Journal of Systematic and Evolutionary Microbiology 63(Pt 11): 4330–4339. 10.1099/ijs.0.053009-0 24014624

[B77] Spatafora JW, Sung GH, Johnson D et al. (2006) A five-gene phylogeny of *Pezizomycotina*. Mycologia 98(6): 1018–1028. 10.1080/15572536.2006.11832630 17486977

[B78] Summerell BA, Groenewald JZ, Carnegie A et al. (2006) Eucalyptus microfungi known from culture. 2. *Alysidiella*, *Fusculina* and *Phlogicylindrium* genera nova, with notes on some other poorly known taxa. Fungal Diversity 23: 323–350.

[B79] Swe A, Jeewon R, Pointing SB et al. (2008) Taxonomy and molecular phylogeny of *Arthrobotrys mangrovispora*, a new marine nematode-trapping fungal species. Botanica Marina 51(4): 331–338. 10.1515/BOT.2008.043

[B80] Tan YP, Shivas RG (2024) Nomenclatural novelties. Index of Australian Fungi 46 (ISBN 978-1-7636439-5-6): 1–17. 10.5281/zenodo.13905938

[B81] Turland NJ, Wiersema JH, Barrie FR et al. (2025) International Code of Nomenclature for algae, fungi, and plants (Madrid Code). Regnum Vegetabile 162. Chicago: University of Chicago Press. 10.7208/chicago/9780226839479.001.0001

[B82] Van Vooren N (2020) Validation de *Peziza martinicensis* sp. nov.(*Pezizales*). Ascomycete.org 12(2): 57–60. 10.25664/ART-0298

[B83] Voglmayr H, Fournier J, Jaklitsch WM et al. (2019) Two new classes of *Ascomycota*: *Xylobotryomycetes* and *Candelariomycetes*. Persoonia 42: 36–49. 10.3767/persoonia.2019.42.02 PMC6712537 31551613

[B84] Vu D, Groenewald M, de Vries M et al. (2019) Large-scale generation and analysis of filamentous fungal DNA barcodes boosts coverage for kingdom fungi and reveals thresholds for fungal species and higher taxon delimitation. Studies in Mycology 92: 135–154. 10.1016/j.simyco.2018.05.001 PMC6020082 29955203

[B85] Wang XW, Houbraken J, Groenewald JZ et al. (2016) Diversity and taxonomy of *Chaetomium* and chaetomium-like fungi from indoor environments. Studies in Mycology 84: 145–224. 10.1016/j.simyco.2016.11.005 PMC5226397 28082757

[B86] Wedin M, Wiklund E, Crewe A et al. (2005) Phylogenetic relationships of *Lecanoromycetes (Ascomycota)* as revealed by analyses of mtSSU and nLSU rDNA sequence data. Mycological Research 109(Pt 2): 159–172. 10.1017/s0953756204002102 15839100

[B87] Wood AR, Damm U, van der Linde EJ et al. (2016) Finding the missing link: Resolving the *Coryneliomycetidae* within *Eurotiomycetes*. Persoonia 37: 37–56. 10.3767/003158516X689800 PMC5315291 28232760

[B88] Xie L, Chen YL, Long YY et al. (2019) Three new species of *Conlarium* from sugarcane rhizosphere in southern China. MycoKeys 56: 1–11. 10.3897/mycokeys.56.35857 PMC6626063 31327928

[B89] Yang CL, Xu XL, Liu YG et al. (2019) First report of bamboo blight disease caused by *Arthrinium pseudoparenchymaticum* on *Dendrocalamus latiflorus* in Sichuan, China. Plant Disease 103(6): 1411. 10.1094/PDIS-01-19-0029-PDN

[B90] Zhang JY, Hyde KD, Bao DF et al. (2025) A worldwide checklist and morpho-molecular systematics of fungi associated with pteridophytes. Fungal Diversity 132: 151–423. 10.1007/s13225-025-00554-1

[B91] Zare R, Gams W (2016) More white verticillium-like anamorphs with erect conidiophores. Mycol Progress 15: 993–1030. 10.1007/s11557-016-1214-8

